# Duration Comparisons for Vision and Touch Are Dependent on Presentation Order and Temporal Context

**DOI:** 10.3389/fnint.2021.664264

**Published:** 2021-06-23

**Authors:** Yi Gao, Kamilla N. Miller, Michael E. Rudd, Michael A. Webster, Fang Jiang

**Affiliations:** Integrative Neuroscience Program, University of Nevada, Reno, Reno, NV, United States

**Keywords:** central tendency, duration perception, crossmodal, presentation order, temporal context

## Abstract

Integrating visual and tactile information in the temporal domain is critical for active perception. To accomplish this, coordinated timing is required. Here, we study perceived duration within and across these two modalities. Specifically, we examined how duration comparisons within and across vision and touch were influenced by temporal context and presentation order using a two-interval forced choice task. We asked participants to compare the duration of two temporal intervals defined by tactile or visual events. Two constant standard durations (700 ms and 1,000 ms in ‘shorter’ sessions; 1,000 ms and 1,500 ms in ‘longer’ sessions) were compared to variable comparison durations in different sessions. In crossmodal trials, standard and comparison durations were presented in different modalities, whereas in the intramodal trials, the two durations were presented in the same modality. The standard duration was either presented first (<sc>) or followed the comparison duration (<cs>). In both crossmodal and intramodal conditions, we found that the longer standard duration was overestimated in <cs> trials and underestimated in <sc> trials whereas the estimation of shorter standard duration was unbiased. Importantly, the estimation of 1,000ms was biased when it was the longer standard duration within the shorter sessions but not when it was the shorter standard duration within the longer sessions, indicating an effect of temporal context. The effects of presentation order can be explained by a central tendency effect applied in different ways to different presentation orders. Both crossmodal and intramodal conditions showed better discrimination performance for <sc> trials than <cs> trials, supporting the Type B effect for both crossmodal and intramodal duration comparison. Moreover, these results were not dependent on whether the standard duration was defined using tactile or visual stimuli. Overall, our results indicate that duration comparison between vision and touch is dependent on presentation order and temporal context, but not modality.

## Introduction

Timing and time perception play a crucial role in experiencing and planning activities in our daily life. The ability to discriminate between temporal intervals of the same and different modalities is essential to form a coherent perception of the world. How do we compare durations of different lengths? Recent studies of duration estimation have mostly used a manual reproduction task or a two-interval forced choice task (2IFC; Vierordt, 1868; Allan, 1977; Jamieson, 1977; Harrington et al., 2004; Jazayeri and Shadlen, 2010; Gu and Meck, 2011; Bratzke and Ulrich, 2019). Reproduction tasks may involve a different system of motor timing rather than the perceptual timing measured in 2IFC tasks (see Lewis and Miall, 2003 for review). In a common 2IFC paradigm, participants are asked to compare two sequentially presented time intervals one of which is fixed, representing the standard duration and the other is varying representing the comparison duration. Stimuli can be presented in either of two orders: standard duration followed by comparison duration (<sc>) or comparison duration followed by standard duration (<cs>). Importantly, subjective time is not a direct reflection of objective time and presentation order can affect the perceived duration. The influence of presentation order on perceived duration is quantified by the point of subjective equality (PSE), where the comparison duration is perceived as the same length as the standard duration. The effect of presentation order on PSE is called the time-order error (TOE, also called Type A effect, Fechner, 1860; Allan, 1977; Hellström, 1979, 1985). TOE is negative when the first duration is underestimated and the second duration is overestimated, and positive for the opposite pattern. Presentation order can also influence duration discrimination performance in that better discriminability is obtained when the standard duration is presented first than when the comparison duration is presented first (standard-position effect, also known as constant-position effect or Type B effect; Rammsayer and Wittkowski, 1990; Grondin and McAuley, 2009; Ulrich and Vorberg, 2009).

In addition to presentation order, there are many other factors influencing duration comparison. For example, duration perception can also be affected by stimulus context in addition to presentation order (Vierordt, 1868; Jones and McAuley, 2005; Jazayeri and Shadlen, 2010). Furthermore, duration judgments of current stimuli can be influenced by the duration distribution of previous events. A representative example is the well-known Vierordt’s law: shorter durations are overestimated and longer durations underestimated in a sequentially presented duration set. Vierordt’s law has been studied since the 1860s (Vierordt, 1868; Lejeune and Wearden, 2009). This effect has been explained by a general central tendency effect in which participants tend to bias their judgments toward the mean of the stimulus distribution (Hollingworth, 1910).

It is known that perceived duration can also be influenced by the modality it is presented in. Several previous studies have shown that auditory duration is overestimated while visual duration is underestimated when they are mixed in the same session (Penney et al., 1998, 2000; Penney and Tourret, 2005). Compared to studies of visual and auditory timing, studies comparing durations of touch and other modalities are scant. Earlier studies showed that tactile durations were perceived approximately the same as auditory durations (Ehrensing and Lhamon, 1966; Hawkes et al., 1977). When comparing visual and tactile durations, there seems to be less consistency. With filled intervals (i.e., a continuous stimulus throughout the duration), visual stimuli appear to last longer than tactile stimuli of the same duration when tested with a reproduction task (Tomassini et al., 2011). In contrast, one study showed that visual intervals needed to be about 8% longer to match the tactile intervals with random presentation order in a 2IFC task (Van Erp and Werkhoven, 2004). Whether presentation order confounded these results remained unknown, however, as the authors did not analyze PSEs for <sc> and <cs> trials separately.

The effects of stimulus order and temporal context are mostly studied by duration comparisons within the same modality. For example, Ellinghaus et al. (2018) found a Type B effect in both visual and auditory modalities. It is unknown whether there is a Type B when comparing durations across modalities. In terms of temporal contextual modulation, Bratzke and Ulrich (2019) found a central tendency effect both across and within visual and auditory modalities using a reproduction task while Rhodes et al. (2018) reached an opposite conclusion.

Given the importance of visual and tactile duration perception for a coherent perception of the world and to plan for coordinated behaviors (Keetels and Vroomen, 2007; Medina et al., 2018), the current study aimed to examine whether duration discrimination within and across modalities (vision and touch) were influenced by presentation orderand temporal context in a similar way. To examine this, we explored the properties of temporal judgments when participants compared temporal intervals presented in two modalities (visual and tactile) within different sessions (shorter vs. longer) and presentation orders (<sc> vs. <cs>). Importantly, the same standard duration was imbedded in both shorter and longer sessions to study the effect of temporal context.

## Materials and Methods

### Participants

A total of 18 undergraduate and graduate students at the University of Nevada, Reno participated in this study. Nine naïve participants (aged from 22 to 30, mean age: 26.67, standard deviation: 2.40, six female and three male) participated in Experiment 1. Nine naïve participants (aged 19–29, mean: 24.78, standard deviation: 3.83, four female and five male) participated in Experiment 2. All participants were right-handed and reported normal or corrected-to-normal vision and normal hearing. Participants provided signed informed consent before the experiments and were financially compensated. The protocols were reviewed and approved by the Institutional Review Board at the University of Nevada, Reno.

### Apparatus and Stimuli

The visual stimuli were displayed on a calibrated and gamma-corrected Display++ LCD monitor (Cambridge Research Systems). The refresh rate of the monitor was 120 Hz. The mean luminance of the stimuli was 46.2 cd/m^2^, measured with a PR-655 spectroradiometer. Participants sat at 70 cm from the monitor. The visual stimuli were presented at the center of a gray background on the display monitor. The stimuli were white disks (maximum luminance: 91.8 cd/m^2^), 4-deg diameter in visual angle, and smoothed with a Gaussian envelope having a 0.8-deg standard deviation unless otherwise stated. The visual stimuli were presented as brief 25 ms flashes. Tactile stimuli were delivered by PiezoTac (Engineering Acoustics, Inc.). The tactile stimuli also lasted 25 ms. They were felt by the subjects as vibrations. Each had a vibration frequency of 50 Hz and an amplitude gain of 255 (maximum of the device) unless otherwise stated. Note that we used empty intervals instead of filled intervals because pulse durations of less than 600 ms are suggested when using the PiezoTac device. The amplitude gain represents the peak-to-peak displacement of the tactors, with a larger number representing a larger displacement. The amplitude of the vibration was presented at a level that can be easily detected by participants. Participants were asked to put the tip of their right index finger on the tactile pad and tactile vibrations were delivered to the index finger. They were asked to make a response with their left hand using a keyboard.

### General Procedure

**Figure 1:**
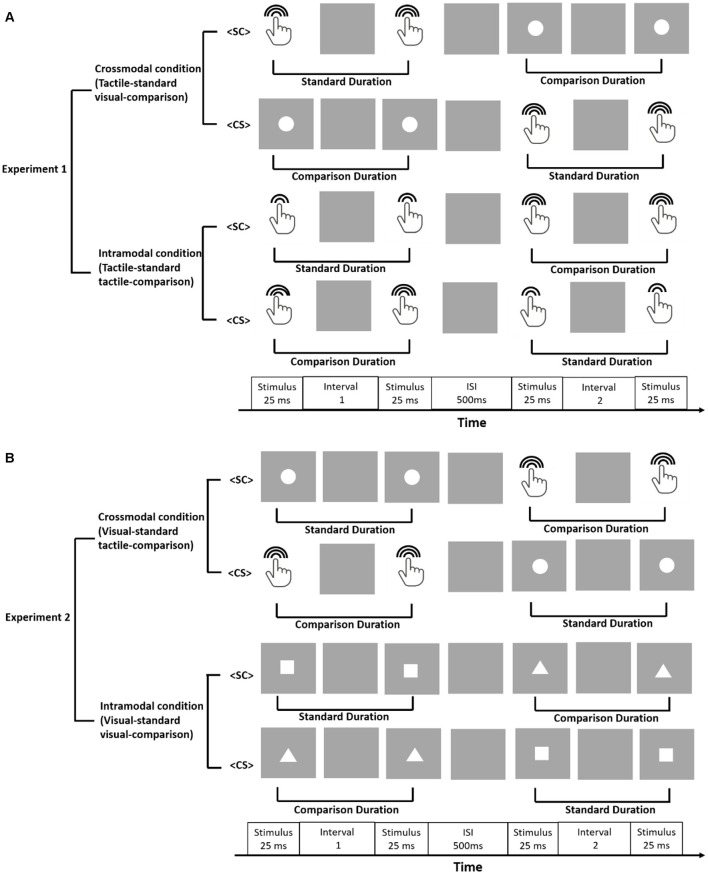
Experimental paradigm. Panel **(A)** illustrates the setup for Experiment 1. The crossmodal condition of Experiment 1 was set as tactile-standard visual-comparison while the intramodal condition was set as tactile-standard tactile-comparison. Panel **(B)** illustrates the setup for Experiment 2. The crossmodal condition of Experiment 2 was set as visual-standard tactile-comparison while the intramodal condition was set as visual-standard visual-comparison.

Duration discrimination was measured with a 2IFC task (see Figure 1 for paradigms and conditions). A given duration corresponded to the time interval between either two visual flashes or two tactile vibrations. Each trial started with the presentation of a blank gray screen with a random duration between 1,500 ms and 2,000 ms. The blank screen was followed by two visual flashes or two tactile vibrations with the temporal delay between them defining the first temporal interval. After a 500 ms blank gray screen, another two visual flashes or two tactile vibrations were presented with the temporal delay between the two defining the second temporal interval. Participants were asked to press the left-arrow key if the first interval was longer and press the right-arrow key if the second interval was longer. In the crossmodal condition, the two stimuli were delivered in different modalities (vision and touch), while in the intramodal condition the two intervals were delivered in the same modality (vision or touch). Crossmodal and intramodal conditions were tested in different sessions. Participants wore ear plugs throughout the experiment to avoid the influence of sound from the tactile vibration.

### Experimental Design

We conducted two experiments, with Experiment 1 presenting standard duration in tactile modality and Experiment 2 presenting the standard duration in visual modality. Each experiment consisted of two conditions (crossmodal vs. intramodal, Figure 1). In the crossmodal condition of Experiment 1, the standard duration was defined by the interval between two tactile vibrations, and the comparison duration was defined by the interval between two visual flashes. In the intramodal condition, the standard and comparison duration were both defined by the intervals between tactile vibrations with different vibration amplitudes. Standard and comparison durations were randomly paired with weak (amplitude gain of 75) and strong (amplitude gain of 200) vibrations across participants but were consistent for each participant throughout the whole session. In the crossmodal condition of Experiment 2, the visual duration served as the standard and the tactile duration served as the comparison. In the intramodal condition, the standard and comparison durations were defined by the intervals between visual flashes of different shapes. Standard and comparison intervals were randomly paired with square and triangle shapes across participants but were consistent for each participant throughout the experiment. The heights and widths of the squares and triangles were all 4-deg. Since the durations were defined by empty intervals in our experiment, participants may mistakenly group the markers if the same shape/intensity was used. Thus, different visual shapes/vibration intensities were used to help participants to group stimuli into two intervals. Similarly, Bratzke and Ulrich (2019) modulated sound intensity and visual object shape (a relatively slight variation in the stimulus) in their study.

For each condition, there were four sessions: two for shorter duration distributions (‘shorter’ sessions) and two for longer duration distributions (‘longer’ sessions). In the two “shorter” sessions, the two standard durations presented in each session were 700 ms and 1,000 ms. For each short session, there were nine comparison intervals in equal steps, ranging from 210 ms to 1,190 ms for the 700 ms standard duration and from 300 ms to 1,700 ms for the 1,000 ms standard duration. In the two “longer” sessions, the two standard durations presented in each session were 1,000 ms and 1,500 ms. Similarly, there were nine comparison intervals in equal steps for each longer session, ranging from 300 ms to 1,700 ms for the 1,000 ms standard duration and from 800 ms to 2,200 ms for the 1,500 ms standard duration. In half of the trials, the standard temporal intervals were presented first (<sc>) and in the other half, comparison intervals were presented first (<cs>). The two types of trials (<sc> vs. <cs>) were randomized within a session. Each comparison level had 10 trials per presentation order per standard duration in a session, resulting in a total of 360 trials per session (10 trials × 9 comparison levels × 2 standard durations × 2 presentation order). Participants were given a 1 min break after every 60 trials and were tested in only one session per day. The presentation order of crossmodal vs. intramodal condition was counterbalanced across participants and, within each condition, the order between “shorter” and “longer” sessions was also counterbalanced across participants.

### Analysis

The percentage of “comparison interval longer” judgments was calculated as a function of comparison duration. A psychometric function was then obtained by fitting the percentage “longer” responses with a cumulative Gaussian function. PSEs for individual participants were derived from the psychometric function. The PSE is the comparison duration for which there was an equal chance of answering that the comparison or standard duration was longer. Bias was defined as the difference between the measured PSE and the veridical standard duration. Just-noticeable difference (JND) was calculated as half of the difference between the durations at which the comparison was judged to be longer 75% and 25% of the time. A Weber fraction was then calculated by dividing the JND by the corresponding standard duration.

Bayesian statistics were implemented in JASP (Version 0.14., Computer software). Bayesian statistics are equally valid for all sample sizes (Wagenmakers et al., 2018). Following the suggestions of Jeffreys (1961), Bayes Factors with a BF_10_ were interpreted as following: <1: no evidence, 1–3: anecdotal evidence, 3–10: moderate evidence, 10–30: strong evidence, 30–100: very strong evidence, >100: extreme evidence supporting the alternative hypothesis. Due to the number of predictors, the model numbers were too large to be individually compared. Thus, Bayes factors for inclusion were calculated based on Bayesian model averaging (Etz and Wagenmakers, 2017). The Bayes factor for inclusion indicates how much more likely the data were under the model including the predictor than under the model without the predictor. To correct for multiplicity, the priors for t-tests were corrected for the null hypotheses through the formula [(1− *p*(*H*_0_)^(2/*k*))/(*p*(*H*_0_)^(2/*k*))], where p(H_0_) equaled 0.5 and k represented number of conditions (Westfall, 1997; De Jong et al., 2019; van den Bergh et al., 2019).

## Results

### Experiment 1

**Figure 2:**
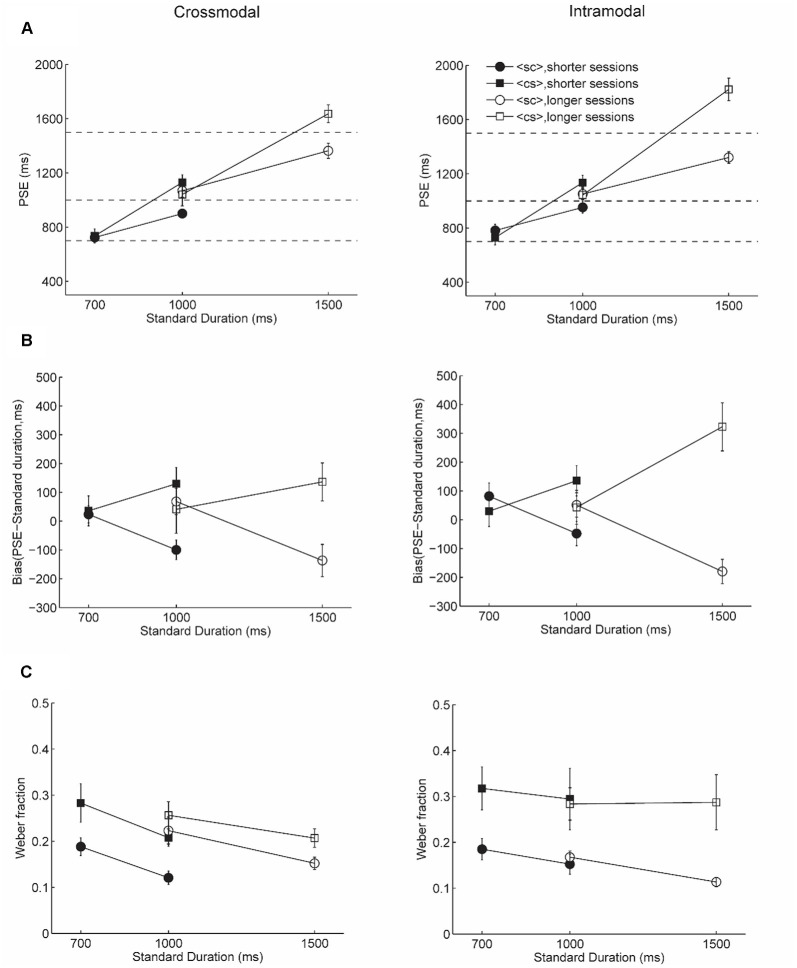
Results of Experiment 1. Panels **(A,B,C)** show the point of subjective equality (PSE), bias, and Weber fraction, respectively. Left panels show results for crossmodal condition and right panels show results for intramodal condition. Solid symbols show results for the shorter sessions and empty symbols show results for the longer sessions. Circles show results for <sc> trials and squares show results for <cs> trials. The dashed lines in **(A)** show the veridical standard duration.

In Experiment 1, we examined duration discrimination when a tactile temporal interval was used as the standard duration and a visual temporal interval was used as the comparison duration. Figure 2 shows results for both crossmodal and comparison conditions, with PSEs shown in Figure 2A and bias (PSE- veridical standard duration) shown in Figure 2B (also see Supplementary Figure 1 for individual data of bias).

We ran a four-way Bayesian repeated measures analysis of variance (ANOVA) to compare the bias, as a function of standard duration (shorter vs. longer standard duration in each session), presentation order (<cs> vs. <sc>), session (shorter vs. longer session), and condition (crossmodal vs. intramodal). A Bayesian factor (BF_10_) of 4.0*10^8^ provided extreme evidence for a significant interaction between presentation order and standard duration, a main effect of presentation order, and a main effect of standard duration (see Supplementary Table 1 for model comparisons). *Post hoc* analysis showed extreme evidence for larger bias for <cs> trials compared to those for <sc> trials for longer standard durations (uncorrected *BF*_10_ = 118,293.3, adjusted posterior odds = 48,998.7, bias for <sc> trials: Mean = −115.9 ms, *SD* = 136.1, bias for <sc> trials: Mean = 181.1 ms, *SD* = 205.7). Importantly, the longer standard durations in both sessions (i.e., 1,000 ms in shorter sessions and 1,500 ms in longer sessions) were underestimated for <sc> trials and overestimated for <cs> trials in both crossmodal and intromodal conditions. There was no evidence supporting a difference of duration estimation between <sc> trials and <cs> trials for shorter standard durations (uncorrected *BF*_10_ = 0.2, adjusted posterior odds = 0.08, bias for <sc> trials: Mean = 56.3 ms, *SD* = 120.9, bias for <sc> trials: Mean = 37.4 ms, SD = 181.1). Consistent with the Bayesian model comparison results, the Bayesian factor for inclusion strongly supports a model that includes presentation order (*BF*_inclusion_ = 6.5*10^7^), followed by the interaction between presentation order and standard duration (BF_inclusion_ = 34,013.3), and then standard duration (BF_inclusion_ = 4,635.8, see Table 1). Notably, the effect analysis of session (BF_inclusion_ = 0.07) and condition (BF_inclusion_ = 0.08) provided substantial evidence that session and modality condition did not have an influence on the bias.

**Table 1:** Bayes factors for inclusion calculated based on Bayesian model averaging for PSEs of Experiment 1.

Effects	P(incl)	P(excl)	P(incl|data)	P(excl|data)	BF_incl_
**Analysis of effects**
presentation order	0.886	0.114	1.000	1.976e-9	6.496e+7
session	0.886	0.114	0.347	0.653	0.068
standard duration	0.886	0.114	1.000	2.769e-5	4,635.849
condition	0.886	0.114	0.373	0.627	0.076
presentation order * standard duration	0.503	0.497	1.000	2.905e-5	34,013.281
presentation order * condition	0.503	0.497	0.092	0.908	0.100
session * presentation order	0.503	0.497	0.178	0.822	0.214
session * standard duration	0.503	0.497	0.101	0.899	0.112
session * condition	0.503	0.497	0.030	0.970	0.031
standard duration * condition	0.503	0.497	0.104	0.896	0.115
presentation order * standard duration * condition	0.120	0.880	0.010	0.990	0.072
session * presentation order * standard duration	0.120	0.880	0.035	0.965	0.268
session * presentation order * condition	0.120	0.880	0.004	0.996	0.028
session * standard duration * condition	0.120	0.880	0.001	0.999	0.007
session * presentation order * standard duration * condition	0.006	0.994	1.448e-5	1.000	0.002

*P (incl) and P (excl) represent the priors of including and excluding the predictor before seeing the model. P (incl|data) and P (excl|data) represent the posteriors of including and excluding the predictor after seeing the model. BF_incl_ represents how much more likely the data are under the model including the predictor than under the model excluding the predictor. Abbreviation: PSE, point of subjective equality*.

Figure 2C shows Weber fractions. A four-way Bayesian repeated measures analysis of variance was run to compare the Weber fractions, as a function of standard duration (shorter vs. longer standard duration in each session), presentation order (<cs> vs. <sc>), session (shorter vs. longer session), and condition (crossmodal vs. intramodal). There was extreme evidence (BF_10_ = 6.9*10^8^) in favor of a model for main effect of presentation order, main effect of standard duration, and main effect of condition, and interaction between presentation order and condition (see Supplementary Table 2 for model comparisons). *Post hoc* analysis provided evidence for a smaller Weber fraction for <sc> trials than <cs> trials for both crossmodal and intramodal conditions (crossmodal: uncorrected BF_10_ = 134.0, adjusted posterior odds = 55.5, <sc> trials: Mean = 0.17, SD = 0.07, <cs> trials: Mean = 0.24, SD = 0.09, intramodal: BF_10_ = 8901.8, adjusted posterior odds = 3687.1, <sc> trials: Mean = 0.16, *SD* = 0.06, <cs> trials: Mean = 0.30, *SD* = 0.16). Weber fractions for the longer standard duration were smaller than those for shorter standard durations irrespective of session and modality condition, suggesting overall better performance for longer standard durations (BF_10_ = 63.6, longer standard duration: Mean = 0.19, *SD* = 0.12; longer standard duration: Mean = 0.24, SD = 0.10). Bayes factor for inclusion (Table 2) indicates that the evidence most strongly supports the inclusion of the presentation order (BF_inclusion_ = 4.5*10^7^), and moderate evidence to include standard duration (BF_inclusion_ = 3.7), and anecdotal evidence for interaction between presentation order and condition (BF_inclusion_ = 1.4). Notably, the effect analysis of session (BF_inclusion_ = 0.06) or condition (BF_inclusion_ = 0.4) provided substantial evidence that session and condition did not have an influence on the Weber fractions.

**Table 2:** Bayes factors for inclusion calculated based on Bayesian model averaging for Weber fractions of Experiment 1.

Effects	P(incl)	P(excl)	P(incl|data)	P(excl|data)	BF_incl_
**Analysis of effects**
presentation order	0.886	0.114	1.000	2.843e-9	4.515e+7
session	0.886	0.114	0.331	0.669	0.064
standard duration	0.886	0.114	0.967	0.033	3.735
condition	0.886	0.114	0.776	0.224	0.445
presentation order * standard duration	0.503	0.497	0.265	0.735	0.357
presentation order * condition	0.503	0.497	0.584	0.416	1.389
session * presentation order	0.503	0.497	0.070	0.930	0.075
session * standard duration	0.503	0.497	0.059	0.941	0.062
session * condition	0.503	0.497	0.077	0.923	0.082
standard duration * condition	0.503	0.497	0.263	0.737	0.353
presentation order * standard duration * condition	0.120	0.880	0.014	0.986	0.105
session * presentation order * standard duration	0.120	0.880	0.001	0.999	0.009
session * presentation order * condition	0.120	0.880	0.006	0.994	0.043
session * standard duration * condition	0.120	0.880	0.002	0.998	0.012
session * presentation order * standard duration * condition	0.006	0.994	1.277e-6	1.000	2.120e-4

*P (incl) and P (excl) represent the priors of including and excluding the predictor before seeing the model. P (incl|data) and P (excl|data) represent the posteriors of including and excluding the predictor after seeing the model. BF_incl_ represents how much more likely the data are under the model including the predictor than under the model excluding the predictor*.

To summarize, we found that duration comparisons between vision and touch were influenced by presentation order and standard duration, but not session and modality. Specifically, we found an underestimation of longer standard durations in <sc> trials and overestimation of longer standard durations in <cs> trials, regardless of the modality condition and whether trials were imbedded in shorter or longer sessions. Better discrimination performance was found for <sc> trials than <cs> trials and for longer standard durations than shorter standard durations.

### Experiment 2

**Figure 3:**
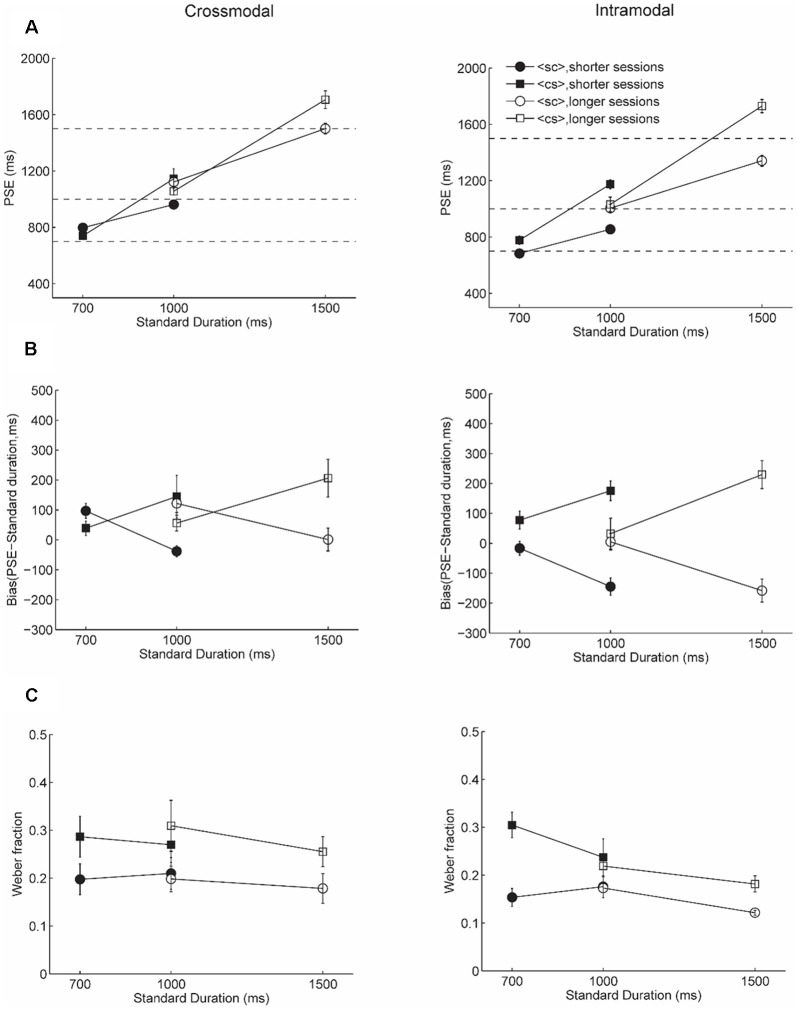
Results of Experiment 2. Panels **(A,B,C)** show the PSE, bias, and Weber fraction, respectively. Left panels show results for the crossmodal condition and right panels show results for intramodal condition. Solid symbols show results for the shorter sessions and empty symbols shows results for the longer sessions. Circles show results for <sc> trials and squares show results for <cs> trials. The dashed lines in **(A)** show the veridical standard duration.

In Experiment 1, the standards were always presented in the tactile domain. Experiment 2 was conducted to verify that our results were not specific to the standards being tactile. In the crossmodal condition of Experiment 2, a new group of participants were asked to judge tactile comparison durations to visual standard durations (visual-standard tactile-comparison). In the intramodal condition of Experiment 2, the participants was asked to compare a pair of visual durations defined by different shapes (visual-standard visual-comparison). We predicted that the findings in Experiment 1 would be replicated in Experiment 2, indicating that our results were not dependent on which modality was being used for the standard durations. Figure 3A plots PSEs and Figure 3B plots bias for Experiment 2 (also see Supplementary Figure 2 for individual data of bias).

A four-way Bayesian repeated measures ANOVA for the bias showed a Bayesian factor of 3.3*10^15^ for the interaction between presentation order and standard duration, condition and standard duration, main effect of presentation order, standard duration, and condition (see Supplementary Table 3). *Post hoc* analysis showed that for longer standard durations, bias for <cs> (Mean = 189.1 ms, SD = 115.2) was significantly larger than that for <sc> trials (Mean = −84.8 ms, SD = 161.7, uncorrected BF_10_ = 389978.1, adjusted posterior odds = 73,783.9) while there was no evidence supporting differences between different presentation orders for shorter standard durations (uncorrected BF_10_ = 0.2, adjusted posterior odds = 0.04, <sc>: Mean = 51.9 ms, SD = 97.4, <cs>: SD = 51.2 ms, SD = 101.8). The bias in duration estimation for <cs> trials (Mean = 128.7 ms, SD = 142.7) was larger than <sc> trials (Mean = −78.5 ms, SD = 114.2) for intramodal condition (uncorrected BF_10_ = 2770.6, adjusted posterior odds = 524.2) but not for crossmodal condition (uncorrected BF_10_ = 0.7, adjusted posterior odds = 0.1, <sc>: Mean = 45.6 ms, SD = 106.8, <cs>: Mean = 111.6 ms, SD = 160.6). Table 3 showed Bayes factor for inclusion based on Bayesian model averaging. Consistent with the Bayesian model comparison results, the evidence most strongly supports the inclusion of the presentation order (BF_inclusion_ = ∞), the interaction between presentation order and standard duration (BF_inclusion_ = 1.2*10^8^), standard duration (BF_inclusion_ = 2.0*10^7^), followed by condition (BF_inclusion_ = 121.4) and the interaction between condition and presentation order (BF_inclusion_ = 108.9). Notably, the effect analysis of session (BF_inclusion_ = 0.08) provided substantial evidence that session did not have an influence on the bias.

**Table 3:** Bayes factors for inclusion calculated based on Bayesian model averaging for PSEs of Experiment 2.

Effects	P(incl)	P(excl)	P(incl|data)	P(excl|data)	BF_incl_
**Analysis of effects**
presentation order	0.886	0.114	1.000	0.000	∞
session	0.886	0.114	0.396	0.604	0.084
standard duration	0.886	0.114	1.000	6.535e-9	1.964e+7
condition	0.886	0.114	0.999	0.001	121.43612.
session * presentation order	0.503	0.497	0.093	0.907	0.101
session * standard duration	0.503	0.497	0.111	0.889	0.124
session * condition	0.503	0.497	0.105	0.895	0.116
standard duration * presentation order	0.503	0.497	1.000	8.111e-9	1.218e+8
standard duration * condition	0.503	0.497	0.257	0.743	0.341
condition * presentation order	0.503	0.497	0.991	0.009	108.90012
session * standard duration * presentation order	0.120	0.880	0.011	0.989	0.079
session * standard duration * condition	0.120	0.880	0.002	0.998	0.016
session * condition * presentation order	0.120	0.880	0.007	0.993	0.052
standard duration * condition * presentation order	0.120	0.880	0.066	0.934	0.521
session * standard duration * condition * presentation order	0.006	0.994	8.707e-6	1.000	0.001

*P (incl) and P (excl) represent the priors of including and excluding the predictor before seeing the model. P (incl|data) and P (excl|data) represent the posteriors of including and excluding the predictor after seeing the model. BF_incl_ represents how much more likely the data are under the model including the predictor than under the model excluding the predictor*.

Figure 3C plots Weber fractions. A four-way Bayesian repeated measures ANOVA showed extreme evidence for a main effect of presentation order, standard duration, session, and modality condition over other models (BF_10_ = 1.5*10^9^). *Post hoc* tests showed extreme evidence for a main effect of presentation order (BF_10,*u*_ = 2.5*e^10^, adjusted posterior odds = 2.3*10^9^). Weber fractions for <sc> trials (Mean = 0.18, SD = 0.08) were smaller than for <cs> trials (Mean = 0.26, SD = 0.11). There was only anecdotal evidence for a main effect of condition (uncorrected BF_10_ = 13.1, adjusted posterior odds = 1.2, intramodal condition: Mean = 0.20, SD = 0.08; crossmodal condition: Mean = 0.24, SD = 0.11) and no evidence for a main effect of session (uncorrected BF_10_ = 2.6, adjusted posterior odds = 0.2) and standard duration (uncorrected BF_10_ = 3.3, adjusted posterior odds = 0.3). Table 4 showed Bayes factor for inclusion based on Bayesian model averaging. The evidence strongly supports the inclusion of the presentation order (BF_inclusion_ = 3.3*10^7^) and condition (BF_inclusion_ = 23.0), but not for standard duration (BF_inclusion_ = 0.6) and session (BF_inclusion_ = 0.7).

**Table 4:** Bayes factors for inclusion calculated based on Bayesian model averaging for Weber fractions of Experiment 2.

Effects	P(incl)	P(excl)	P(incl|data)	P(excl|data)	BF_incl_
**Analysis of effects**
presentation order	0.886	0.114	1.000	3.837e-9	3.346e+7
session	0.886	0.114	0.829	0.171	0.623
standard duration	0.886	0.114	0.852	0.148	0.740
condition	0.886	0.114	0.994	0.006	23.024
session * presentation order	0.503	0.497	0.222	0.778	0.282
session * standard duration	0.503	0.497	0.239	0.761	0.310
session * condition	0.503	0.497	0.394	0.606	0.641
standard duration * presentation order	0.503	0.497	0.348	0.652	0.527
standard duration * condition	0.503	0.497	0.198	0.802	0.244
condition * presentation order	0.503	0.497	0.215	0.785	0.270
session * standard duration * presentation order	0.120	0.880	0.011	0.989	0.079
session * standard duration * condition	0.120	0.880	0.009	0.991	0.063
session * condition * presentation order	0.120	0.880	0.019	0.981	0.146
standard duration * condition * presentation order	0.120	0.880	0.005	0.995	0.039
session * standard duration * condition * presentation order	0.006	0.994	1.291e-5	1.000	0.002

*P (incl) and P (excl) represent the priors of including and excluding the predictor before seeing the model. P (incl|data) and P (excl|data) represent the posteriors of including and excluding the predictor after seeing the model. BF_incl_ represents how much more likely the data are under the model including the predictor than under the model excluding the predictor*.

Thus, Bayesian analysis for Experiment 2 revealed the existence of a larger bias for <cs> trials than <sc> trials for longer standard durations, but not for shorter standard durations in both sessions, with extreme evidence in the intramodal condition but not in the crossmodal condition. There was also anecdotal evidence for a modality effect in that better discrimination was found in the intramodal condition compared to the crossmodal condition. Consistent with Experiment 1, better discrimination performance was found with strong evidence for <sc> trials than <cs> trials.

### Comparison Between Experiment 1 and Experiment 2

We ran a Bayesian ANOVA with a mixed design to compare Experiments 1 and 2. We combined bias and Weber fractions across shorter and longer sessions since the session did not have an influence on bias and Weber fractions in both experiments. A four-way Bayesian repeated measures ANOVA was conducted, with presentation order, standard duration, and modality as within-subjects factors, and experiment as a between-subjects factor. For the bias, a Bayesian factor of 6.5*10^22^ showed extreme evidence for a model including the main effect of presentation order, standard duration, condition, an interaction between presentation order and standard duration, and an interaction between presentation order and condition over other models. Analysis of effects showed extreme evidence to include presentation order (BF_inclusion_ = ∞), the interaction presentation order and duration (BF_inclusion_ = 2.7*10^13^), and standard duration (BF_inclusion_ = 4.0*10^12^). There was only anecdotal evidence for the interaction between presentation order and modality condition (BF_inclusion_ = 1.7). Most importantly, a Bayesian inclusion factor of 0.1 for the experiment provided strong evidence that the bias was influenced similarly between Experiment 1 and 2.

For the Weber fractions, a Bayesian factor of 2.8*10^17^ showed extreme evidence for a model including the main effect of presentation order, standard duration, condition, experiment, and interaction between modality condition and experiment over other models. Analysis of effects showed extreme evidence to include presentation order (BF_inclusion_ = ∞), strong evidence for standard duration (BF_inclusion_ = 29.0), moderate evidence to include the interaction between modality and experiment (BF_inclusion_ = 3.5), and anecdotal evidence to include the interaction between presentation order, modality condition and experiment (BF_inclusion_ = 1.1). This is consistent with results from our early analysis that better performance for intramodal condition compared to the crossmodal condition in Experiment 2 but not in Experiment 1.

To summarize, the bias for duration estimation in both Experiment 1 and 2 was similarly influenced by presentation order and standard duration but not by session and modality. However, the two experiments differed in how Weber fractions were influenced by the modality condition.

## Discussion

Given the importance of temporal integration of visual and tactile information for active perception, we examined duration comparisons between vision and touch under different presentation orders, sessions, and modality conditions. We found that for both crossmodal and intramodal conditions, there was a bias for duration estimation for the longer standard duration but not the shorter standard duration in both shorter and longer sessions. Specifically, the estimation of 1,000 ms was biased when it was the longer standard duration within the shorter sessions but not when it was the shorter standard duration within the longer sessions, indicating the effect of temporal context. Moreover, the direction of the bias depended on the presentation order. We also found that duration discrimination performance was better for <sc> trials than <cs> trials regardless of session and modality condition. The differences in Weber fractions, not in bias, between intramodal and crossmodal condition was influenced by whether tactile or visual duration was used as the standard.

Our key finding is that the bias in duration estimation was stronger for longer durations than for shorter durations within both shorter and longer sessions. Furthermore, the bias was dependent on the relative duration within each session instead of its absolute duration. That is, the estimation of 1,000 ms was biased when it was the longer standard duration within the shorter sessions (with 700 ms and 1,000 ms being the two standard durations), whereas it was not biased when it was the shorter standard duration within the longer sessions (with 1,000 ms and 1,500 ms being the two standard durations). Our results are consistent with the findings of Jazayeri and Shadlen (2010), who found that the reproduction bias of sample temporal intervals was stronger for intervals from longer duration distributions (priors), and within each duration distributions, bias was stronger for the longer sample intervals. They suggested that this is because the longer intervals involve more uncertainty, and more uncertain judgments may rely more on the priors. With a “Ready-Set-Go” reproduction task, they postulated that the Bayesian temporal contextual modulation may be achieved by sensorimotor structures related to time reproduction (Schultz and Romo, 1992; Shadlen and Newsome, 2001; Gold et al., 2008). Importantly, we used a 2IFC task that measures perceptual rather than motor timing (see Lewis and Miall, 2003 for review).

We also found that longer standard durations were overestimated in <cs> trials and underestimated in <sc> trials, regardless of session and modality condition. This opposite direction of bias for two presentation orders can be explained by the idea that the central tendency effect is applied differently to the two orders within the context of the Bayesian model mentioned above. Another important finding of our study is that in both crossmodal and intramodal conditions, duration discrimination was better for the <sc> trials than <cs> trials. This finding is consistent with the Type B effect (Rammsayer and Wittkowski, 1990; Grondin and McAuley, 2009; Ulrich and Vorberg, 2009; Ellinghaus et al., 2018). Both central tendency effect and Type B effect can be accommodated with a memory mixing explanation supported by the pacemaker-accumulator model (Creelman, 1962; Treisman, 1963; Gibbon et al., 1984). According to this model, the temporal processing of our brain consists of an internal clock, a memory stage, and a decision stage. The internal “clock” that counts the pulses emitted by the pacemaker and the subjective timing depends on the number of pulses accumulated (Treisman, 1963; Allan and Kristofferson, 1974; Gibbon et al., 1997; Rammsayer and Ulrich, 2001). The number of pulses defining the duration is then stored in working memory, which later forms a more solid long-term memory. The central tendency effect would be stronger for the duration presented first than for the duration presented second due to the longer time that it is held in memory. When the standard duration is presented first (in the case of <sc> trials), it should be more uncertain. Thus, the central tendency effect should be stronger for the standard duration than for the comparison duration, resulting in the PSE shifting in the direction of the central tendency. In contrast, if the comparison duration was presented first (in the case of <cs> trials), the central tendency effect should be stronger for the comparison duration than for the standard duration, and the PSE would be shifted away in the opposite direction to the central tendency effect. This pattern of results is precisely what we found.

In terms of the Type B effect, it has been suggested by the Internal Reference Model that the current stimulus is not compared to the physical duration of the first-presented stimulus, but rather to an internal reference that combines the first-presented stimulus with a record of the previous stimulus history (Dyjas et al., 2012). Specifically, in <sc> trials, the standard duration is mixed into memory, while in <cs> trials the more variable comparison duration was mixed into memory. Thus, a less stable interval reference in <cs> trials resulted in worse discrimination than in <sc> trials (Lapid et al., 2008; Dyjas et al., 2012; Bausenhart et al., 2016; Ellinghaus et al., 2018). According to the model, a constant weight of g was applied to stimulus history and a weight of 1-g was applied to the first duration of the current trial. The internal reference was then the weighted summation of stimulus history and the first duration of the current trial. Previous studies have found the Type B effect for both random and blocked order of <sc> and <cs> trials (Nachmias, 2006; Nahum et al., 2010). Dyjas et al. (2012) demonstrated that the Internal Reference Model can predict the Type B effect for both random and blocked order. Critically, our results suggested that the Type B effect generalizes not only across stimulus attributes but also across modalities (vision and touch).

How do we compare durations from different modalities? The Pacemaker Model suggests that memory representations of durations from different modalities are stored centrally and amodally by counting the accumulated number of pulses (Wearden et al., 1998; Penney et al., 2000; Rammsayer and Ulrich, 2005; Ulrich et al., 2006). The pacemaker model can explain phenomena such as modality differences in duration estimation. For example, in the case of visual and auditory duration comparisons, it is believed that auditory signals drive the pacemaker at a faster rate than do visual signals (Wearden et al., 1998; Penney et al., 2000; Ulrich et al., 2006). Therefore, when compared to the mixed memory distribution, auditory durations would be judged longer, and visual durations would be judged shorter. This memory mixing theory assumes a single duration distribution for different modalities and proposes a central timing mechanism for different modalities. However, other researchers have proposed the existence of distributed local duration processing mechanisms for different sensory modalities (Gamache and Grondin, 2010; Rattat and Picard, 2012; Takahashi and Watanabe, 2012). The existence of distributed timing mechanisms for different modalities is supported by findings of brain activities specific to different modalities (Jantzen et al., 2005; Bueti et al., 2008). Consistent with the latter hypothesis, it has been found that duration adaptation is modality-specific and there is no transfer of learning effects from auditory duration discrimination to visual duration discrimination (Lapid et al., 2009; Heron et al., 2012). For example, Heron et al. (2012) proposed duration-selective channels specific to modality.

Our results cannot differentiate these two models. On the one hand, the bias for duration estimation in both Experiment 1 and 2 was similarly influenced by presentation order and standard duration, but not session and modality. Crossmodal and intramodal conditions exhibited similar tendencies to exhibit the Type B effect. This is consistent with a common mechanism underlying duration discrimination within and across vision and touch. On the other hand, we found a larger bias of duration estimation for <cs> trials than <sc> trials, and better discrimination performance in the intramodal, compared to the crossmodal condition in Experiment 2, indicating the potential contribution of a modality-specific mechanism. A limitation of our study is that the mean duration was the same for different modalities, thus not allowing us to discriminate whether there is a centralized timing mechanism between the two modalities. Future studies using different prior distributions for different modalities may provide a better test for discriminating between these mechanisms.

## Data Availability Statement

The raw data supporting the conclusions of this article will be made available by the authors, without undue reservation.

## Ethics Statement

The studies involving human participants were reviewed and approved by Institutional Review Board at the University of Nevada, Reno. The patients/participants provided their written informed consent to participate in this study.

## Author Contributions

YG and FJ conceived the experiments. YG and KM carried out the experiments. YG and MR analyzed data. YG, MR, MW, and FJ wrote the manuscript. All authors contributed to the article and approved the submitted version.

## Conflict of Interest

The authors declare that the research was conducted in the absence of any commercial or financial relationships that could be construed as a potential conflict of interest.
