# Supplementary material for: Duration Comparisons for Vision and Touch Are Dependent on Presentation Order and Temporal Context
Source: Front Integr Neurosci. 2021 Jun 23;15:664264. doi: 10.3389/fnint.2021.664264 (PMC8261066; doi:10.3389/fnint.2021.664264)
Supplement: Supplementary file 1 [file Data_Sheet_1.docx]

**Supplementary**

**
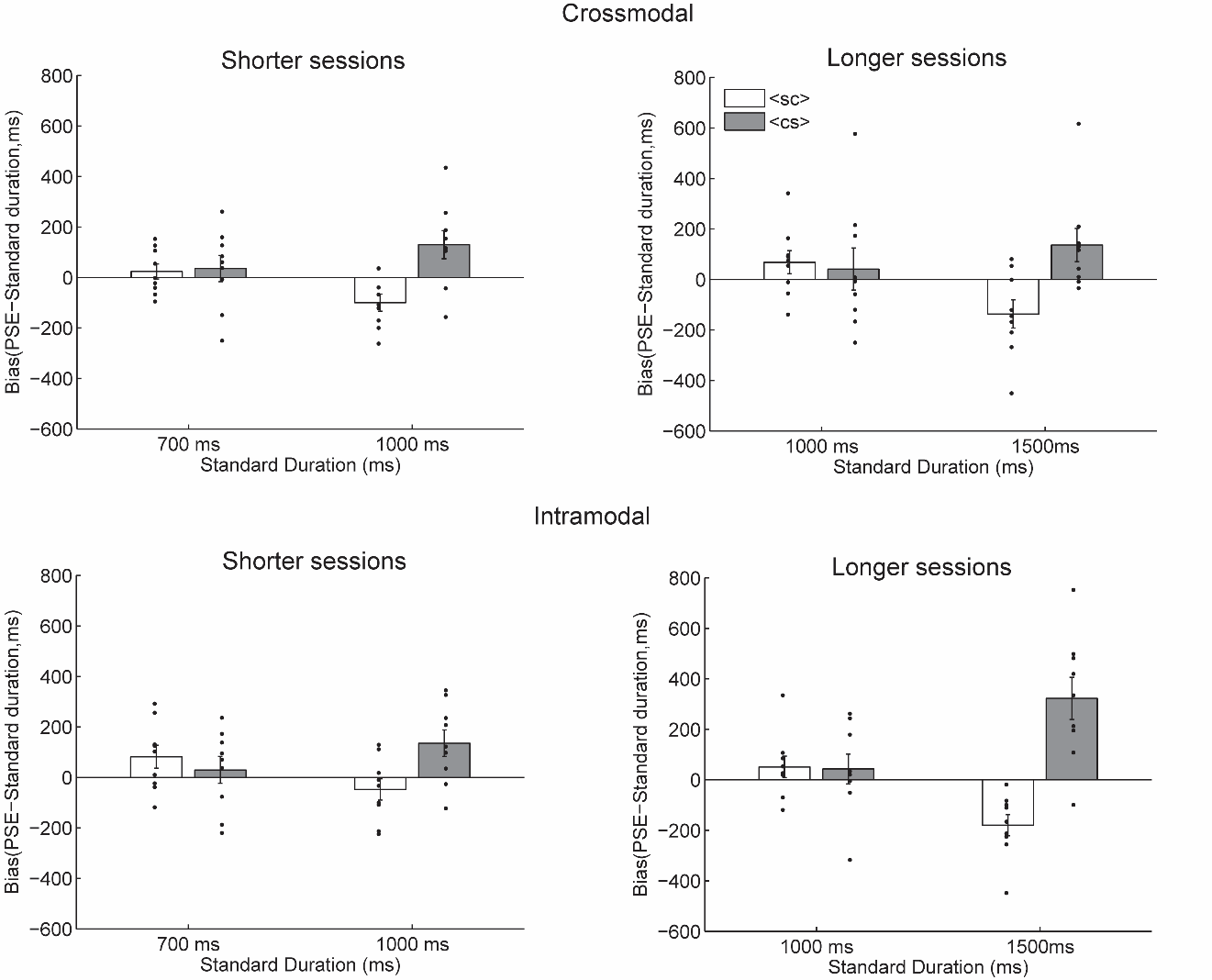
**

Figure S1. Individual data for bias (PSE- veridical standard duration) of Experiment 1. The bar plots showed mean bias in each combination of conditions and the dots showed individual data.

**
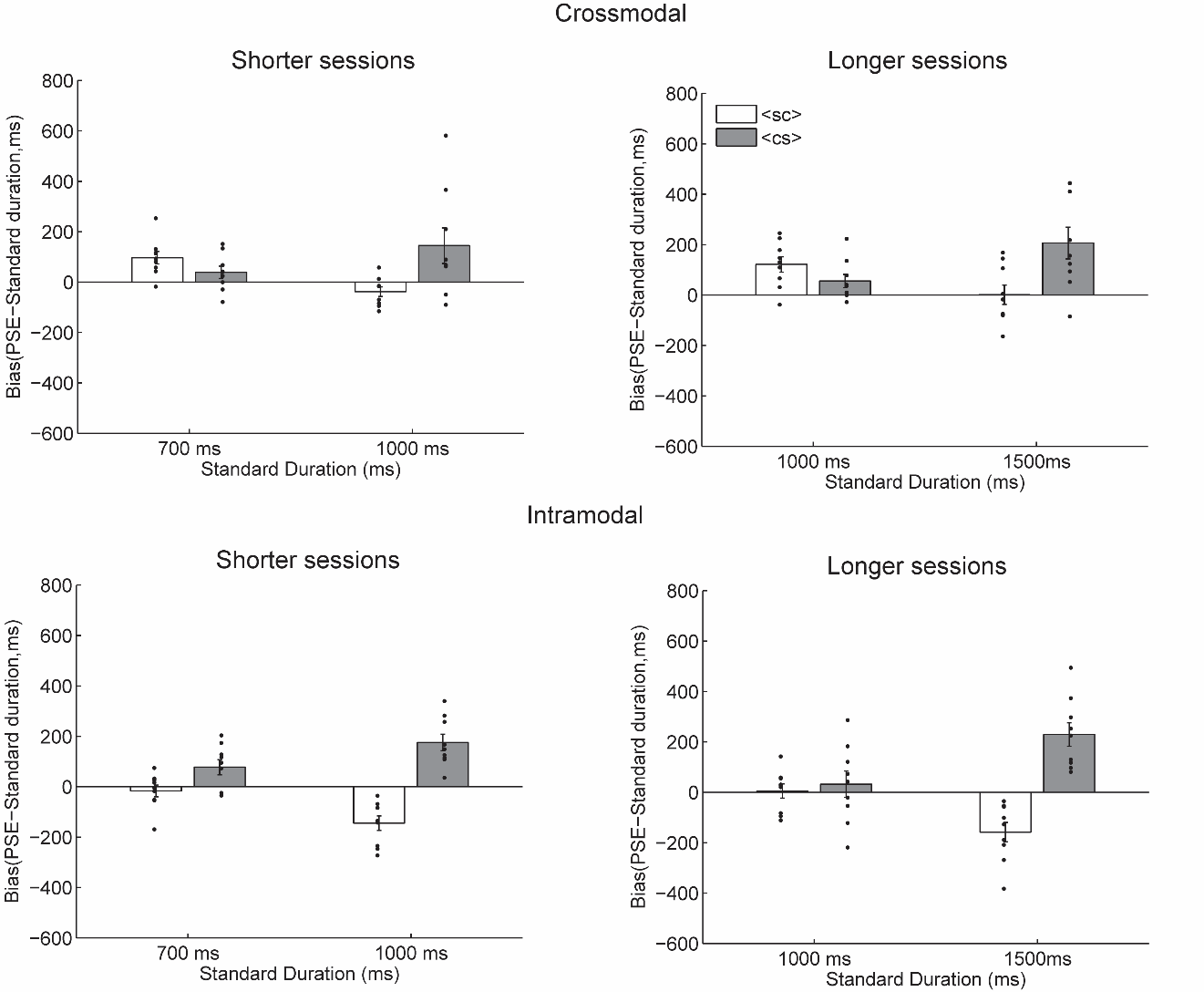
**

Figure S2. Individual data for bias (PSE- veridical standard duration) of Experiment 2. The bar plots showed mean bias in each combination of conditions and the dots showed individual data.

| \| **Model Comparison** \| \| \| \| \| \| \| \| \| \| \| \| \| \| \| \| \| --- \| --- \| --- \| --- \| --- \| --- \| --- \| --- \| --- \| --- \| --- \| --- \| --- \| --- \| --- \| --- \| \| **Models** \| **P(M)** \| \| \| \| **P(M\|data)** \| \| **BF _M_** \| \| \| \| **BF _10_** \| \| **error %** \| \| \| \| Null model (incl. subject) \|  \| 0.006 \|  \| \| 1.077e  -9 \|  \| \| 1.787e  -7 \| \|  \| 1.000 \|  \| \|  \|  \| \| \| presentation order + standard duration + presentation order  ✻  standard duration \|  \| 0.006 \|  \| \| 0.426 \|  \| \| 122.994 \| \|  \| 3.953e +8 \|  \| \| 3.113 \|  \| \| \| presentation order + standard duration + condition + presentation order  ✻  standard duration \|  \| 0.006 \|  \| \| 0.130 \|  \| \| 24.726 \| \|  \| 1.204e +8 \|  \| \| 2.531 \|  \| \| \| presentation order + session + standard duration + presentation order  ✻  standard duration \|  \| 0.006 \|  \| \| 0.076 \|  \| \| 13.722 \| \|  \| 7.091e +7 \|  \| \| 2.682 \|  \| \| \| presentation order + session + standard duration + presentation order  ✻  session + presentation order  ✻  standard duration \|  \| 0.006 \|  \| \| 0.066 \|  \| \| 11.706 \| \|  \| 6.118e +7 \|  \| \| 3.292 \|  \| \| \| presentation order + standard duration + condition + presentation order  ✻  standard duration + standard duration  ✻  condition \|  \| 0.006 \|  \| \| 0.045 \|  \| \| 7.815 \| \|  \| 4.176e +7 \|  \| \| 6.139 \|  \| \| \| presentation order + standard duration + condition + presentation order  ✻  standard duration + presentation order  ✻  condition \|  \| 0.006 \|  \| \| 0.035 \|  \| \| 6.087 \| \|  \| 3.285e +7 \|  \| \| 2.129 \|  \| \| \| presentation order + session + standard duration + presentation order  ✻  standard duration + session  ✻  standard duration \|  \| 0.006 \|  \| \| 0.024 \|  \| \| 4.158 \|  \| \| 2.270e +7 \|  \| \| 24.793 \|  \| \| \| presentation order + session + standard duration + condition + presentation order  ✻  standard duration \|  \| 0.006 \|  \| \| 0.023 \|  \| \| 3.951 \|  \| \| 2.159e +7 \|  \| \| 2.991 \|  \| \| \| presentation order + session + standard duration + condition + presentation order  ✻  session + presentation order  ✻  standard duration \|  \| 0.006 \|  \| \| 0.021 \|  \| \| 3.573 \|  \| \| 1.957e +7 \|  \| \| 4.379 \|  \| \| \| presentation order + session + standard duration + presentation order  ✻  session + presentation order  ✻  standard duration + session  ✻  standard duration + presentation order  ✻  session  ✻  standard duration \|  \| 0.006 \|  \| \| 0.018 \|  \| \| 3.116 \|  \| \| 1.711e +7 \|  \| \| 9.830 \|  \| \| \| presentation order + session + standard duration + presentation order  ✻  session + presentation order  ✻  standard duration + session  ✻  standard duration \|  \| 0.006 \|  \| \| 0.016 \|  \| \| 2.758 \|  \| \| 1.518e +7 \|  \| \| 4.340 \|  \| \| \| presentation order + standard duration + condition + presentation order  ✻  standard duration + presentation order  ✻  condition + standard duration  ✻  condition \|  \| 0.006 \|  \| \| 0.012 \|  \| \| 2.067 \|  \| \| 1.142e +7 \|  \| \| 3.736 \|  \| \| \| presentation order + session + standard duration + condition + presentation order  ✻  standard duration + standard duration  ✻  condition \|  \| 0.006 \|  \| \| 0.009 \|  \| \| 1.567 \|  \| \| 8.685e +6 \|  \| \| 18.968 \|  \| \| \| presentation order + session + standard duration + condition + presentation order  ✻  standard duration + presentation order  ✻  condition \|  \| 0.006 \|  \| \| 0.009 \|  \| \| 1.450 \|  \| \| 8.043e +6 \|  \| \| 24.478 \|  \| \| \| presentation order + session + standard duration + condition + presentation order  ✻  session + presentation order  ✻  standard duration + session  ✻  standard duration + presentation order  ✻  session  ✻  standard duration \|  \| 0.006 \|  \| \| 0.008 \|  \| \| 1.271 \|  \| \| 7.058e +6 \|  \| \| 31.282 \|  \| \| \| presentation order + session + standard duration + condition + presentation order  ✻  session + presentation order  ✻  standard duration + standard duration  ✻  condition \|  \| 0.006 \|  \| \| 0.007 \|  \| \| 1.148 \|  \| \| 6.376e +6 \|  \| \| 6.405 \|  \| \| \| presentation order + session + standard duration + condition + presentation order  ✻  standard duration + session  ✻  standard duration \|  \| 0.006 \|  \| \| 0.007 \|  \| \| 1.109 \|  \| \| 6.161e +6 \|  \| \| 9.885 \|  \| \| \| presentation order + session + standard duration + condition + presentation order  ✻  session + presentation order  ✻  standard duration + presentation order  ✻  condition \|  \| 0.006 \|  \| \| 0.006 \|  \| \| 0.992 \|  \| \| 5.514e +6 \|  \| \| 4.158 \|  \| \| \| presentation order + standard duration + condition + presentation order  ✻  standard duration + presentation order  ✻  condition + standard duration  ✻  condition + presentation order  ✻  standard duration  ✻  condition \|  \| 0.006 \|  \| \| 0.006 \|  \| \| 0.933 \|  \| \| 5.190e +6 \|  \| \| 4.146 \|  \| \| \| presentation order + session + standard duration + condition + presentation order  ✻  session + presentation order  ✻  standard duration + session  ✻  condition \|  \| 0.006 \|  \| \| 0.005 \|  \| \| 0.898 \|  \| \| 4.996e +6 \|  \| \| 5.322 \|  \| \| \| presentation order + session + standard duration + condition + presentation order  ✻  standard duration + session  ✻  condition \|  \| 0.006 \|  \| \| 0.005 \|  \| \| 0.887 \|  \| \| 4.936e +6 \|  \| \| 3.027 \|  \| \| \| presentation order + session + standard duration + condition + presentation order  ✻  session + presentation order  ✻  standard duration + session  ✻  standard duration \|  \| 0.006 \|  \| \| 0.005 \|  \| \| 0.785 \|  \| \| 4.370e +6 \|  \| \| 2.704 \|  \| \| \| presentation order + session + standard duration + condition + presentation order  ✻  session + presentation order  ✻  standard duration + session  ✻  standard duration + standard duration  ✻  condition + presentation order  ✻  session  ✻  standard duration \|  \| 0.006 \| \|  \| 0.004 \|  \| \| 0.589 \|  \| \| 3.282e +6 \|  \| \| 50.565 \|  \| \| \| presentation order + session + standard duration + condition + presentation order  ✻  session + presentation order  ✻  standard duration + presentation order  ✻  condition + standard duration  ✻  condition \|  \| 0.006 \| \|  \| 0.003 \|  \| \| 0.423 \|  \| \| 2.362e +6 \|  \| \| 17.394 \|  \| \| \| presentation order + session + standard duration + condition + presentation order  ✻  standard duration + presentation order  ✻  condition + standard duration  ✻  condition \|  \| 0.006 \| \|  \| 0.002 \|  \| \| 0.372 \|  \| \| 2.079e +6 \|  \| \| 9.483 \|  \| \| \| presentation order + session + standard duration + condition + presentation order  ✻  standard duration + session  ✻  standard duration + presentation order  ✻  condition \|  \| 0.006 \| \|  \| 0.002 \|  \| \| 0.328 \|  \| \| 1.834e +6 \|  \| \| 17.883 \|  \| \| \| presentation order + session + standard duration + condition + presentation order  ✻  standard duration + session  ✻  condition + standard duration  ✻  condition \|  \| 0.006 \| \|  \| 0.002 \|  \| \| 0.284 \|  \| \| 1.584e +6 \|  \| \| 3.042 \|  \| \| \| presentation order + session + standard duration + condition + presentation order  ✻  session + presentation order  ✻  standard duration + session  ✻  standard duration + standard duration  ✻  condition \|  \| 0.006 \| \|  \| 0.002 \|  \| \| 0.281 \|  \| \| 1.568e +6 \|  \| \| 6.660 \|  \| \| \| presentation order + session + standard duration + condition + presentation order  ✻  session + presentation order  ✻  standard duration + session  ✻  condition + standard duration  ✻  condition \|  \| 0.006 \| \|  \| 0.002 \|  \| \| 0.277 \|  \| \| 1.547e +6 \|  \| \| 6.403 \|  \| \| \| presentation order + session + standard duration + condition + presentation order  ✻  standard duration + session  ✻  standard duration + standard duration  ✻  condition \|  \| 0.006 \| \|  \| 0.002 \|  \| \| 0.276 \|  \| \| 1.540e +6 \|  \| \| 3.370 \|  \| \| \| presentation order + session + standard duration + condition + presentation order  ✻  standard duration + presentation order  ✻  condition + session  ✻  condition \|  \| 0.006 \| \|  \| 0.002 \|  \| \| 0.271 \|  \| \| 1.515e +6 \|  \| \| 7.828 \|  \| \| \| presentation order + session + standard duration + condition + presentation order  ✻  session + presentation order  ✻  standard duration + presentation order  ✻  condition + session  ✻  condition \|  \| 0.006 \| \|  \| 0.001 \|  \| \| 0.249 \|  \| \| 1.391e +6 \|  \| \| 4.508 \|  \| \| \| presentation order + session + standard duration + condition + presentation order  ✻  session + presentation order  ✻  standard duration + session  ✻  standard duration + presentation order  ✻  condition + presentation order  ✻  session  ✻  standard duration \|  \| 0.006 \| \|  \| 0.001 \|  \| \| 0.239 \|  \| \| 1.334e +6 \|  \| \| 4.190 \|  \| \| \| presentation order + session + standard duration + condition + presentation order  ✻  session + presentation order  ✻  standard duration + presentation order  ✻  condition + session  ✻  condition + presentation order  ✻  session  ✻  condition \|  \| 0.006 \| \|  \| 0.001 \|  \| \| 0.237 \|  \| \| 1.323e +6 \|  \| \| 8.074 \|  \| \| \| presentation order + session + standard duration + condition + presentation order  ✻  session + presentation order  ✻  standard duration + session  ✻  standard duration + presentation order  ✻  condition \|  \| 0.006 \| \|  \| 0.001 \|  \| \| 0.232 \|  \| \| 1.294e +6 \|  \| \| 3.538 \|  \| \| \| presentation order + session + standard duration + condition + presentation order  ✻  session + presentation order  ✻  standard duration + session  ✻  standard duration + session  ✻  condition \|  \| 0.006 \|  \| \| 0.001 \|  \| \| 0.228 \|  \| \| 1.273e +6 \|  \| \| 9.675 \|  \| \| \| presentation order + session + standard duration + condition + presentation order  ✻  session + presentation order  ✻  standard duration + session  ✻  standard duration + session  ✻  condition + presentation order  ✻  session  ✻  standard duration \|  \| 0.006 \|  \| \| 0.001 \|  \| \| 0.226 \|  \| \| 1.265e +6 \|  \| \| 7.671 \|  \| \| \| presentation order + session + standard duration + condition + presentation order  ✻  standard duration + session  ✻  standard duration + session  ✻  condition \|  \| 0.006 \|  \| \| 0.001 \|  \| \| 0.222 \|  \| \| 1.240e +6 \|  \| \| 4.562 \|  \| \| \| presentation order + session + standard duration + condition + presentation order  ✻  session + presentation order  ✻  standard duration + presentation order  ✻  condition + standard duration  ✻  condition + presentation order  ✻  standard duration  ✻  condition \|  \| 0.006 \|  \| \| 0.001 \|  \| \| 0.173 \|  \| \| 968081.401 \|  \| \| 7.638 \|  \| \| \| presentation order + session + standard duration + condition + presentation order  ✻  standard duration + presentation order  ✻  condition + standard duration  ✻  condition + presentation order  ✻  standard duration  ✻  condition \|  \| 0.006 \|  \| \| 0.001 \|  \| \| 0.170 \|  \| \| 950956.759 \|  \| \| 4.206 \|  \| \| \| presentation order + session + standard duration + condition + presentation order  ✻  session + presentation order  ✻  standard duration + presentation order  ✻  condition + session  ✻  condition + standard duration  ✻  condition + presentation order  ✻  session  ✻  condition \|  \| 0.006 \|  \| \| 5.927e  -4 \|  \| \| 0.098 \|  \| \| 550403.332 \|  \| \| 13.661 \|  \| \| \| presentation order + session + standard duration + condition + presentation order  ✻  session + presentation order  ✻  standard duration + session  ✻  standard duration + presentation order  ✻  condition + standard duration  ✻  condition + presentation order  ✻  session  ✻  standard duration \|  \| 0.006 \|  \| \| 5.127e  -4 \|  \| \| 0.085 \|  \| \| 476155.389 \|  \| \| 15.158 \|  \| \| \| presentation order + session + standard duration + condition + presentation order  ✻  standard duration + session  ✻  standard duration + presentation order  ✻  condition + standard duration  ✻  condition \|  \| 0.006 \|  \| \| 5.072e  -4 \|  \| \| 0.084 \|  \| \| 471034.730 \|  \| \| 5.299 \|  \| \| \| presentation order + session + standard duration + condition + presentation order  ✻  standard duration + presentation order  ✻  condition + session  ✻  condition + standard duration  ✻  condition \|  \| 0.006 \|  \| \| 4.944e  -4 \|  \| \| 0.082 \|  \| \| 459127.490 \|  \| \| 3.915 \|  \| \| \| presentation order + session + standard duration + condition + presentation order  ✻  session + presentation order  ✻  standard duration + session  ✻  standard duration + presentation order  ✻  condition + standard duration  ✻  condition \|  \| 0.006 \|  \| \| 4.697e  -4 \|  \| \| 0.078 \|  \| \| 436178.638 \|  \| \| 6.844 \|  \| \| \| presentation order + session + standard duration + condition + presentation order  ✻  standard duration + session  ✻  standard duration + session  ✻  condition + standard duration  ✻  condition \|  \| 0.006 \|  \| \| 4.483e  -4 \|  \| \| 0.074 \|  \| \| 416336.113 \|  \| \| 6.406 \|  \| \| \| presentation order + session + standard duration + condition + presentation order  ✻  session + presentation order  ✻  standard duration + presentation order  ✻  condition + session  ✻  condition + standard duration  ✻  condition \|  \| 0.006 \|  \| \| 4.405e  -4 \|  \| \| 0.073 \|  \| \| 409128.444 \|  \| \| 3.258 \|  \| \| \| presentation order + session + standard duration + condition + presentation order  ✻  session + presentation order  ✻  standard duration + session  ✻  standard duration + session  ✻  condition + standard duration  ✻  condition + presentation order  ✻  session  ✻  standard duration \|  \| 0.006 \|  \| \| 4.153e  -4 \|  \| \| 0.069 \|  \| \| 385733.145 \|  \| \| 8.518 \|  \| \| \| presentation order + session + standard duration + condition + presentation order  ✻  session + presentation order  ✻  standard duration + session  ✻  standard duration + presentation order  ✻  condition + session  ✻  condition + presentation order  ✻  session  ✻  standard duration + presentation order  ✻  session  ✻  condition \|  \| 0.006 \|  \| \| 4.110e  -4 \|  \| \| 0.068 \|  \| \| 381708.330 \|  \| \| 11.315 \|  \| \| \| presentation order + session + standard duration + condition + presentation order  ✻  session + presentation order  ✻  standard duration + session  ✻  standard duration + presentation order  ✻  condition + session  ✻  condition + presentation order  ✻  session  ✻  condition \|  \| 0.006 \|  \| \| 4.028e  -4 \|  \| \| 0.067 \|  \| \| 374088.249 \|  \| \| 6.340 \|  \| \| \| presentation order + session + standard duration + condition + presentation order  ✻  standard duration + session  ✻  standard duration + presentation order  ✻  condition + session  ✻  condition \|  \| 0.006 \|  \| \| 3.971e  -4 \|  \| \| 0.066 \|  \| \| 368802.165 \|  \| \| 4.857 \|  \| \| \| presentation order + session + standard duration + condition + presentation order  ✻  session + presentation order  ✻  standard duration + session  ✻  standard duration + presentation order  ✻  condition + session  ✻  condition \|  \| 0.006 \|  \| \| 3.808e  -4 \|  \| \| 0.063 \|  \| \| 353627.964 \|  \| \| 7.793 \|  \| \| \| presentation order + session + standard duration + condition + presentation order  ✻  session + presentation order  ✻  standard duration + session  ✻  standard duration + session  ✻  condition + standard duration  ✻  condition \|  \| 0.006 \|  \| \| 3.543e  -4 \|  \| \| 0.059 \|  \| \| 329034.702 \|  \| \| 4.471 \|  \| \| \| presentation order + session + standard duration + condition + presentation order  ✻  session + presentation order  ✻  standard duration + session  ✻  standard duration + presentation order  ✻  condition + session  ✻  condition + presentation order  ✻  session  ✻  standard duration \|  \| 0.006 \|  \| \| 3.448e  -4 \|  \| \| 0.057 \|  \| \| 320217.535 \|  \| \| 4.641 \|  \| \| \| presentation order + session + standard duration + condition + presentation order  ✻  standard duration + session  ✻  standard duration + presentation order  ✻  condition + standard duration  ✻  condition + presentation order  ✻  standard duration  ✻  condition \|  \| 0.006 \|  \| \| 2.795e  -4 \|  \| \| 0.046 \|  \| \| 259594.918 \|  \| \| 9.285 \|  \| \| \| presentation order + session + standard duration + condition + presentation order  ✻  session + presentation order  ✻  standard duration + session  ✻  standard duration + presentation order  ✻  condition + standard duration  ✻  condition + presentation order  ✻  session  ✻  standard duration + presentation order  ✻  standard duration  ✻  condition \|  \| 0.006 \|  \| \| 2.717e  -4 \|  \| \| 0.045 \|  \| \| 252286.712 \|  \| \| 8.373 \|  \| \| \| presentation order + session + standard duration + condition + presentation order  ✻  session + presentation order  ✻  standard duration + session  ✻  standard duration + presentation order  ✻  condition + standard duration  ✻  condition + presentation order  ✻  standard duration  ✻  condition \|  \| 0.006 \|  \| \| 2.574e  -4 \|  \| \| 0.043 \|  \| \| 239064.516 \|  \| \| 7.938 \|  \| \| \| presentation order + session + standard duration + condition + presentation order  ✻  standard duration + presentation order  ✻  condition + session  ✻  condition + standard duration  ✻  condition + presentation order  ✻  standard duration  ✻  condition \|  \| 0.006 \|  \| \| 2.503e  -4 \|  \| \| 0.042 \|  \| \| 232476.814 \|  \| \| 5.738 \|  \| \| \| presentation order + session + standard duration + condition + presentation order  ✻  standard duration + session  ✻  standard duration + session  ✻  condition + standard duration  ✻  condition + session  ✻  standard duration  ✻  condition \|  \| 0.006 \|  \| \| 2.274e  -4 \|  \| \| 0.038 \|  \| \| 211173.235 \|  \| \| 19.153 \|  \| \| \| presentation order + session + standard duration + condition + presentation order  ✻  session + presentation order  ✻  standard duration + presentation order  ✻  condition + session  ✻  condition + standard duration  ✻  condition + presentation order  ✻  standard duration  ✻  condition \|  \| 0.006 \|  \| \| 2.272e  -4 \|  \| \| 0.038 \|  \| \| 210964.879 \|  \| \| 5.474 \|  \| \| \| presentation order + session + standard duration + condition + presentation order  ✻  session + presentation order  ✻  standard duration + presentation order  ✻  condition + session  ✻  condition + standard duration  ✻  condition + presentation order  ✻  session  ✻  condition + presentation order  ✻  standard duration  ✻  condition \|  \| 0.006 \|  \| \| 2.265e  -4 \|  \| \| 0.038 \|  \| \| 210377.076 \|  \| \| 7.606 \|  \| \| \| presentation order + session + standard duration + condition + presentation order  ✻  session + presentation order  ✻  standard duration + session  ✻  standard duration + presentation order  ✻  condition + session  ✻  condition + standard duration  ✻  condition + presentation order  ✻  session  ✻  standard duration + presentation order  ✻  session  ✻  condition \|  \| 0.006 \|  \| \| 1.972e  -4 \|  \| \| 0.033 \|  \| \| 183155.815 \|  \| \| 38.276 \|  \| \| \| presentation order + session + standard duration + condition + presentation order  ✻  session + presentation order  ✻  standard duration + session  ✻  standard duration + presentation order  ✻  condition + session  ✻  condition + standard duration  ✻  condition + presentation order  ✻  session  ✻  condition \|  \| 0.006 \|  \| \| 1.783e  -4 \|  \| \| 0.030 \|  \| \| 165581.806 \|  \| \| 22.894 \|  \| \| \| presentation order + session + standard duration + condition + presentation order  ✻  session + presentation order  ✻  standard duration + session  ✻  standard duration + session  ✻  condition + standard duration  ✻  condition + session  ✻  standard duration  ✻  condition \|  \| 0.006 \|  \| \| 1.726e  -4 \|  \| \| 0.029 \|  \| \| 160339.846 \|  \| \| 14.539 \|  \| \| \| presentation order + session + standard duration + condition + presentation order  ✻  session + presentation order  ✻  standard duration + session  ✻  standard duration + session  ✻  condition + standard duration  ✻  condition + presentation order  ✻  session  ✻  standard duration + session  ✻  standard duration  ✻  condition \|  \| 0.006 \|  \| \| 1.655e  -4 \|  \| \| 0.027 \|  \| \| 153662.842 \|  \| \| 12.938 \|  \| \| \| presentation order + session + standard duration + condition + presentation order  ✻  standard duration + session  ✻  standard duration + presentation order  ✻  condition + session  ✻  condition + standard duration  ✻  condition \|  \| 0.006 \|  \| \| 1.420e  -4 \|  \| \| 0.024 \|  \| \| 131833.499 \|  \| \| 14.639 \|  \| \| \| presentation order + session + standard duration + condition + presentation order  ✻  session + presentation order  ✻  standard duration + session  ✻  standard duration + presentation order  ✻  condition + session  ✻  condition + standard duration  ✻  condition \|  \| 0.006 \|  \| \| 1.158e  -4 \|  \| \| 0.019 \|  \| \| 107527.247 \|  \| \| 8.375 \|  \| \| \| presentation order + session + standard duration + condition + presentation order  ✻  session + presentation order  ✻  standard duration + session  ✻  standard duration + presentation order  ✻  condition + session  ✻  condition + standard duration  ✻  condition + presentation order  ✻  session  ✻  standard duration \|  \| 0.006 \|  \| \| 1.146e  -4 \|  \| \| 0.019 \|  \| \| 106416.148 \|  \| \| 6.238 \|  \| \| \| presentation order + session + standard duration + condition + presentation order  ✻  session + presentation order  ✻  standard duration + session  ✻  standard duration + presentation order  ✻  condition + session  ✻  condition + standard duration  ✻  condition + presentation order  ✻  session  ✻  standard duration + presentation order  ✻  standard duration  ✻  condition \|  \| 0.006 \|  \| \| 7.680e  -5 \|  \| \| 0.013 \|  \| \| 71322.147 \|  \| \| 14.219 \|  \| \| \| presentation order + session + standard duration + condition + presentation order  ✻  session + presentation order  ✻  standard duration + session  ✻  standard duration + presentation order  ✻  condition + session  ✻  condition + standard duration  ✻  condition + presentation order  ✻  session  ✻  standard duration + presentation order  ✻  session  ✻  condition + presentation order  ✻  standard duration  ✻  condition \|  \| 0.006 \|  \| \| 7.374e  -5 \|  \| \| 0.012 \|  \| \| 68482.855 \|  \| \| 15.213 \|  \| \| \| presentation order + session + standard duration + condition + presentation order  ✻  session + presentation order  ✻  standard duration + session  ✻  standard duration + presentation order  ✻  condition + session  ✻  condition + standard duration  ✻  condition + presentation order  ✻  session  ✻  standard duration + presentation order  ✻  session  ✻  condition + session  ✻  standard duration  ✻  condition \|  \| 0.006 \|  \| \| 6.918e  -5 \|  \| \| 0.011 \|  \| \| 64244.679 \|  \| \| 36.807 \|  \| \| \| presentation order + session + standard duration + condition + presentation order  ✻  session + presentation order  ✻  standard duration + session  ✻  standard duration + presentation order  ✻  condition + session  ✻  condition + standard duration  ✻  condition + presentation order  ✻  session  ✻  condition + presentation order  ✻  standard duration  ✻  condition \|  \| 0.006 \|  \| \| 6.899e  -5 \|  \| \| 0.011 \|  \| \| 64070.162 \|  \| \| 11.242 \|  \| \| \| presentation order + session + standard duration + condition + presentation order  ✻  standard duration + session  ✻  standard duration + presentation order  ✻  condition + session  ✻  condition + standard duration  ✻  condition + presentation order  ✻  standard duration  ✻  condition \|  \| 0.006 \|  \| \| 6.767e  -5 \|  \| \| 0.011 \|  \| \| 62844.984 \|  \| \| 8.402 \|  \| \| \| presentation order + session + standard duration + condition + presentation order  ✻  session + presentation order  ✻  standard duration + session  ✻  standard duration + presentation order  ✻  condition + session  ✻  condition + standard duration  ✻  condition + presentation order  ✻  standard duration  ✻  condition \|  \| 0.006 \|  \| \| 6.425e  -5 \|  \| \| 0.011 \|  \| \| 59668.573 \|  \| \| 18.305 \|  \| \| \| presentation order + session + standard duration + condition + presentation order  ✻  session + presentation order  ✻  standard duration + session  ✻  standard duration + presentation order  ✻  condition + session  ✻  condition + standard duration  ✻  condition + presentation order  ✻  standard duration  ✻  condition + session  ✻  standard duration  ✻  condition \|  \| 0.006 \|  \| \| 5.971e  -5 \|  \| \| 0.010 \|  \| \| 55452.910 \|  \| \| 44.305 \|  \| \| \| presentation order + session + standard duration + condition + presentation order  ✻  session + presentation order  ✻  standard duration + session  ✻  standard duration + presentation order  ✻  condition + session  ✻  condition + standard duration  ✻  condition + presentation order  ✻  session  ✻  condition + session  ✻  standard duration  ✻  condition \|  \| 0.006 \|  \| \| 5.159e  -5 \|  \| \| 0.009 \|  \| \| 47910.338 \|  \| \| 10.375 \|  \| \| \| presentation order + session + standard duration + condition + presentation order  ✻  session + presentation order  ✻  standard duration + session  ✻  standard duration + presentation order  ✻  condition + session  ✻  condition + standard duration  ✻  condition + presentation order  ✻  session  ✻  standard duration + session  ✻  standard duration  ✻  condition \|  \| 0.006 \|  \| \| 4.903e  -5 \|  \| \| 0.008 \|  \| \| 45535.252 \|  \| \| 10.043 \|  \| \| \| presentation order + session + standard duration + condition + presentation order  ✻  standard duration + session  ✻  standard duration + presentation order  ✻  condition + session  ✻  condition + standard duration  ✻  condition + session  ✻  standard duration  ✻  condition \|  \| 0.006 \|  \| \| 4.759e  -5 \|  \| \| 0.008 \|  \| \| 44196.008 \|  \| \| 7.857 \|  \| \| \| presentation order + session + standard duration + condition + presentation order  ✻  session + presentation order  ✻  standard duration + session  ✻  standard duration + presentation order  ✻  condition + session  ✻  condition + standard duration  ✻  condition + session  ✻  standard duration  ✻  condition \|  \| 0.006 \|  \| \| 4.448e  -5 \|  \| \| 0.007 \|  \| \| 41311.827 \|  \| \| 5.816 \|  \| \| \| presentation order + session + standard duration + condition + presentation order  ✻  session + presentation order  ✻  standard duration + session  ✻  standard duration + presentation order  ✻  condition + session  ✻  condition + standard duration  ✻  condition + presentation order  ✻  session  ✻  standard duration + presentation order  ✻  session  ✻  condition + presentation order  ✻  standard duration  ✻  condition + session  ✻  standard duration  ✻  condition \|  \| 0.006 \|  \| \| 3.151e  -5 \|  \| \| 0.005 \|  \| \| 29262.390 \|  \| \| 19.703 \|  \| \| \| presentation order + session + standard duration + condition + presentation order  ✻  standard duration + session  ✻  standard duration + presentation order  ✻  condition + session  ✻  condition + standard duration  ✻  condition + presentation order  ✻  standard duration  ✻  condition + session  ✻  standard duration  ✻  condition \|  \| 0.006 \|  \| \| 2.605e  -5 \|  \| \| 0.004 \|  \| \| 24190.314 \|  \| \| 10.267 \|  \| \| \| presentation order + session + standard duration + condition + presentation order  ✻  session + presentation order  ✻  standard duration + session  ✻  standard duration + presentation order  ✻  condition + session  ✻  condition + standard duration  ✻  condition + presentation order  ✻  session  ✻  condition + presentation order  ✻  standard duration  ✻  condition + session  ✻  standard duration  ✻  condition \|  \| 0.006 \|  \| \| 2.506e  -5 \|  \| \| 0.004 \|  \| \| 23275.778 \|  \| \| 18.363 \|  \| \| \| presentation order + session + standard duration + condition + presentation order  ✻  session + presentation order  ✻  standard duration + session  ✻  standard duration + presentation order  ✻  condition + session  ✻  condition + standard duration  ✻  condition + presentation order  ✻  session  ✻  standard duration + presentation order  ✻  standard duration  ✻  condition + session  ✻  standard duration  ✻  condition \|  \| 0.006 \|  \| \| 2.329e  -5 \|  \| \| 0.004 \|  \| \| 21626.874 \|  \| \| 6.307 \|  \| \| \| presentation order + condition + presentation order  ✻  condition \|  \| 0.006 \|  \| \| 2.177e  -5 \|  \| \| 0.004 \|  \| \| 20222.128 \|  \| \| 98.824 \|  \| \| \| presentation order + session + standard duration + condition + presentation order  ✻  session + presentation order  ✻  standard duration + session  ✻  standard duration + presentation order  ✻  condition + session  ✻  condition + standard duration  ✻  condition + presentation order  ✻  session  ✻  standard duration + presentation order  ✻  session  ✻  condition + presentation order  ✻  standard duration  ✻  condition + session  ✻  standard duration  ✻  condition + presentation order  ✻  session  ✻  standard duration  ✻  condition \|  \| 0.006 \|  \| \| 1.448e  -5 \|  \| \| 0.002 \|  \| \| 13444.686 \|  \| \| 11.771 \|  \| \| \| presentation order \|  \| 0.006 \|  \| \| 3.385e  -6 \|  \| \| 5.619e  -4 \|  \| \| 3143.373 \|  \| \| 1.249 \|  \| \| \| presentation order + condition \|  \| 0.006 \|  \| \| 9.351e  -7 \|  \| \| 1.552e  -4 \|  \| \| 868.455 \|  \| \| 1.670 \|  \| \| \| presentation order + session \|  \| 0.006 \|  \| \| 6.868e  -7 \|  \| \| 1.140e  -4 \|  \| \| 637.882 \|  \| \| 14.020 \|  \| \| \| presentation order + standard duration \|  \| 0.006 \|  \| \| 6.435e  -7 \|  \| \| 1.068e  -4 \|  \| \| 597.671 \|  \| \| 1.289 \|  \| \| \| presentation order + session + presentation order  ✻  session \|  \| 0.006 \|  \| \| 4.072e  -7 \|  \| \| 6.759e  -5 \|  \| \| 378.158 \|  \| \| 2.564 \|  \| \| \| presentation order + session + condition \|  \| 0.006 \|  \| \| 1.837e  -7 \|  \| \| 3.050e  -5 \|  \| \| 170.616 \|  \| \| 5.604 \|  \| \| \| presentation order + standard duration + condition \|  \| 0.006 \|  \| \| 1.833e  -7 \|  \| \| 3.043e  -5 \|  \| \| 170.227 \|  \| \| 1.838 \|  \| \| \| presentation order + session + condition + presentation order  ✻  session \|  \| 0.006 \|  \| \| 1.207e  -7 \|  \| \| 2.003e  -5 \|  \| \| 112.076 \|  \| \| 6.697 \|  \| \| \| presentation order + session + standard duration \|  \| 0.006 \|  \| \| 1.162e  -7 \|  \| \| 1.929e  -5 \|  \| \| 107.941 \|  \| \| 2.219 \|  \| \| \| presentation order + session + standard duration + presentation order  ✻  session \|  \| 0.006 \|  \| \| 8.141e  -8 \|  \| \| 1.351e  -5 \|  \| \| 75.605 \|  \| \| 2.464 \|  \| \| \| presentation order + standard duration + condition + standard duration  ✻  condition \|  \| 0.006 \|  \| \| 5.369e  -8 \|  \| \| 8.913e  -6 \|  \| \| 49.864 \|  \| \| 2.468 \|  \| \| \| presentation order + session + condition + presentation order  ✻  session + session  ✻  condition \|  \| 0.006 \|  \| \| 5.280e  -8 \|  \| \| 8.765e  -6 \|  \| \| 49.039 \|  \| \| 36.410 \|  \| \| \| presentation order + standard duration + condition + presentation order  ✻  condition \|  \| 0.006 \|  \| \| 5.192e  -8 \|  \| \| 8.618e  -6 \|  \| \| 48.216 \|  \| \| 3.523 \|  \| \| \| presentation order + session + condition + presentation order  ✻  condition \|  \| 0.006 \|  \| \| 4.442e  -8 \|  \| \| 7.373e  -6 \|  \| \| 41.250 \|  \| \| 1.944 \|  \| \| \| presentation order + session + condition + session  ✻  condition \|  \| 0.006 \|  \| \| 3.846e  -8 \|  \| \| 6.384e  -6 \|  \| \| 35.717 \|  \| \| 2.331 \|  \| \| \| presentation order + session + standard duration + condition \|  \| 0.006 \|  \| \| 3.605e  -8 \|  \| \| 5.984e  -6 \|  \| \| 33.478 \|  \| \| 5.076 \|  \| \| \| presentation order + session + condition + presentation order  ✻  session + presentation order  ✻  condition \|  \| 0.006 \|  \| \| 3.261e  -8 \|  \| \| 5.413e  -6 \|  \| \| 30.283 \|  \| \| 2.779 \|  \| \| \| presentation order + session + standard duration + session  ✻  standard duration \|  \| 0.006 \|  \| \| 2.814e  -8 \|  \| \| 4.672e  -6 \|  \| \| 26.136 \|  \| \| 3.785 \|  \| \| \| presentation order + session + standard duration + condition + presentation order  ✻  session \|  \| 0.006 \|  \| \| 2.398e  -8 \|  \| \| 3.981e  -6 \|  \| \| 22.275 \|  \| \| 3.905 \|  \| \| \| presentation order + session + standard duration + presentation order  ✻  session + session  ✻  standard duration \|  \| 0.006 \|  \| \| 1.940e  -8 \|  \| \| 3.220e  -6 \|  \| \| 18.013 \|  \| \| 2.824 \|  \| \| \| presentation order + standard duration + condition + presentation order  ✻  condition + standard duration  ✻  condition \|  \| 0.006 \|  \| \| 1.443e  -8 \|  \| \| 2.395e  -6 \|  \| \| 13.401 \|  \| \| 2.147 \|  \| \| \| presentation order + session + standard duration + condition + session  ✻  standard duration + presentation order  ✻  condition \|  \| 0.006 \|  \| \| 1.236e  -8 \|  \| \| 2.052e  -6 \|  \| \| 11.479 \|  \| \| 77.418 \|  \| \| \| presentation order + session + standard duration + condition + standard duration  ✻  condition \|  \| 0.006 \|  \| \| 1.118e  -8 \|  \| \| 1.856e  -6 \|  \| \| 10.383 \|  \| \| 7.986 \|  \| \| \| presentation order + session + condition + presentation order  ✻  condition + session  ✻  condition \|  \| 0.006 \|  \| \| 1.100e  -8 \|  \| \| 1.826e  -6 \|  \| \| 10.215 \|  \| \| 2.944 \|  \| \| \| presentation order + session + condition + presentation order  ✻  session + presentation order  ✻  condition + session  ✻  condition \|  \| 0.006 \|  \| \| 1.009e  -8 \|  \| \| 1.675e  -6 \|  \| \| 9.374 \|  \| \| 21.124 \|  \| \| \| presentation order + session + standard duration + condition + presentation order  ✻  session + presentation order  ✻  condition \|  \| 0.006 \|  \| \| 8.884e  -9 \|  \| \| 1.475e  -6 \|  \| \| 8.250 \|  \| \| 29.124 \|  \| \| \| presentation order + session + standard duration + condition + presentation order  ✻  condition \|  \| 0.006 \|  \| \| 8.788e  -9 \|  \| \| 1.459e  -6 \|  \| \| 8.161 \|  \| \| 2.560 \|  \| \| \| presentation order + session + standard duration + condition + session  ✻  standard duration \|  \| 0.006 \|  \| \| 8.544e  -9 \|  \| \| 1.418e  -6 \|  \| \| 7.935 \|  \| \| 7.332 \|  \| \| \| presentation order + session + standard duration + condition + session  ✻  condition \|  \| 0.006 \|  \| \| 8.388e  -9 \|  \| \| 1.392e  -6 \|  \| \| 7.790 \|  \| \| 5.837 \|  \| \| \| presentation order + session + condition + presentation order  ✻  session + presentation order  ✻  condition + session  ✻  condition + presentation order  ✻  session  ✻  condition \|  \| 0.006 \|  \| \| 8.266e  -9 \|  \| \| 1.372e  -6 \|  \| \| 7.676 \|  \| \| 15.430 \|  \| \| \| presentation order + session + standard duration + condition + presentation order  ✻  session + standard duration  ✻  condition \|  \| 0.006 \|  \| \| 7.294e  -9 \|  \| \| 1.211e  -6 \|  \| \| 6.774 \|  \| \| 7.707 \|  \| \| \| presentation order + session + standard duration + condition + presentation order  ✻  session + session  ✻  standard duration \|  \| 0.006 \|  \| \| 5.612e  -9 \|  \| \| 9.315e  -7 \|  \| \| 5.212 \|  \| \| 4.986 \|  \| \| \| presentation order + session + standard duration + condition + presentation order  ✻  session + session  ✻  condition \|  \| 0.006 \|  \| \| 5.403e  -9 \|  \| \| 8.969e  -7 \|  \| \| 5.018 \|  \| \| 3.174 \|  \| \| \| presentation order + session + standard duration + condition + presentation order  ✻  condition + standard duration  ✻  condition \|  \| 0.006 \|  \| \| 2.701e  -9 \|  \| \| 4.483e  -7 \|  \| \| 2.508 \|  \| \| 3.712 \|  \| \| \| presentation order + session + standard duration + condition + session  ✻  condition + standard duration  ✻  condition \|  \| 0.006 \|  \| \| 2.594e  -9 \|  \| \| 4.306e  -7 \|  \| \| 2.409 \|  \| \| 4.945 \|  \| \| \| presentation order + session + standard duration + condition + presentation order  ✻  condition + session  ✻  condition \|  \| 0.006 \|  \| \| 2.284e  -9 \|  \| \| 3.791e  -7 \|  \| \| 2.121 \|  \| \| 8.038 \|  \| \| \| presentation order + session + standard duration + condition + session  ✻  standard duration + standard duration  ✻  condition \|  \| 0.006 \|  \| \| 2.233e  -9 \|  \| \| 3.706e  -7 \|  \| \| 2.074 \|  \| \| 3.068 \|  \| \| \| presentation order + session + standard duration + condition + session  ✻  standard duration + session  ✻  condition \|  \| 0.006 \|  \| \| 2.003e  -9 \|  \| \| 3.325e  -7 \|  \| \| 1.860 \|  \| \| 5.604 \|  \| \| \| presentation order + session + standard duration + condition + presentation order  ✻  session + presentation order  ✻  condition + standard duration  ✻  condition \|  \| 0.006 \|  \| \| 1.882e  -9 \|  \| \| 3.125e  -7 \|  \| \| 1.748 \|  \| \| 5.433 \|  \| \| \| presentation order + session + standard duration + condition + presentation order  ✻  session + session  ✻  standard duration + standard duration  ✻  condition \|  \| 0.006 \|  \| \| 1.835e  -9 \|  \| \| 3.046e  -7 \|  \| \| 1.704 \|  \| \| 6.320 \|  \| \| \| presentation order + session + standard duration + condition + presentation order  ✻  session + presentation order  ✻  condition + session  ✻  condition \|  \| 0.006 \|  \| \| 1.632e  -9 \|  \| \| 2.709e  -7 \|  \| \| 1.516 \|  \| \| 5.189 \|  \| \| \| presentation order + session + standard duration + condition + presentation order  ✻  session + session  ✻  condition + standard duration  ✻  condition \|  \| 0.006 \|  \| \| 1.577e  -9 \|  \| \| 2.617e  -7 \|  \| \| 1.464 \|  \| \| 3.257 \|  \| \| \| presentation order + session + standard duration + condition + presentation order  ✻  session + session  ✻  standard duration + presentation order  ✻  condition \|  \| 0.006 \|  \| \| 1.554e  -9 \|  \| \| 2.580e  -7 \|  \| \| 1.443 \|  \| \| 4.538 \|  \| \| \| presentation order + session + standard duration + condition + presentation order  ✻  session + presentation order  ✻  condition + session  ✻  condition + presentation order  ✻  session  ✻  condition \|  \| 0.006 \|  \| \| 1.403e  -9 \|  \| \| 2.329e  -7 \|  \| \| 1.303 \|  \| \| 11.873 \|  \| \| \| presentation order + session + standard duration + condition + presentation order  ✻  session + session  ✻  standard duration + session  ✻  condition \|  \| 0.006 \|  \| \| 1.293e  -9 \|  \| \| 2.146e  -7 \|  \| \| 1.200 \|  \| \| 3.958 \|  \| \| \| presentation order + session + standard duration + condition + presentation order  ✻  condition + session  ✻  condition + standard duration  ✻  condition \|  \| 0.006 \|  \| \| 6.723e -10 \|  \| \| 1.116e  -7 \|  \| \| 0.624 \|  \| \| 6.524 \|  \| \| \| presentation order + session + standard duration + condition + session  ✻  standard duration + presentation order  ✻  condition + session  ✻  condition \|  \| 0.006 \|  \| \| 5.770e -10 \|  \| \| 9.578e  -8 \|  \| \| 0.536 \|  \| \| 8.859 \|  \| \| \| presentation order + session + standard duration + condition + session  ✻  standard duration + presentation order  ✻  condition + standard duration  ✻  condition \|  \| 0.006 \|  \| \| 5.757e -10 \|  \| \| 9.556e  -8 \|  \| \| 0.535 \|  \| \| 6.768 \|  \| \| \| presentation order + session + standard duration + condition + session  ✻  standard duration + session  ✻  condition + standard duration  ✻  condition \|  \| 0.006 \|  \| \| 5.517e -10 \|  \| \| 9.158e  -8 \|  \| \| 0.512 \|  \| \| 3.674 \|  \| \| \| presentation order + session + standard duration + condition + presentation order  ✻  session + presentation order  ✻  condition + session  ✻  condition + standard duration  ✻  condition \|  \| 0.006 \|  \| \| 4.700e -10 \|  \| \| 7.802e  -8 \|  \| \| 0.437 \|  \| \| 5.324 \|  \| \| \| presentation order + session + standard duration + condition + presentation order  ✻  session + session  ✻  standard duration + presentation order  ✻  condition + standard duration  ✻  condition \|  \| 0.006 \|  \| \| 4.577e -10 \|  \| \| 7.598e  -8 \|  \| \| 0.425 \|  \| \| 5.868 \|  \| \| \| presentation order + session + standard duration + condition + presentation order  ✻  session + session  ✻  standard duration + presentation order  ✻  condition + session  ✻  condition \|  \| 0.006 \|  \| \| 4.421e -10 \|  \| \| 7.340e  -8 \|  \| \| 0.411 \|  \| \| 19.531 \|  \| \| \| presentation order + session + standard duration + condition + presentation order  ✻  session + presentation order  ✻  condition + session  ✻  condition + standard duration  ✻  condition + presentation order  ✻  session  ✻  condition \|  \| 0.006 \|  \| \| 4.194e -10 \|  \| \| 6.961e  -8 \|  \| \| 0.389 \|  \| \| 17.122 \|  \| \| \| presentation order + session + standard duration + condition + presentation order  ✻  session + session  ✻  standard duration + session  ✻  condition + standard duration  ✻  condition \|  \| 0.006 \|  \| \| 4.004e -10 \|  \| \| 6.647e  -8 \|  \| \| 0.372 \|  \| \| 6.071 \|  \| \| \| presentation order + session + standard duration + condition + presentation order  ✻  session + session  ✻  standard duration + presentation order  ✻  condition + session  ✻  condition + presentation order  ✻  session  ✻  condition \|  \| 0.006 \|  \| \| 3.042e -10 \|  \| \| 5.050e  -8 \|  \| \| 0.283 \|  \| \| 4.413 \|  \| \| \| condition \|  \| 0.006 \|  \| \| 2.859e -10 \|  \| \| 4.747e  -8 \|  \| \| 0.266 \|  \| \| 1.242 \|  \| \| \| presentation order + session + standard duration + condition + session  ✻  standard duration + session  ✻  condition + standard duration  ✻  condition + session  ✻  standard duration  ✻  condition \|  \| 0.006 \|  \| \| 2.212e -10 \|  \| \| 3.672e  -8 \|  \| \| 0.205 \|  \| \| 6.452 \|  \| \| \| standard duration \|  \| 0.006 \|  \| \| 2.093e -10 \|  \| \| 3.475e  -8 \|  \| \| 0.194 \|  \| \| 2.088 \|  \| \| \| session \|  \| 0.006 \|  \| \| 1.982e -10 \|  \| \| 3.290e  -8 \|  \| \| 0.184 \|  \| \| 2.190 \|  \| \| \| presentation order + session + standard duration + condition + session  ✻  standard duration + presentation order  ✻  condition + session  ✻  condition + standard duration  ✻  condition \|  \| 0.006 \|  \| \| 1.917e -10 \|  \| \| 3.182e  -8 \|  \| \| 0.178 \|  \| \| 19.598 \|  \| \| \| presentation order + session + standard duration + condition + presentation order  ✻  session + session  ✻  standard duration + session  ✻  condition + standard duration  ✻  condition + session  ✻  standard duration  ✻  condition \|  \| 0.006 \|  \| \| 1.564e -10 \|  \| \| 2.597e  -8 \|  \| \| 0.145 \|  \| \| 11.075 \|  \| \| \| presentation order + session + standard duration + condition + presentation order  ✻  session + session  ✻  standard duration + presentation order  ✻  condition + session  ✻  condition + standard duration  ✻  condition \|  \| 0.006 \|  \| \| 1.245e -10 \|  \| \| 2.066e  -8 \|  \| \| 0.116 \|  \| \| 14.918 \|  \| \| \| presentation order + session + standard duration + condition + presentation order  ✻  session + session  ✻  standard duration + presentation order  ✻  condition + session  ✻  condition + standard duration  ✻  condition + presentation order  ✻  session  ✻  condition \|  \| 0.006 \|  \| \| 9.549e -11 \|  \| \| 1.585e  -8 \|  \| \| 0.089 \|  \| \| 16.635 \|  \| \| \| session + condition \|  \| 0.006 \|  \| \| 5.702e -11 \|  \| \| 9.465e  -9 \|  \| \| 0.053 \|  \| \| 8.973 \|  \| \| \| standard duration + condition \|  \| 0.006 \|  \| \| 5.404e -11 \|  \| \| 8.971e  -9 \|  \| \| 0.050 \|  \| \| 1.886 \|  \| \| \| presentation order + session + standard duration + condition + session  ✻  standard duration + presentation order  ✻  condition + session  ✻  condition + standard duration  ✻  condition + session  ✻  standard duration  ✻  condition \|  \| 0.006 \|  \| \| 5.396e -11 \|  \| \| 8.958e  -9 \|  \| \| 0.050 \|  \| \| 4.721 \|  \| \| \| presentation order + session + standard duration + condition + presentation order  ✻  session + session  ✻  standard duration + presentation order  ✻  condition + session  ✻  condition + standard duration  ✻  condition + session  ✻  standard duration  ✻  condition \|  \| 0.006 \|  \| \| 5.340e -11 \|  \| \| 8.864e  -9 \|  \| \| 0.050 \|  \| \| 19.111 \|  \| \| \| session + standard duration \|  \| 0.006 \|  \| \| 3.831e -11 \|  \| \| 6.360e  -9 \|  \| \| 0.036 \|  \| \| 3.339 \|  \| \| \| presentation order + session + standard duration + condition + presentation order  ✻  session + session  ✻  standard duration + presentation order  ✻  condition + session  ✻  condition + standard duration  ✻  condition + presentation order  ✻  session  ✻  condition + session  ✻  standard duration  ✻  condition \|  \| 0.006 \|  \| \| 3.522e -11 \|  \| \| 5.846e  -9 \|  \| \| 0.033 \|  \| \| 8.903 \|  \| \| \| standard duration + condition + standard duration  ✻  condition \|  \| 0.006 \|  \| \| 1.529e -11 \|  \| \| 2.539e  -9 \|  \| \| 0.014 \|  \| \| 1.850 \|  \| \| \| session + condition + session  ✻  condition \|  \| 0.006 \|  \| \| 1.214e -11 \|  \| \| 2.015e  -9 \|  \| \| 0.011 \|  \| \| 2.270 \|  \| \| \| session + standard duration + condition \|  \| 0.006 \|  \| \| 9.901e -12 \|  \| \| 1.644e  -9 \|  \| \| 0.009 \|  \| \| 2.373 \|  \| \| \| session + standard duration + session  ✻  standard duration \|  \| 0.006 \|  \| \| 8.916e -12 \|  \| \| 1.480e  -9 \|  \| \| 0.008 \|  \| \| 3.447 \|  \| \| \| session + standard duration + condition + standard duration  ✻  condition \|  \| 0.006 \|  \| \| 2.936e -12 \|  \| \| 4.874e -10 \|  \| \| 0.003 \|  \| \| 5.255 \|  \| \| \| session + standard duration + condition + session  ✻  standard duration \|  \| 0.006 \|  \| \| 2.717e -12 \|  \| \| 4.510e -10 \|  \| \| 0.003 \|  \| \| 7.251 \|  \| \| \| session + standard duration + condition + session  ✻  condition \|  \| 0.006 \|  \| \| 2.401e -12 \|  \| \| 3.985e -10 \|  \| \| 0.002 \|  \| \| 2.457 \|  \| \| \| session + standard duration + condition + session  ✻  standard duration + standard duration  ✻  condition \|  \| 0.006 \|  \| \| 7.208e -13 \|  \| \| 1.197e -10 \|  \| \| 6.695e -4 \|  \| \| 4.568 \|  \| \| \| session + standard duration + condition + session  ✻  condition + standard duration  ✻  condition \|  \| 0.006 \|  \| \| 7.007e -13 \|  \| \| 1.163e -10 \|  \| \| 6.508e -4 \|  \| \| 7.395 \|  \| \| \| session + standard duration + condition + session  ✻  standard duration + session  ✻  condition \|  \| 0.006 \|  \| \| 5.893e -13 \|  \| \| 9.782e -11 \|  \| \| 5.472e -4 \|  \| \| 5.170 \|  \| \| \| session + standard duration + condition + session  ✻  standard duration + session  ✻  condition + standard duration  ✻  condition \|  \| 0.006 \|  \| \| 1.615e -13 \|  \| \| 2.680e -11 \|  \| \| 1.499e -4 \|  \| \| 4.307 \|  \| \| \| session + standard duration + condition + session  ✻  standard duration + session  ✻  condition + standard duration  ✻  condition + session  ✻  standard duration  ✻  condition \|  \| 0.006 \|  \| \| 5.681e -14 \|  \| \| 9.431e -12 \|  \| \| 5.276e -5 \|  \| \| 4.085 \|  \| \| \|  \| \| \| \| \| \| \| \| \| \| \| \| \| \| \| \| \| *Note.*  All models include subject \| \| \| \| \| \| \| \| \| \| \| \| \| \| \| \| |
| --- | --- | --- | --- | --- | --- | --- | --- | --- | --- | --- | --- | --- | --- | --- | --- | --- | --- | --- | --- | --- | --- | --- | --- | --- | --- | --- | --- | --- | --- | --- | --- | --- | --- | --- | --- | --- | --- | --- | --- | --- | --- | --- | --- | --- | --- | --- | --- | --- | --- | --- | --- | --- | --- | --- | --- | --- | --- | --- | --- | --- | --- | --- | --- | --- | --- | --- | --- | --- | --- | --- | --- | --- | --- | --- | --- | --- | --- | --- | --- | --- | --- | --- | --- | --- | --- | --- | --- | --- | --- | --- | --- | --- | --- | --- | --- | --- | --- | --- | --- | --- | --- | --- | --- | --- | --- | --- | --- | --- | --- | --- | --- | --- | --- | --- | --- | --- | --- | --- | --- | --- | --- | --- | --- | --- | --- | --- | --- | --- | --- | --- | --- | --- | --- | --- | --- | --- | --- | --- | --- | --- | --- | --- | --- | --- | --- | --- | --- | --- | --- | --- | --- | --- | --- | --- | --- | --- | --- | --- | --- | --- | --- | --- | --- | --- | --- | --- | --- | --- | --- | --- | --- | --- | --- | --- | --- | --- | --- | --- | --- | --- | --- | --- | --- | --- | --- | --- | --- | --- | --- | --- | --- | --- | --- | --- | --- | --- | --- | --- | --- | --- | --- | --- | --- | --- | --- | --- | --- | --- | --- | --- | --- | --- | --- | --- | --- | --- | --- | --- | --- | --- | --- | --- | --- | --- | --- | --- | --- | --- | --- | --- | --- | --- | --- | --- | --- | --- | --- | --- | --- | --- | --- | --- | --- | --- | --- | --- | --- | --- | --- | --- | --- | --- | --- | --- | --- | --- | --- | --- | --- | --- | --- | --- | --- | --- | --- | --- | --- | --- | --- | --- | --- | --- | --- | --- | --- | --- | --- | --- | --- | --- | --- | --- | --- | --- | --- | --- | --- | --- | --- | --- | --- | --- | --- | --- | --- | --- | --- | --- | --- | --- | --- | --- | --- | --- | --- | --- | --- | --- | --- | --- | --- | --- | --- | --- | --- | --- | --- | --- | --- | --- | --- | --- | --- | --- | --- | --- | --- | --- | --- | --- | --- | --- | --- | --- | --- | --- | --- | --- | --- | --- | --- | --- | --- | --- | --- | --- | --- | --- | --- | --- | --- | --- | --- | --- | --- | --- | --- | --- | --- | --- | --- | --- | --- | --- | --- | --- | --- | --- | --- | --- | --- | --- | --- | --- | --- | --- | --- | --- | --- | --- | --- | --- | --- | --- | --- | --- | --- | --- | --- | --- | --- | --- | --- | --- | --- | --- | --- | --- | --- | --- | --- | --- | --- | --- | --- | --- | --- | --- | --- | --- | --- | --- | --- | --- | --- | --- | --- | --- | --- | --- | --- | --- | --- | --- | --- | --- | --- | --- | --- | --- | --- | --- | --- | --- | --- | --- | --- | --- | --- | --- | --- | --- | --- | --- | --- | --- | --- | --- | --- | --- | --- | --- | --- | --- | --- | --- | --- | --- | --- | --- | --- | --- | --- | --- | --- | --- | --- | --- | --- | --- | --- | --- | --- | --- | --- | --- | --- | --- | --- | --- | --- | --- | --- | --- | --- | --- | --- | --- | --- | --- | --- | --- | --- | --- | --- | --- | --- | --- | --- | --- | --- | --- | --- | --- | --- | --- | --- | --- | --- | --- | --- | --- | --- | --- | --- | --- | --- | --- | --- | --- | --- | --- | --- | --- | --- | --- | --- | --- | --- | --- | --- | --- | --- | --- | --- | --- | --- | --- | --- | --- | --- | --- | --- | --- | --- | --- | --- | --- | --- | --- | --- | --- | --- | --- | --- | --- | --- | --- | --- | --- | --- | --- | --- | --- | --- | --- | --- | --- | --- | --- | --- | --- | --- | --- | --- | --- | --- | --- | --- | --- | --- | --- | --- | --- | --- | --- | --- | --- | --- | --- | --- | --- | --- | --- | --- | --- | --- | --- | --- | --- | --- | --- | --- | --- | --- | --- | --- | --- | --- | --- | --- | --- | --- | --- | --- | --- | --- | --- | --- | --- | --- | --- | --- | --- | --- | --- | --- | --- | --- | --- | --- | --- | --- | --- | --- | --- | --- | --- | --- | --- | --- | --- | --- | --- | --- | --- | --- | --- | --- | --- | --- | --- | --- | --- | --- | --- | --- | --- | --- | --- | --- | --- | --- | --- | --- | --- | --- | --- | --- | --- | --- | --- | --- | --- | --- | --- | --- | --- | --- | --- | --- | --- | --- | --- | --- | --- | --- | --- | --- | --- | --- | --- | --- | --- | --- | --- | --- | --- | --- | --- | --- | --- | --- | --- | --- | --- | --- | --- | --- | --- | --- | --- | --- | --- | --- | --- | --- | --- | --- | --- | --- | --- | --- | --- | --- | --- | --- | --- | --- | --- | --- | --- | --- | --- | --- | --- | --- | --- | --- | --- | --- | --- | --- | --- | --- | --- | --- | --- | --- | --- | --- | --- | --- | --- | --- | --- | --- | --- | --- | --- | --- | --- | --- | --- | --- | --- | --- | --- | --- | --- | --- | --- | --- | --- | --- | --- | --- | --- | --- | --- | --- | --- | --- | --- | --- | --- | --- | --- | --- | --- | --- | --- | --- | --- | --- | --- | --- | --- | --- | --- | --- | --- | --- | --- | --- | --- | --- | --- | --- | --- | --- | --- | --- | --- | --- | --- | --- | --- | --- | --- | --- | --- | --- | --- | --- | --- | --- | --- | --- | --- | --- | --- | --- | --- | --- | --- | --- | --- | --- | --- | --- | --- | --- | --- | --- | --- | --- | --- | --- | --- | --- | --- | --- | --- | --- | --- | --- | --- | --- | --- | --- | --- | --- | --- | --- | --- | --- | --- | --- | --- | --- | --- | --- | --- | --- | --- | --- | --- | --- | --- | --- | --- | --- | --- | --- | --- | --- | --- | --- | --- | --- | --- | --- | --- | --- | --- | --- | --- | --- | --- | --- | --- | --- | --- | --- | --- | --- | --- | --- | --- | --- | --- | --- | --- | --- | --- | --- | --- | --- | --- | --- | --- | --- | --- | --- | --- | --- | --- | --- | --- | --- | --- | --- | --- | --- | --- | --- | --- | --- | --- | --- | --- | --- | --- | --- | --- | --- | --- | --- | --- | --- | --- | --- | --- | --- | --- | --- | --- | --- | --- | --- | --- | --- | --- | --- | --- | --- | --- | --- | --- | --- | --- | --- | --- | --- | --- | --- | --- | --- | --- | --- | --- | --- | --- | --- | --- | --- | --- | --- | --- | --- | --- | --- | --- | --- | --- | --- | --- | --- | --- | --- | --- | --- | --- | --- | --- | --- | --- | --- | --- | --- | --- | --- | --- | --- | --- | --- | --- | --- | --- | --- | --- | --- | --- | --- | --- | --- | --- | --- | --- | --- | --- | --- | --- | --- | --- | --- | --- | --- | --- | --- | --- | --- | --- | --- | --- | --- | --- | --- | --- | --- | --- | --- | --- | --- | --- | --- | --- | --- | --- | --- | --- | --- | --- | --- | --- | --- | --- | --- | --- | --- | --- | --- | --- | --- | --- | --- | --- | --- | --- | --- | --- | --- | --- | --- | --- | --- | --- | --- | --- | --- | --- | --- | --- | --- | --- | --- | --- | --- | --- | --- | --- | --- | --- | --- | --- | --- | --- | --- | --- | --- | --- | --- | --- | --- | --- | --- | --- | --- | --- | --- | --- | --- | --- | --- | --- | --- | --- | --- | --- | --- | --- | --- | --- | --- | --- | --- | --- | --- | --- | --- | --- | --- | --- | --- | --- | --- | --- | --- | --- | --- | --- | --- | --- | --- | --- | --- | --- | --- | --- | --- | --- | --- | --- | --- | --- | --- | --- | --- | --- | --- | --- | --- | --- | --- | --- | --- | --- | --- | --- | --- | --- | --- | --- | --- | --- | --- | --- | --- | --- | --- | --- | --- | --- | --- | --- | --- | --- | --- | --- | --- | --- | --- | --- | --- | --- | --- | --- | --- | --- | --- | --- | --- | --- | --- | --- | --- | --- | --- | --- | --- | --- | --- | --- | --- | --- | --- | --- | --- | --- | --- | --- | --- | --- | --- | --- | --- | --- | --- | --- | --- | --- | --- | --- | --- | --- | --- | --- | --- | --- | --- | --- | --- | --- | --- | --- | --- | --- | --- | --- | --- | --- | --- | --- | --- | --- | --- | --- | --- | --- | --- | --- | --- | --- | --- | --- | --- | --- | --- | --- | --- | --- | --- | --- | --- | --- | --- | --- | --- | --- | --- | --- | --- | --- | --- | --- | --- | --- | --- | --- | --- | --- | --- | --- | --- | --- | --- | --- | --- | --- | --- | --- | --- | --- | --- | --- | --- | --- | --- | --- | --- | --- | --- | --- | --- | --- | --- | --- | --- | --- | --- | --- | --- | --- | --- | --- | --- | --- | --- | --- | --- | --- | --- | --- | --- | --- | --- | --- | --- | --- | --- | --- | --- | --- | --- | --- | --- | --- | --- | --- | --- | --- | --- | --- | --- | --- | --- | --- | --- | --- | --- | --- | --- | --- | --- | --- | --- | --- | --- | --- | --- | --- | --- | --- | --- | --- | --- | --- | --- | --- | --- | --- | --- | --- | --- | --- | --- | --- | --- | --- | --- | --- | --- | --- | --- | --- | --- | --- | --- | --- | --- | --- | --- | --- | --- | --- | --- | --- | --- | --- | --- | --- | --- | --- | --- | --- | --- | --- | --- | --- | --- | --- | --- | --- | --- | --- | --- | --- | --- | --- | --- | --- | --- | --- | --- | --- | --- | --- | --- | --- | --- | --- | --- | --- | --- | --- | --- | --- | --- | --- | --- | --- | --- | --- | --- | --- | --- | --- | --- | --- | --- | --- | --- | --- | --- | --- | --- | --- | --- | --- | --- | --- | --- | --- | --- | --- | --- | --- | --- | --- | --- | --- | --- | --- | --- | --- | --- | --- | --- | --- | --- | --- | --- | --- | --- | --- | --- | --- | --- | --- | --- | --- | --- | --- | --- | --- | --- | --- | --- | --- | --- | --- | --- | --- | --- | --- | --- | --- | --- | --- | --- | --- | --- | --- | --- | --- | --- | --- | --- | --- | --- | --- | --- | --- | --- | --- | --- | --- | --- | --- | --- | --- | --- | --- | --- | --- | --- | --- | --- | --- | --- | --- | --- | --- | --- | --- | --- | --- | --- | --- | --- | --- | --- | --- | --- | --- | --- | --- | --- | --- | --- | --- | --- | --- | --- | --- | --- | --- | --- | --- | --- | --- | --- | --- | --- | --- | --- | --- | --- | --- | --- | --- | --- | --- | --- | --- | --- | --- | --- | --- | --- | --- | --- | --- | --- | --- | --- | --- | --- | --- | --- | --- | --- | --- | --- | --- | --- | --- | --- | --- | --- | --- | --- | --- | --- | --- | --- | --- | --- | --- | --- | --- | --- | --- | --- | --- | --- | --- | --- | --- | --- | --- | --- | --- | --- | --- | --- | --- | --- | --- | --- | --- | --- | --- | --- | --- | --- | --- | --- | --- | --- | --- | --- | --- | --- | --- | --- | --- | --- | --- | --- | --- | --- | --- | --- | --- | --- | --- | --- | --- | --- | --- | --- | --- | --- | --- | --- | --- | --- | --- | --- | --- | --- | --- | --- | --- | --- | --- | --- | --- | --- | --- | --- | --- | --- | --- | --- | --- | --- | --- | --- | --- | --- | --- | --- | --- | --- | --- | --- | --- | --- | --- | --- | --- | --- | --- | --- | --- | --- | --- | --- | --- | --- | --- | --- | --- | --- | --- | --- | --- | --- | --- | --- | --- | --- | --- | --- | --- | --- | --- | --- | --- | --- | --- | --- | --- | --- | --- | --- | --- | --- | --- | --- | --- | --- | --- | --- | --- | --- | --- | --- | --- | --- | --- | --- | --- | --- | --- | --- | --- | --- | --- | --- | --- | --- | --- | --- | --- | --- | --- | --- | --- | --- | --- | --- | --- | --- | --- | --- | --- | --- | --- | --- | --- | --- | --- | --- | --- | --- | --- | --- | --- | --- | --- | --- | --- | --- | --- | --- | --- | --- | --- | --- | --- | --- | --- | --- | --- | --- | --- | --- | --- | --- | --- | --- | --- | --- | --- | --- | --- | --- | --- | --- | --- | --- | --- | --- | --- | --- | --- | --- | --- | --- | --- | --- | --- | --- | --- | --- | --- | --- | --- | --- | --- | --- | --- | --- | --- | --- | --- | --- | --- | --- | --- | --- | --- | --- | --- | --- | --- | --- | --- | --- | --- | --- | --- | --- | --- | --- | --- | --- | --- | --- | --- | --- | --- | --- | --- | --- | --- | --- | --- | --- | --- | --- | --- | --- | --- | --- | --- | --- | --- | --- | --- | --- | --- | --- | --- | --- | --- | --- | --- | --- | --- | --- | --- | --- | --- | --- | --- | --- | --- | --- | --- | --- | --- | --- | --- | --- | --- | --- | --- | --- | --- | --- | --- | --- | --- | --- | --- | --- | --- | --- | --- | --- | --- | --- | --- | --- | --- | --- | --- | --- | --- | --- | --- | --- | --- | --- | --- | --- | --- | --- | --- | --- | --- | --- | --- | --- | --- | --- | --- | --- | --- | --- | --- | --- | --- | --- | --- | --- | --- | --- | --- | --- | --- | --- | --- | --- | --- | --- | --- | --- | --- | --- | --- | --- | --- | --- | --- | --- | --- | --- | --- | --- | --- | --- | --- | --- | --- | --- | --- | --- | --- | --- | --- | --- | --- | --- | --- | --- | --- | --- | --- | --- | --- | --- | --- | --- | --- | --- | --- | --- | --- | --- | --- | --- | --- | --- | --- | --- | --- | --- | --- | --- | --- | --- | --- | --- | --- | --- | --- | --- | --- | --- | --- | --- | --- | --- | --- | --- | --- | --- | --- | --- | --- | --- | --- | --- | --- | --- | --- | --- | --- | --- | --- | --- | --- | --- | --- | --- | --- | --- | --- | --- | --- | --- | --- | --- | --- | --- | --- | --- | --- | --- | --- | --- | --- | --- | --- | --- | --- | --- | --- | --- | --- | --- | --- | --- | --- | --- | --- | --- | --- | --- | --- | --- | --- | --- | --- | --- | --- | --- | --- | --- | --- | --- | --- | --- | --- | --- | --- | --- | --- | --- | --- | --- | --- | --- | --- | --- | --- | --- | --- | --- | --- | --- | --- | --- | --- | --- | --- | --- | --- | --- | --- | --- | --- | --- | --- | --- | --- | --- | --- | --- | --- | --- | --- | --- | --- | --- | --- | --- | --- | --- | --- | --- | --- | --- | --- | --- | --- | --- | --- | --- | --- | --- | --- | --- | --- | --- | --- | --- | --- | --- | --- | --- | --- | --- | --- | --- | --- | --- | --- | --- | --- | --- | --- | --- | --- | --- | --- | --- | --- | --- | --- | --- | --- | --- | --- | --- | --- | --- | --- | --- | --- | --- | --- | --- | --- | --- | --- | --- | --- | --- | --- | --- | --- | --- | --- | --- | --- | --- | --- | --- | --- | --- | --- | --- | --- | --- | --- | --- | --- | --- | --- | --- | --- | --- | --- | --- | --- | --- | --- | --- | --- | --- | --- | --- | --- | --- | --- | --- | --- | --- | --- | --- | --- | --- | --- | --- | --- | --- | --- | --- | --- | --- | --- | --- | --- | --- | --- | --- | --- | --- | --- | --- | --- | --- | --- | --- | --- | --- | --- | --- | --- | --- | --- | --- | --- | --- | --- | --- | --- | --- | --- | --- | --- | --- | --- | --- | --- | --- | --- | --- | --- | --- | --- | --- | --- | --- | --- | --- | --- | --- | --- | --- | --- | --- | --- | --- | --- | --- | --- | --- | --- | --- | --- | --- | --- | --- | --- | --- | --- | --- | --- | --- | --- | --- | --- | --- | --- | --- | --- | --- | --- | --- | --- | --- | --- | --- | --- | --- | --- | --- | --- | --- | --- | --- | --- | --- | --- | --- | --- | --- | --- | --- | --- | --- | --- | --- | --- | --- | --- | --- | --- | --- | --- | --- | --- | --- | --- | --- | --- | --- | --- | --- | --- | --- | --- | --- | --- | --- | --- | --- | --- | --- | --- | --- | --- | --- | --- | --- | --- | --- | --- | --- | --- | --- | --- | --- | --- | --- | --- | --- | --- | --- | --- | --- | --- | --- | --- | --- | --- | --- | --- | --- | --- | --- | --- | --- | --- | --- | --- | --- | --- | --- | --- | --- | --- | --- | --- | --- | --- | --- | --- | --- | --- | --- | --- | --- | --- | --- | --- | --- | --- | --- | --- | --- | --- | --- | --- | --- | --- | --- | --- | --- | --- | --- | --- | --- | --- | --- | --- | --- | --- | --- | --- | --- | --- | --- | --- | --- | --- | --- | --- | --- | --- | --- | --- | --- | --- | --- | --- | --- | --- | --- | --- | --- | --- | --- | --- | --- | --- | --- | --- | --- | --- | --- | --- | --- | --- | --- | --- | --- | --- | --- | --- | --- | --- | --- | --- | --- | --- | --- | --- | --- | --- | --- | --- | --- | --- | --- | --- | --- | --- | --- | --- | --- | --- | --- | --- | --- | --- | --- | --- | --- | --- | --- | --- | --- | --- | --- | --- | --- | --- | --- | --- | --- | --- | --- | --- | --- | --- | --- | --- | --- | --- | --- | --- | --- | --- | --- | --- | --- | --- | --- | --- | --- | --- | --- | --- | --- | --- | --- | --- | --- | --- | --- | --- | --- | --- | --- | --- | --- | --- | --- | --- | --- | --- | --- | --- | --- | --- | --- | --- | --- | --- | --- | --- | --- | --- | --- | --- | --- | --- | --- | --- | --- | --- | --- | --- | --- | --- | --- | --- | --- | --- | --- | --- | --- | --- | --- | --- | --- | --- | --- | --- | --- | --- | --- | --- | --- | --- | --- | --- | --- | --- | --- | --- | --- | --- | --- | --- | --- | --- | --- | --- | --- | --- | --- | --- | --- | --- | --- | --- | --- | --- | --- | --- | --- | --- | --- | --- | --- | --- | --- | --- | --- | --- | --- | --- | --- | --- | --- | --- | --- | --- | --- | --- | --- | --- | --- | --- | --- | --- | --- | --- | --- | --- | --- | --- | --- | --- | --- | --- | --- | --- | --- | --- | --- | --- | --- | --- | --- | --- | --- | --- | --- | --- | --- | --- | --- | --- | --- | --- | --- | --- | --- | --- | --- | --- | --- | --- | --- | --- | --- | --- | --- | --- | --- | --- | --- | --- | --- | --- | --- | --- | --- | --- | --- | --- | --- | --- | --- | --- | --- | --- | --- | --- | --- | --- | --- | --- | --- | --- | --- | --- | --- | --- | --- | --- | --- | --- | --- | --- | --- | --- | --- | --- | --- | --- | --- | --- | --- | --- | --- | --- | --- | --- | --- | --- | --- | --- | --- | --- | --- | --- | --- | --- | --- | --- | --- | --- | --- | --- | --- | --- | --- | --- | --- | --- | --- | --- | --- | --- | --- | --- | --- | --- | --- | --- | --- | --- | --- | --- | --- | --- | --- | --- | --- | --- | --- | --- | --- | --- | --- | --- | --- | --- | --- | --- | --- | --- | --- | --- | --- | --- | --- | --- | --- | --- | --- | --- | --- | --- | --- | --- | --- | --- | --- | --- | --- |

**Table 1** Model comparisons for biases in Experiment 1. *Note.* P(M) represents the prior for each model. P(M|data) represents how much the data were explained by the model. BF_M_ represents how much the data was likely under the alternative hypothesis than the null hypothesis. BF_10_ represents the Bayesian factor. Note that all models include subjects as a variable.

| **Model Comparison** | | | | | | | | | | | |
| --- | --- | --- | --- | --- | --- | --- | --- | --- | --- | --- | --- |
| **Models** | | **P(M)** | | **P(M\|data)** | | **BF _M_** | | **BF _10_** | | **error %** | |
| Null model (incl. subject) |  | 0.006 |  | 2.792e -10 |  | 4.634e  -8 |  | 1.000 |  |  |  |
| presentation order + standard duration + condition + presentation order  ✻  condition |  | 0.006 |  | 0.193 |  | 39.647 |  | 6.906e +8 |  | 5.160 |  |
| presentation order + standard duration |  | 0.006 |  | 0.117 |  | 22.066 |  | 4.203e +8 |  | 3.260 |  |
| presentation order + standard duration + condition + presentation order  ✻  condition + standard duration  ✻  condition |  | 0.006 |  | 0.094 |  | 17.207 |  | 3.364e +8 |  | 2.513 |  |
| presentation order + standard duration + condition + presentation order  ✻  standard duration + presentation order  ✻  condition |  | 0.006 |  | 0.052 |  | 9.098 |  | 1.861e +8 |  | 2.455 |  |
| presentation order + standard duration + condition |  | 0.006 |  | 0.049 |  | 8.534 |  | 1.752e +8 |  | 3.476 |  |
| presentation order + standard duration + presentation order  ✻  standard duration |  | 0.006 |  | 0.044 |  | 7.598 |  | 1.568e +8 |  | 19.505 |  |
| presentation order + session + standard duration + condition + presentation order  ✻  standard duration |  | 0.006 |  | 0.041 |  | 7.158 |  | 1.481e +8 |  | 93.276 |  |
| presentation order + session + standard duration + condition + presentation order  ✻  condition |  | 0.006 |  | 0.040 |  | 6.866 |  | 1.423e +8 |  | 5.758 |  |
| presentation order + standard duration + condition + standard duration  ✻  condition |  | 0.006 |  | 0.035 |  | 6.087 |  | 1.267e +8 |  | 30.966 |  |
| presentation order + standard duration + condition + presentation order  ✻  standard duration + presentation order  ✻  condition + standard duration  ✻  condition |  | 0.006 |  | 0.030 |  | 5.219 |  | 1.092e +8 |  | 5.244 |  |
| presentation order + session + standard duration |  | 0.006 |  | 0.024 |  | 4.153 |  | 8.743e +7 |  | 2.681 |  |
| presentation order + session + standard duration + condition + presentation order  ✻  condition + standard duration  ✻  condition |  | 0.006 |  | 0.021 |  | 3.601 |  | 7.605e +7 |  | 4.055 |  |
| presentation order + session + standard duration + condition + presentation order  ✻  condition + session  ✻  condition |  | 0.006 |  | 0.016 |  | 2.630 |  | 5.588e +7 |  | 3.389 |  |
| presentation order + standard duration + condition + presentation order  ✻  standard duration |  | 0.006 |  | 0.014 |  | 2.306 |  | 4.908e +7 |  | 2.221 |  |
| presentation order + session + standard duration + condition + presentation order  ✻  standard duration + presentation order  ✻  condition |  | 0.006 |  | 0.012 |  | 2.018 |  | 4.302e +7 |  | 9.345 |  |
| presentation order + condition + presentation order  ✻  condition |  | 0.006 |  | 0.011 |  | 1.923 |  | 4.103e +7 |  | 2.621 |  |
| presentation order + session + standard duration + condition + presentation order  ✻  session + presentation order  ✻  condition |  | 0.006 |  | 0.010 |  | 1.691 |  | 3.613e +7 |  | 2.593 |  |
| presentation order + session + standard duration + condition |  | 0.006 |  | 0.010 |  | 1.651 |  | 3.528e +7 |  | 6.096 |  |
| presentation order + session + standard duration + condition + presentation order  ✻  condition + session  ✻  condition + standard duration  ✻  condition |  | 0.006 |  | 0.009 |  | 1.582 |  | 3.382e +7 |  | 10.798 |  |
| presentation order |  | 0.006 |  | 0.009 |  | 1.563 |  | 3.342e +7 |  | 5.965 |  |
| presentation order + standard duration + condition + presentation order  ✻  standard duration + presentation order  ✻  condition + standard duration  ✻  condition + presentation order  ✻  standard duration  ✻  condition |  | 0.006 |  | 0.009 |  | 1.557 |  | 3.328e +7 |  | 3.541 |  |
| presentation order + session + standard duration + condition + session  ✻  standard duration + presentation order  ✻  condition |  | 0.006 |  | 0.009 |  | 1.553 |  | 3.320e +7 |  | 5.621 |  |
| presentation order + session + standard duration + presentation order  ✻  session |  | 0.006 |  | 0.007 |  | 1.209 |  | 2.591e +7 |  | 7.890 |  |
| presentation order + standard duration + condition + presentation order  ✻  standard duration + standard duration  ✻  condition |  | 0.006 |  | 0.007 |  | 1.204 |  | 2.579e +7 |  | 2.984 |  |
| presentation order + session + standard duration + presentation order  ✻  standard duration |  | 0.006 |  | 0.007 |  | 1.126 |  | 2.414e +7 |  | 3.872 |  |
| presentation order + session + standard duration + condition + presentation order  ✻  session + presentation order  ✻  condition + standard duration  ✻  condition |  | 0.006 |  | 0.007 |  | 1.087 |  | 2.331e +7 |  | 4.889 |  |
| presentation order + session + standard duration + session  ✻  standard duration |  | 0.006 |  | 0.005 |  | 0.913 |  | 1.958e +7 |  | 2.698 |  |
| presentation order + session + standard duration + condition + presentation order  ✻  standard duration + presentation order  ✻  condition + standard duration  ✻  condition |  | 0.006 |  | 0.005 |  | 0.908 |  | 1.949e +7 |  | 3.385 |  |
| presentation order + session + standard duration + condition + session  ✻  standard duration + presentation order  ✻  condition + standard duration  ✻  condition |  | 0.006 |  | 0.005 |  | 0.829 |  | 1.780e +7 |  | 5.178 |  |
| presentation order + session + standard duration + condition + presentation order  ✻  session + presentation order  ✻  condition + session  ✻  condition |  | 0.006 |  | 0.005 |  | 0.803 |  | 1.724e +7 |  | 8.048 |  |
| presentation order + session + standard duration + condition + standard duration  ✻  condition |  | 0.006 |  | 0.005 |  | 0.796 |  | 1.710e +7 |  | 3.070 |  |
| presentation order + session + standard duration + condition + presentation order  ✻  standard duration + presentation order  ✻  condition + session  ✻  condition |  | 0.006 |  | 0.004 |  | 0.728 |  | 1.564e +7 |  | 3.780 |  |
| presentation order + session + standard duration + condition + session  ✻  condition |  | 0.006 |  | 0.004 |  | 0.674 |  | 1.448e +7 |  | 2.970 |  |
| presentation order + session + standard duration + condition + session  ✻  standard duration + presentation order  ✻  condition + session  ✻  condition |  | 0.006 |  | 0.004 |  | 0.657 |  | 1.412e +7 |  | 4.259 |  |
| presentation order + session + standard duration + condition + presentation order  ✻  session + presentation order  ✻  standard duration + presentation order  ✻  condition |  | 0.006 |  | 0.004 |  | 0.648 |  | 1.393e +7 |  | 5.012 |  |
| presentation order + condition |  | 0.006 |  | 0.004 |  | 0.592 |  | 1.272e +7 |  | 2.792 |  |
| presentation order + session |  | 0.006 |  | 0.003 |  | 0.512 |  | 1.102e +7 |  | 40.835 |  |
| presentation order + session + standard duration + condition + presentation order  ✻  session |  | 0.006 |  | 0.003 |  | 0.484 |  | 1.042e +7 |  | 7.136 |  |
| presentation order + session + standard duration + condition + presentation order  ✻  session + session  ✻  standard duration + presentation order  ✻  condition |  | 0.006 |  | 0.003 |  | 0.462 |  | 9.951e +6 |  | 6.682 |  |
| presentation order + session + standard duration + condition + presentation order  ✻  standard duration + session  ✻  standard duration + presentation order  ✻  condition + session  ✻  condition + standard duration  ✻  condition |  | 0.006 |  | 0.003 |  | 0.461 |  | 9.921e +6 |  | 63.525 |  |
| presentation order + session + standard duration + condition + presentation order  ✻  standard duration + session  ✻  standard duration + presentation order  ✻  condition |  | 0.006 |  | 0.003 |  | 0.461 |  | 9.915e +6 |  | 5.737 |  |
| presentation order + session + standard duration + condition + presentation order  ✻  standard duration + presentation order  ✻  condition + session  ✻  condition + standard duration  ✻  condition |  | 0.006 |  | 0.003 |  | 0.430 |  | 9.254e +6 |  | 5.504 |  |
| presentation order + session + standard duration + condition + presentation order  ✻  session + presentation order  ✻  condition + session  ✻  condition + standard duration  ✻  condition |  | 0.006 |  | 0.003 |  | 0.420 |  | 9.039e +6 |  | 4.949 |  |
| presentation order + session + standard duration + condition + session  ✻  standard duration |  | 0.006 |  | 0.002 |  | 0.363 |  | 7.817e +6 |  | 2.260 |  |
| presentation order + session + standard duration + condition + session  ✻  standard duration + presentation order  ✻  condition + session  ✻  condition + standard duration  ✻  condition |  | 0.006 |  | 0.002 |  | 0.356 |  | 7.668e +6 |  | 9.156 |  |
| presentation order + session + condition + presentation order  ✻  condition |  | 0.006 |  | 0.002 |  | 0.352 |  | 7.572e +6 |  | 2.519 |  |
| presentation order + session + standard duration + condition + session  ✻  condition + standard duration  ✻  condition |  | 0.006 |  | 0.002 |  | 0.343 |  | 7.379e +6 |  | 4.622 |  |
| presentation order + session + standard duration + presentation order  ✻  session + presentation order  ✻  standard duration |  | 0.006 |  | 0.002 |  | 0.330 |  | 7.101e +6 |  | 5.062 |  |
| presentation order + session + standard duration + condition + presentation order  ✻  session + presentation order  ✻  condition + session  ✻  condition + presentation order  ✻  session  ✻  condition |  | 0.006 |  | 0.002 |  | 0.327 |  | 7.034e +6 |  | 3.470 |  |
| presentation order + session + standard duration + presentation order  ✻  standard duration + session  ✻  standard duration |  | 0.006 |  | 0.002 |  | 0.304 |  | 6.550e +6 |  | 8.285 |  |
| presentation order + session + standard duration + condition + presentation order  ✻  standard duration + presentation order  ✻  condition + standard duration  ✻  condition + presentation order  ✻  standard duration  ✻  condition |  | 0.006 |  | 0.002 |  | 0.300 |  | 6.464e +6 |  | 3.403 |  |
| presentation order + session + standard duration + presentation order  ✻  session + session  ✻  standard duration |  | 0.006 |  | 0.002 |  | 0.278 |  | 5.980e +6 |  | 4.614 |  |
| presentation order + session + standard duration + condition + presentation order  ✻  session + presentation order  ✻  standard duration + presentation order  ✻  condition + standard duration  ✻  condition |  | 0.006 |  | 0.002 |  | 0.263 |  | 5.676e +6 |  | 4.350 |  |
| presentation order + session + standard duration + condition + presentation order  ✻  session + standard duration  ✻  condition |  | 0.006 |  | 0.001 |  | 0.242 |  | 5.215e +6 |  | 3.913 |  |
| presentation order + session + standard duration + condition + presentation order  ✻  standard duration + session  ✻  standard duration + presentation order  ✻  condition + standard duration  ✻  condition |  | 0.006 |  | 0.001 |  | 0.237 |  | 5.105e +6 |  | 4.946 |  |
| presentation order + session + standard duration + condition + presentation order  ✻  session + presentation order  ✻  condition + session  ✻  condition + standard duration  ✻  condition + presentation order  ✻  session  ✻  condition |  | 0.006 |  | 0.001 |  | 0.228 |  | 4.916e +6 |  | 12.839 |  |
| presentation order + session + standard duration + condition + presentation order  ✻  session + session  ✻  standard duration + presentation order  ✻  condition + standard duration  ✻  condition |  | 0.006 |  | 0.001 |  | 0.226 |  | 4.881e +6 |  | 5.487 |  |
| presentation order + session + standard duration + condition + presentation order  ✻  standard duration + standard duration  ✻  condition |  | 0.006 |  | 0.001 |  | 0.224 |  | 4.826e +6 |  | 2.890 |  |
| presentation order + session + standard duration + condition + presentation order  ✻  session + presentation order  ✻  standard duration + presentation order  ✻  condition + session  ✻  condition |  | 0.006 |  | 0.001 |  | 0.220 |  | 4.740e +6 |  | 5.141 |  |
| presentation order + session + standard duration + condition + presentation order  ✻  standard duration + session  ✻  standard duration + presentation order  ✻  condition + session  ✻  condition |  | 0.006 |  | 0.001 |  | 0.219 |  | 4.710e +6 |  | 5.310 |  |
| presentation order + session + standard duration + condition + presentation order  ✻  session + session  ✻  standard duration + presentation order  ✻  condition + session  ✻  condition |  | 0.006 |  | 0.001 |  | 0.207 |  | 4.459e +6 |  | 15.774 |  |
| presentation order + session + standard duration + condition + session  ✻  standard duration + standard duration  ✻  condition |  | 0.006 |  | 0.001 |  | 0.202 |  | 4.360e +6 |  | 3.863 |  |
| presentation order + session + standard duration + condition + presentation order  ✻  standard duration + session  ✻  condition |  | 0.006 |  | 0.001 |  | 0.192 |  | 4.148e +6 |  | 3.989 |  |
| presentation order + session + standard duration + condition + presentation order  ✻  session + session  ✻  condition |  | 0.006 |  | 0.001 |  | 0.186 |  | 4.013e +6 |  | 2.809 |  |
| presentation order + session + standard duration + condition + session  ✻  standard duration + session  ✻  condition |  | 0.006 |  | 9.256e  -4 |  | 0.154 |  | 3.316e +6 |  | 3.084 |  |
| presentation order + session + condition + presentation order  ✻  condition + session  ✻  condition |  | 0.006 |  | 8.873e  -4 |  | 0.147 |  | 3.179e +6 |  | 3.598 |  |
| presentation order + session + standard duration + condition + presentation order  ✻  session + presentation order  ✻  standard duration + session  ✻  standard duration + presentation order  ✻  condition |  | 0.006 |  | 8.787e  -4 |  | 0.146 |  | 3.148e +6 |  | 14.425 |  |
| presentation order + session + standard duration + condition + presentation order  ✻  session + presentation order  ✻  standard duration |  | 0.006 |  | 8.125e  -4 |  | 0.135 |  | 2.910e +6 |  | 4.371 |  |
| presentation order + session + standard duration + condition + presentation order  ✻  standard duration + presentation order  ✻  condition + session  ✻  condition + standard duration  ✻  condition + presentation order  ✻  standard duration  ✻  condition |  | 0.006 |  | 7.862e  -4 |  | 0.131 |  | 2.816e +6 |  | 5.552 |  |
| presentation order + session + standard duration + condition + presentation order  ✻  session + presentation order  ✻  standard duration + presentation order  ✻  condition + session  ✻  condition + standard duration  ✻  condition |  | 0.006 |  | 7.727e  -4 |  | 0.128 |  | 2.768e +6 |  | 11.597 |  |
| presentation order + session + condition |  | 0.006 |  | 7.056e  -4 |  | 0.117 |  | 2.528e +6 |  | 3.066 |  |
| presentation order + session + standard duration + condition + presentation order  ✻  standard duration + session  ✻  standard duration |  | 0.006 |  | 6.856e  -4 |  | 0.114 |  | 2.456e +6 |  | 3.773 |  |
| presentation order + session + standard duration + condition + session  ✻  standard duration + presentation order  ✻  condition + session  ✻  condition + standard duration  ✻  condition + session  ✻  standard duration  ✻  condition |  | 0.006 |  | 6.511e  -4 |  | 0.108 |  | 2.332e +6 |  | 6.391 |  |
| presentation order + session + condition + presentation order  ✻  session + presentation order  ✻  condition |  | 0.006 |  | 6.377e  -4 |  | 0.106 |  | 2.284e +6 |  | 4.620 |  |
| presentation order + session + standard duration + condition + presentation order  ✻  session + session  ✻  standard duration |  | 0.006 |  | 6.228e  -4 |  | 0.103 |  | 2.231e +6 |  | 2.874 |  |
| presentation order + session + standard duration + condition + presentation order  ✻  session + session  ✻  standard duration + presentation order  ✻  condition + session  ✻  condition + standard duration  ✻  condition |  | 0.006 |  | 5.960e  -4 |  | 0.099 |  | 2.135e +6 |  | 4.767 |  |
| presentation order + session + standard duration + condition + presentation order  ✻  standard duration + session  ✻  condition + standard duration  ✻  condition |  | 0.006 |  | 5.940e  -4 |  | 0.099 |  | 2.128e +6 |  | 4.016 |  |
| presentation order + session + standard duration + condition + presentation order  ✻  standard duration + session  ✻  standard duration + presentation order  ✻  condition + standard duration  ✻  condition + presentation order  ✻  standard duration  ✻  condition |  | 0.006 |  | 5.910e  -4 |  | 0.098 |  | 2.117e +6 |  | 6.528 |  |
| presentation order + session + standard duration + condition + session  ✻  standard duration + session  ✻  condition + standard duration  ✻  condition |  | 0.006 |  | 5.849e  -4 |  | 0.097 |  | 2.095e +6 |  | 5.660 |  |
| presentation order + session + standard duration + condition + presentation order  ✻  session + session  ✻  condition + standard duration  ✻  condition |  | 0.006 |  | 5.837e  -4 |  | 0.097 |  | 2.091e +6 |  | 3.514 |  |
| presentation order + session + standard duration + condition + presentation order  ✻  session + presentation order  ✻  standard duration + presentation order  ✻  condition + session  ✻  condition + presentation order  ✻  session  ✻  condition |  | 0.006 |  | 5.688e  -4 |  | 0.094 |  | 2.038e +6 |  | 4.902 |  |
| presentation order + session + standard duration + presentation order  ✻  session + presentation order  ✻  standard duration + session  ✻  standard duration |  | 0.006 |  | 5.370e  -4 |  | 0.089 |  | 1.924e +6 |  | 4.703 |  |
| presentation order + session + standard duration + condition + presentation order  ✻  session + presentation order  ✻  standard duration + presentation order  ✻  condition + standard duration  ✻  condition + presentation order  ✻  standard duration  ✻  condition |  | 0.006 |  | 5.323e  -4 |  | 0.088 |  | 1.907e +6 |  | 4.703 |  |
| presentation order + session + standard duration + condition + presentation order  ✻  session + session  ✻  standard duration + presentation order  ✻  condition + session  ✻  condition + presentation order  ✻  session  ✻  condition |  | 0.006 |  | 5.050e  -4 |  | 0.084 |  | 1.809e +6 |  | 12.692 |  |
| presentation order + session + presentation order  ✻  session |  | 0.006 |  | 4.887e  -4 |  | 0.081 |  | 1.751e +6 |  | 2.948 |  |
| presentation order + session + standard duration + condition + presentation order  ✻  session + presentation order  ✻  standard duration + session  ✻  standard duration + presentation order  ✻  condition + standard duration  ✻  condition |  | 0.006 |  | 4.645e  -4 |  | 0.077 |  | 1.664e +6 |  | 6.480 |  |
| presentation order + session + standard duration + condition + presentation order  ✻  standard duration + session  ✻  standard duration + standard duration  ✻  condition |  | 0.006 |  | 4.153e  -4 |  | 0.069 |  | 1.488e +6 |  | 13.481 |  |
| presentation order + session + standard duration + condition + presentation order  ✻  session + presentation order  ✻  standard duration + standard duration  ✻  condition |  | 0.006 |  | 4.012e  -4 |  | 0.067 |  | 1.437e +6 |  | 3.914 |  |
| presentation order + session + standard duration + condition + presentation order  ✻  session + presentation order  ✻  standard duration + session  ✻  condition |  | 0.006 |  | 3.595e  -4 |  | 0.060 |  | 1.288e +6 |  | 4.876 |  |
| presentation order + session + standard duration + condition + presentation order  ✻  session + presentation order  ✻  standard duration + presentation order  ✻  condition + session  ✻  condition + standard duration  ✻  condition + presentation order  ✻  session  ✻  condition |  | 0.006 |  | 3.364e  -4 |  | 0.056 |  | 1.205e +6 |  | 8.273 |  |
| presentation order + session + standard duration + condition + presentation order  ✻  session + session  ✻  standard duration + standard duration  ✻  condition |  | 0.006 |  | 3.226e  -4 |  | 0.054 |  | 1.156e +6 |  | 4.004 |  |
| presentation order + session + standard duration + condition + presentation order  ✻  session + presentation order  ✻  standard duration + session  ✻  standard duration + presentation order  ✻  condition + session  ✻  condition |  | 0.006 |  | 3.166e  -4 |  | 0.053 |  | 1.134e +6 |  | 4.354 |  |
| presentation order + session + standard duration + condition + presentation order  ✻  session + presentation order  ✻  standard duration + session  ✻  standard duration + presentation order  ✻  condition + presentation order  ✻  session  ✻  standard duration |  | 0.006 |  | 3.067e  -4 |  | 0.051 |  | 1.099e +6 |  | 7.472 |  |
| presentation order + session + standard duration + condition + presentation order  ✻  standard duration + session  ✻  standard duration + session  ✻  condition |  | 0.006 |  | 3.056e  -4 |  | 0.051 |  | 1.095e +6 |  | 9.748 |  |
| presentation order + session + condition + session  ✻  condition |  | 0.006 |  | 2.925e  -4 |  | 0.049 |  | 1.048e +6 |  | 8.343 |  |
| presentation order + session + standard duration + condition + presentation order  ✻  session + session  ✻  standard duration + session  ✻  condition |  | 0.006 |  | 2.819e  -4 |  | 0.047 |  | 1.010e +6 |  | 4.055 |  |
| presentation order + session + condition + presentation order  ✻  session + presentation order  ✻  condition + session  ✻  condition |  | 0.006 |  | 2.810e  -4 |  | 0.047 |  | 1.007e +6 |  | 4.269 |  |
| presentation order + session + standard duration + condition + presentation order  ✻  session + session  ✻  standard duration + presentation order  ✻  condition + session  ✻  condition + standard duration  ✻  condition + presentation order  ✻  session  ✻  condition |  | 0.006 |  | 2.606e  -4 |  | 0.043 |  | 933593.304 |  | 5.542 |  |
| presentation order + session + standard duration + condition + presentation order  ✻  session + presentation order  ✻  standard duration + presentation order  ✻  condition + session  ✻  condition + standard duration  ✻  condition + presentation order  ✻  standard duration  ✻  condition |  | 0.006 |  | 2.555e  -4 |  | 0.042 |  | 915238.953 |  | 5.025 |  |
| presentation order + session + standard duration + condition + presentation order  ✻  standard duration + session  ✻  standard duration + presentation order  ✻  condition + session  ✻  condition + standard duration  ✻  condition + session  ✻  standard duration  ✻  condition |  | 0.006 |  | 2.122e  -4 |  | 0.035 |  | 760124.145 |  | 6.502 |  |
| presentation order + session + standard duration + condition + presentation order  ✻  session + session  ✻  standard duration + presentation order  ✻  condition + session  ✻  condition + standard duration  ✻  condition + session  ✻  standard duration  ✻  condition |  | 0.006 |  | 2.112e  -4 |  | 0.035 |  | 756636.272 |  | 12.777 |  |
| presentation order + session + standard duration + condition + presentation order  ✻  standard duration + session  ✻  standard duration + presentation order  ✻  condition + session  ✻  condition + standard duration  ✻  condition + presentation order  ✻  standard duration  ✻  condition |  | 0.006 |  | 2.071e  -4 |  | 0.034 |  | 741776.265 |  | 5.449 |  |
| presentation order + session + standard duration + condition + presentation order  ✻  session + presentation order  ✻  standard duration + session  ✻  standard duration + presentation order  ✻  condition + session  ✻  condition + standard duration  ✻  condition |  | 0.006 |  | 1.993e  -4 |  | 0.033 |  | 714104.160 |  | 13.493 |  |
| presentation order + session + standard duration + condition + presentation order  ✻  session + presentation order  ✻  standard duration + session  ✻  standard duration |  | 0.006 |  | 1.923e  -4 |  | 0.032 |  | 688879.383 |  | 5.048 |  |
| presentation order + session + condition + presentation order  ✻  session |  | 0.006 |  | 1.899e  -4 |  | 0.032 |  | 680434.436 |  | 2.712 |  |
| presentation order + session + standard duration + presentation order  ✻  session + presentation order  ✻  standard duration + session  ✻  standard duration + presentation order  ✻  session  ✻  standard duration |  | 0.006 |  | 1.854e  -4 |  | 0.031 |  | 664287.441 |  | 9.808 |  |
| presentation order + session + standard duration + condition + presentation order  ✻  session + presentation order  ✻  standard duration + session  ✻  condition + standard duration  ✻  condition |  | 0.006 |  | 1.791e  -4 |  | 0.030 |  | 641494.661 |  | 4.112 |  |
| presentation order + session + standard duration + condition + session  ✻  standard duration + session  ✻  condition + standard duration  ✻  condition + session  ✻  standard duration  ✻  condition |  | 0.006 |  | 1.665e  -4 |  | 0.028 |  | 596456.699 |  | 6.542 |  |
| presentation order + session + standard duration + condition + presentation order  ✻  session + presentation order  ✻  standard duration + session  ✻  standard duration + presentation order  ✻  condition + session  ✻  condition + presentation order  ✻  session  ✻  condition |  | 0.006 |  | 1.602e  -4 |  | 0.027 |  | 574012.194 |  | 8.889 |  |
| presentation order + session + standard duration + condition + presentation order  ✻  session + presentation order  ✻  standard duration + session  ✻  standard duration + presentation order  ✻  condition + standard duration  ✻  condition + presentation order  ✻  session  ✻  standard duration |  | 0.006 |  | 1.583e  -4 |  | 0.026 |  | 567085.741 |  | 10.871 |  |
| presentation order + session + standard duration + condition + presentation order  ✻  standard duration + session  ✻  standard duration + session  ✻  condition + standard duration  ✻  condition |  | 0.006 |  | 1.516e  -4 |  | 0.025 |  | 543184.180 |  | 6.143 |  |
| presentation order + session + standard duration + condition + presentation order  ✻  session + session  ✻  standard duration + session  ✻  condition + standard duration  ✻  condition |  | 0.006 |  | 1.472e  -4 |  | 0.024 |  | 527324.482 |  | 4.007 |  |
| presentation order + session + standard duration + condition + presentation order  ✻  session + presentation order  ✻  standard duration + presentation order  ✻  condition + session  ✻  condition + standard duration  ✻  condition + presentation order  ✻  session  ✻  condition + presentation order  ✻  standard duration  ✻  condition |  | 0.006 |  | 1.432e  -4 |  | 0.024 |  | 512836.114 |  | 12.658 |  |
| presentation order + session + condition + presentation order  ✻  session + presentation order  ✻  condition + session  ✻  condition + presentation order  ✻  session  ✻  condition |  | 0.006 |  | 1.343e  -4 |  | 0.022 |  | 481114.973 |  | 5.741 |  |
| presentation order + session + standard duration + condition + presentation order  ✻  session + presentation order  ✻  standard duration + session  ✻  standard duration + presentation order  ✻  condition + standard duration  ✻  condition + presentation order  ✻  standard duration  ✻  condition |  | 0.006 |  | 1.337e  -4 |  | 0.022 |  | 479087.399 |  | 7.218 |  |
| presentation order + session + standard duration + condition + presentation order  ✻  session + presentation order  ✻  standard duration + session  ✻  standard duration + presentation order  ✻  condition + session  ✻  condition + presentation order  ✻  session  ✻  standard duration |  | 0.006 |  | 1.215e  -4 |  | 0.020 |  | 435381.909 |  | 5.643 |  |
| presentation order + session + standard duration + condition + presentation order  ✻  session + presentation order  ✻  standard duration + session  ✻  standard duration + standard duration  ✻  condition |  | 0.006 |  | 1.013e  -4 |  | 0.017 |  | 362918.739 |  | 5.725 |  |
| presentation order + session + condition + presentation order  ✻  session + session  ✻  condition |  | 0.006 |  | 8.695e  -5 |  | 0.014 |  | 311494.834 |  | 4.542 |  |
| presentation order + session + standard duration + condition + presentation order  ✻  session + session  ✻  standard duration + presentation order  ✻  condition + session  ✻  condition + standard duration  ✻  condition + presentation order  ✻  session  ✻  condition + session  ✻  standard duration  ✻  condition |  | 0.006 |  | 8.635e  -5 |  | 0.014 |  | 309323.208 |  | 9.331 |  |
| presentation order + session + standard duration + condition + presentation order  ✻  session + presentation order  ✻  standard duration + session  ✻  standard duration + session  ✻  condition |  | 0.006 |  | 8.304e  -5 |  | 0.014 |  | 297489.625 |  | 7.213 |  |
| presentation order + session + standard duration + condition + presentation order  ✻  standard duration + session  ✻  standard duration + presentation order  ✻  condition + session  ✻  condition + standard duration  ✻  condition + presentation order  ✻  standard duration  ✻  condition + session  ✻  standard duration  ✻  condition |  | 0.006 |  | 8.170e  -5 |  | 0.014 |  | 292666.153 |  | 7.673 |  |
| presentation order + session + standard duration + condition + presentation order  ✻  session + presentation order  ✻  standard duration + session  ✻  standard duration + presentation order  ✻  condition + session  ✻  condition + standard duration  ✻  condition + presentation order  ✻  session  ✻  condition |  | 0.006 |  | 7.492e  -5 |  | 0.012 |  | 268368.653 |  | 6.548 |  |
| presentation order + session + standard duration + condition + presentation order  ✻  session + presentation order  ✻  standard duration + session  ✻  standard duration + presentation order  ✻  condition + session  ✻  condition + standard duration  ✻  condition + presentation order  ✻  session  ✻  standard duration |  | 0.006 |  | 7.348e  -5 |  | 0.012 |  | 263228.492 |  | 8.976 |  |
| presentation order + session + standard duration + condition + presentation order  ✻  session + presentation order  ✻  standard duration + session  ✻  standard duration + presentation order  ✻  session  ✻  standard duration |  | 0.006 |  | 6.963e  -5 |  | 0.012 |  | 249419.071 |  | 4.412 |  |
| presentation order + session + standard duration + condition + presentation order  ✻  session + presentation order  ✻  standard duration + session  ✻  standard duration + presentation order  ✻  condition + session  ✻  condition + standard duration  ✻  condition + session  ✻  standard duration  ✻  condition |  | 0.006 |  | 6.256e  -5 |  | 0.010 |  | 224121.756 |  | 6.969 |  |
| presentation order + session + standard duration + condition + presentation order  ✻  session + presentation order  ✻  standard duration + session  ✻  standard duration + presentation order  ✻  condition + session  ✻  condition + standard duration  ✻  condition + presentation order  ✻  standard duration  ✻  condition |  | 0.006 |  | 5.832e  -5 |  | 0.010 |  | 208918.086 |  | 5.779 |  |
| presentation order + session + standard duration + condition + presentation order  ✻  standard duration + session  ✻  standard duration + session  ✻  condition + standard duration  ✻  condition + session  ✻  standard duration  ✻  condition |  | 0.006 |  | 5.404e  -5 |  | 0.009 |  | 193579.579 |  | 6.687 |  |
| presentation order + session + standard duration + condition + presentation order  ✻  session + presentation order  ✻  standard duration + session  ✻  standard duration + presentation order  ✻  condition + standard duration  ✻  condition + presentation order  ✻  session  ✻  standard duration + presentation order  ✻  standard duration  ✻  condition |  | 0.006 |  | 5.349e  -5 |  | 0.009 |  | 191616.082 |  | 9.785 |  |
| presentation order + session + standard duration + condition + presentation order  ✻  session + presentation order  ✻  standard duration + session  ✻  standard duration + presentation order  ✻  condition + session  ✻  condition + presentation order  ✻  session  ✻  standard duration + presentation order  ✻  session  ✻  condition |  | 0.006 |  | 5.227e  -5 |  | 0.009 |  | 187248.430 |  | 5.147 |  |
| presentation order + session + standard duration + condition + presentation order  ✻  session + presentation order  ✻  standard duration + session  ✻  standard duration + session  ✻  condition + standard duration  ✻  condition |  | 0.006 |  | 4.585e  -5 |  | 0.008 |  | 164247.234 |  | 6.978 |  |
| presentation order + session + standard duration + condition + presentation order  ✻  session + session  ✻  standard duration + session  ✻  condition + standard duration  ✻  condition + session  ✻  standard duration  ✻  condition |  | 0.006 |  | 4.199e  -5 |  | 0.007 |  | 150433.464 |  | 4.282 |  |
| presentation order + session + standard duration + condition + presentation order  ✻  session + presentation order  ✻  standard duration + session  ✻  standard duration + standard duration  ✻  condition + presentation order  ✻  session  ✻  standard duration |  | 0.006 |  | 3.625e  -5 |  | 0.006 |  | 129858.716 |  | 5.168 |  |
| presentation order + session + standard duration + condition + presentation order  ✻  session + presentation order  ✻  standard duration + session  ✻  standard duration + presentation order  ✻  condition + session  ✻  condition + standard duration  ✻  condition + presentation order  ✻  session  ✻  standard duration + presentation order  ✻  session  ✻  condition |  | 0.006 |  | 3.274e  -5 |  | 0.005 |  | 117298.339 |  | 10.001 |  |
| presentation order + session + standard duration + condition + presentation order  ✻  session + presentation order  ✻  standard duration + session  ✻  standard duration + session  ✻  condition + presentation order  ✻  session  ✻  standard duration |  | 0.006 |  | 3.193e  -5 |  | 0.005 |  | 114375.225 |  | 6.497 |  |
| presentation order + session + standard duration + condition + presentation order  ✻  session + presentation order  ✻  standard duration + session  ✻  standard duration + presentation order  ✻  condition + session  ✻  condition + standard duration  ✻  condition + presentation order  ✻  session  ✻  condition + session  ✻  standard duration  ✻  condition |  | 0.006 |  | 3.025e  -5 |  | 0.005 |  | 108373.400 |  | 10.645 |  |
| presentation order + session + standard duration + condition + presentation order  ✻  session + presentation order  ✻  standard duration + session  ✻  standard duration + presentation order  ✻  condition + session  ✻  condition + standard duration  ✻  condition + presentation order  ✻  session  ✻  condition + presentation order  ✻  standard duration  ✻  condition |  | 0.006 |  | 2.823e  -5 |  | 0.005 |  | 101120.525 |  | 8.549 |  |
| presentation order + session + standard duration + condition + presentation order  ✻  session + presentation order  ✻  standard duration + session  ✻  standard duration + presentation order  ✻  condition + session  ✻  condition + standard duration  ✻  condition + presentation order  ✻  session  ✻  standard duration + session  ✻  standard duration  ✻  condition |  | 0.006 |  | 2.131e  -5 |  | 0.004 |  | 76330.753 |  | 7.636 |  |
| presentation order + session + standard duration + condition + presentation order  ✻  session + presentation order  ✻  standard duration + session  ✻  standard duration + presentation order  ✻  condition + session  ✻  condition + standard duration  ✻  condition + presentation order  ✻  session  ✻  standard duration + presentation order  ✻  standard duration  ✻  condition |  | 0.006 |  | 2.122e  -5 |  | 0.004 |  | 76021.820 |  | 6.232 |  |
| presentation order + session + standard duration + condition + presentation order  ✻  session + presentation order  ✻  standard duration + session  ✻  standard duration + presentation order  ✻  condition + session  ✻  condition + standard duration  ✻  condition + presentation order  ✻  standard duration  ✻  condition + session  ✻  standard duration  ✻  condition |  | 0.006 |  | 1.826e  -5 |  | 0.003 |  | 65400.985 |  | 7.039 |  |
| presentation order + session + standard duration + condition + presentation order  ✻  session + presentation order  ✻  standard duration + session  ✻  standard duration + session  ✻  condition + standard duration  ✻  condition + session  ✻  standard duration  ✻  condition |  | 0.006 |  | 1.794e  -5 |  | 0.003 |  | 64256.113 |  | 29.721 |  |
| presentation order + session + standard duration + condition + presentation order  ✻  session + presentation order  ✻  standard duration + session  ✻  standard duration + session  ✻  condition + standard duration  ✻  condition + presentation order  ✻  session  ✻  standard duration |  | 0.006 |  | 1.629e  -5 |  | 0.003 |  | 58345.026 |  | 5.662 |  |
| presentation order + session + standard duration + condition + presentation order  ✻  session + presentation order  ✻  standard duration + session  ✻  standard duration + presentation order  ✻  condition + session  ✻  condition + standard duration  ✻  condition + presentation order  ✻  session  ✻  standard duration + presentation order  ✻  session  ✻  condition + session  ✻  standard duration  ✻  condition |  | 0.006 |  | 9.784e  -6 |  | 0.002 |  | 35050.617 |  | 9.548 |  |
| presentation order + session + standard duration + condition + presentation order  ✻  session + presentation order  ✻  standard duration + session  ✻  standard duration + presentation order  ✻  condition + session  ✻  condition + standard duration  ✻  condition + presentation order  ✻  session  ✻  standard duration + presentation order  ✻  session  ✻  condition + presentation order  ✻  standard duration  ✻  condition |  | 0.006 |  | 9.682e  -6 |  | 0.002 |  | 34684.754 |  | 6.788 |  |
| presentation order + session + standard duration + condition + presentation order  ✻  session + presentation order  ✻  standard duration + session  ✻  standard duration + presentation order  ✻  condition + session  ✻  condition + standard duration  ✻  condition + presentation order  ✻  session  ✻  condition + presentation order  ✻  standard duration  ✻  condition + session  ✻  standard duration  ✻  condition |  | 0.006 |  | 8.312e  -6 |  | 0.001 |  | 29776.575 |  | 7.906 |  |
| presentation order + session + standard duration + condition + presentation order  ✻  session + presentation order  ✻  standard duration + session  ✻  standard duration + presentation order  ✻  condition + session  ✻  condition + standard duration  ✻  condition + presentation order  ✻  session  ✻  standard duration + presentation order  ✻  standard duration  ✻  condition + session  ✻  standard duration  ✻  condition |  | 0.006 |  | 6.718e  -6 |  | 0.001 |  | 24066.428 |  | 8.285 |  |
| presentation order + session + standard duration + condition + presentation order  ✻  session + presentation order  ✻  standard duration + session  ✻  standard duration + session  ✻  condition + standard duration  ✻  condition + presentation order  ✻  session  ✻  standard duration + session  ✻  standard duration  ✻  condition |  | 0.006 |  | 4.816e  -6 |  | 7.995e  -4 |  | 17253.325 |  | 4.186 |  |
| presentation order + session + standard duration + condition + presentation order  ✻  session + presentation order  ✻  standard duration + session  ✻  standard duration + presentation order  ✻  condition + session  ✻  condition + standard duration  ✻  condition + presentation order  ✻  session  ✻  standard duration + presentation order  ✻  session  ✻  condition + presentation order  ✻  standard duration  ✻  condition + session  ✻  standard duration  ✻  condition |  | 0.006 |  | 4.239e  -6 |  | 7.037e  -4 |  | 15186.356 |  | 16.910 |  |
| presentation order + session + standard duration + condition + presentation order  ✻  session + presentation order  ✻  standard duration + session  ✻  standard duration + presentation order  ✻  condition + session  ✻  condition + standard duration  ✻  condition + presentation order  ✻  session  ✻  standard duration + presentation order  ✻  session  ✻  condition + presentation order  ✻  standard duration  ✻  condition + session  ✻  standard duration  ✻  condition + presentation order  ✻  session  ✻  standard duration  ✻  condition |  | 0.006 |  | 1.277e  -6 |  | 2.120e  -4 |  | 4574.030 |  | 9.598 |  |
| standard duration |  | 0.006 |  | 1.255e  -9 |  | 2.084e  -7 |  | 4.497 |  | 1.627 |  |
| standard duration + condition |  | 0.006 |  | 4.374e -10 |  | 7.261e  -8 |  | 1.567 |  | 2.729 |  |
| session + standard duration |  | 0.006 |  | 2.597e -10 |  | 4.311e  -8 |  | 0.930 |  | 4.728 |  |
| standard duration + condition + standard duration  ✻  condition |  | 0.006 |  | 1.770e -10 |  | 2.938e  -8 |  | 0.634 |  | 2.994 |  |
| condition |  | 0.006 |  | 8.988e -11 |  | 1.492e  -8 |  | 0.322 |  | 1.000 |  |
| session + standard duration + condition |  | 0.006 |  | 7.959e -11 |  | 1.321e  -8 |  | 0.285 |  | 2.048 |  |
| session + standard duration + session  ✻  standard duration |  | 0.006 |  | 6.225e -11 |  | 1.033e  -8 |  | 0.223 |  | 3.394 |  |
| session |  | 0.006 |  | 5.296e -11 |  | 8.791e  -9 |  | 0.190 |  | 1.723 |  |
| session + standard duration + condition + standard duration  ✻  condition |  | 0.006 |  | 4.056e -11 |  | 6.733e  -9 |  | 0.145 |  | 17.205 |  |
| session + standard duration + condition + session  ✻  condition |  | 0.006 |  | 3.019e -11 |  | 5.011e  -9 |  | 0.108 |  | 3.285 |  |
| session + standard duration + condition + session  ✻  standard duration |  | 0.006 |  | 2.260e -11 |  | 3.752e  -9 |  | 0.081 |  | 9.837 |  |
| session + condition |  | 0.006 |  | 1.738e -11 |  | 2.886e  -9 |  | 0.062 |  | 1.630 |  |
| session + standard duration + condition + session  ✻  condition + standard duration  ✻  condition |  | 0.006 |  | 1.338e -11 |  | 2.220e  -9 |  | 0.048 |  | 5.942 |  |
| session + standard duration + condition + session  ✻  standard duration + standard duration  ✻  condition |  | 0.006 |  | 7.916e -12 |  | 1.314e  -9 |  | 0.028 |  | 2.936 |  |
| session + standard duration + condition + session  ✻  standard duration + session  ✻  condition |  | 0.006 |  | 7.087e -12 |  | 1.176e  -9 |  | 0.025 |  | 3.320 |  |
| session + condition + session  ✻  condition |  | 0.006 |  | 6.589e -12 |  | 1.094e  -9 |  | 0.024 |  | 5.119 |  |
| session + standard duration + condition + session  ✻  standard duration + session  ✻  condition + standard duration  ✻  condition |  | 0.006 |  | 3.070e -12 |  | 5.097e -10 |  | 0.011 |  | 8.469 |  |
| session + standard duration + condition + session  ✻  standard duration + session  ✻  condition + standard duration  ✻  condition + session  ✻  standard duration  ✻  condition |  | 0.006 |  | 1.057e -12 |  | 1.754e -10 |  | 0.004 |  | 8.281 |  |
|  | | | | | | | | | | | |
| *Note.*  All models include subject | | | | | | | | | | | |

**Table 2** Model comparisons for Weber fractions in Experiment 1. P(M) represents the prior for each modal. P(M|data) represents how much the data were explained by the model. BF_M_ represents how much the data was likely under the alternative hypothesis than the null hypothesis. BF_10_ represents the Bayesian factor. Note that all models include subjects as a variable.

| **Model Comparison** | | | | | | | | | | | |
| --- | --- | --- | --- | --- | --- | --- | --- | --- | --- | --- | --- |
| **Models** | | **P(M)** | | **P(M\|data)** | | **BF _M_** | | **BF _10_** | | **error %** | |
| Null model (incl. subject) |  | 0.006 |  | 1.357e -16 |  | 2.252e -14 |  | 1.000 |  |  |  |
| presentation order + standard duration + condition + presentation order  ✻  standard duration + presentation order  ✻  condition |  | 0.006 |  | 0.442 |  | 131.342 |  | 3.256e +15 |  | 5.704 |  |
| presentation order + session + standard duration + condition + presentation order  ✻  standard duration + presentation order  ✻  condition |  | 0.006 |  | 0.127 |  | 24.074 |  | 9.336e +14 |  | 3.295 |  |
| presentation order + standard duration + condition + presentation order  ✻  standard duration + presentation order  ✻  condition + standard duration  ✻  condition |  | 0.006 |  | 0.118 |  | 22.142 |  | 8.675e +14 |  | 8.965 |  |
| presentation order + session + standard duration + condition + presentation order  ✻  standard duration + session  ✻  standard duration + presentation order  ✻  condition |  | 0.006 |  | 0.043 |  | 7.383 |  | 3.139e +14 |  | 4.039 |  |
| presentation order + session + standard duration + condition + presentation order  ✻  standard duration + presentation order  ✻  condition + session  ✻  condition |  | 0.006 |  | 0.040 |  | 6.897 |  | 2.940e +14 |  | 3.616 |  |
| presentation order + standard duration + condition + presentation order  ✻  standard duration + presentation order  ✻  condition + standard duration  ✻  condition + presentation order  ✻  standard duration  ✻  condition |  | 0.006 |  | 0.039 |  | 6.714 |  | 2.865e +14 |  | 10.938 |  |
| presentation order + session + standard duration + condition + presentation order  ✻  session + presentation order  ✻  standard duration + presentation order  ✻  condition |  | 0.006 |  | 0.033 |  | 5.605 |  | 2.407e +14 |  | 5.193 |  |
| presentation order + session + standard duration + condition + presentation order  ✻  standard duration + presentation order  ✻  condition + standard duration  ✻  condition |  | 0.006 |  | 0.030 |  | 5.214 |  | 2.244e +14 |  | 4.350 |  |
| presentation order + session + standard duration + condition + presentation order  ✻  standard duration + session  ✻  standard duration + presentation order  ✻  condition + session  ✻  condition |  | 0.006 |  | 0.017 |  | 2.817 |  | 1.230e +14 |  | 11.778 |  |
| presentation order + session + standard duration + condition + presentation order  ✻  standard duration + presentation order  ✻  condition + standard duration  ✻  condition + presentation order  ✻  standard duration  ✻  condition |  | 0.006 |  | 0.012 |  | 1.956 |  | 8.585e +13 |  | 10.305 |  |
| presentation order + session + standard duration + condition + presentation order  ✻  session + presentation order  ✻  standard duration + session  ✻  standard duration + presentation order  ✻  condition |  | 0.006 |  | 0.011 |  | 1.769 |  | 7.773e +13 |  | 10.831 |  |
| presentation order + session + standard duration + condition + presentation order  ✻  session + presentation order  ✻  standard duration + presentation order  ✻  condition + session  ✻  condition |  | 0.006 |  | 0.010 |  | 1.660 |  | 7.297e +13 |  | 5.949 |  |
| presentation order + session + standard duration + condition + presentation order  ✻  standard duration + session  ✻  standard duration + presentation order  ✻  condition + standard duration  ✻  condition |  | 0.006 |  | 0.010 |  | 1.610 |  | 7.080e +13 |  | 11.318 |  |
| presentation order + session + standard duration + condition + presentation order  ✻  standard duration + presentation order  ✻  condition + session  ✻  condition + standard duration  ✻  condition |  | 0.006 |  | 0.009 |  | 1.515 |  | 6.668e +13 |  | 10.509 |  |
| presentation order + session + standard duration + condition + presentation order  ✻  session + presentation order  ✻  standard duration + presentation order  ✻  condition + standard duration  ✻  condition |  | 0.006 |  | 0.008 |  | 1.311 |  | 5.777e +13 |  | 8.554 |  |
| presentation order + session + standard duration + condition + presentation order  ✻  session + presentation order  ✻  standard duration + session  ✻  standard duration + presentation order  ✻  condition + presentation order  ✻  session  ✻  standard duration |  | 0.006 |  | 0.005 |  | 0.835 |  | 3.688e +13 |  | 6.084 |  |
| presentation order + session + standard duration + condition + presentation order  ✻  standard duration + session  ✻  standard duration + presentation order  ✻  condition + standard duration  ✻  condition + presentation order  ✻  standard duration  ✻  condition |  | 0.006 |  | 0.004 |  | 0.657 |  | 2.905e +13 |  | 8.209 |  |
| presentation order + standard duration + condition + presentation order  ✻  standard duration |  | 0.006 |  | 0.004 |  | 0.634 |  | 2.803e +13 |  | 3.434 |  |
| presentation order + session + standard duration + condition + presentation order  ✻  standard duration + presentation order  ✻  condition + session  ✻  condition + standard duration  ✻  condition + presentation order  ✻  standard duration  ✻  condition |  | 0.006 |  | 0.004 |  | 0.612 |  | 2.707e +13 |  | 6.367 |  |
| presentation order + session + standard duration + condition + presentation order  ✻  standard duration + session  ✻  standard duration + presentation order  ✻  condition + session  ✻  condition + standard duration  ✻  condition |  | 0.006 |  | 0.004 |  | 0.608 |  | 2.692e +13 |  | 13.664 |  |
| presentation order + session + standard duration + condition + presentation order  ✻  session + presentation order  ✻  standard duration + presentation order  ✻  condition + session  ✻  condition + presentation order  ✻  session  ✻  condition |  | 0.006 |  | 0.004 |  | 0.598 |  | 2.646e +13 |  | 15.789 |  |
| presentation order + session + standard duration + condition + presentation order  ✻  session + presentation order  ✻  standard duration + session  ✻  standard duration + presentation order  ✻  condition + session  ✻  condition |  | 0.006 |  | 0.003 |  | 0.551 |  | 2.440e +13 |  | 5.608 |  |
| presentation order + session + standard duration + condition + presentation order  ✻  session + presentation order  ✻  standard duration + presentation order  ✻  condition + standard duration  ✻  condition + presentation order  ✻  standard duration  ✻  condition |  | 0.006 |  | 0.003 |  | 0.437 |  | 1.935e +13 |  | 6.105 |  |
| presentation order + session + standard duration + condition + presentation order  ✻  session + presentation order  ✻  standard duration + presentation order  ✻  condition + session  ✻  condition + standard duration  ✻  condition |  | 0.006 |  | 0.003 |  | 0.430 |  | 1.903e +13 |  | 9.163 |  |
| presentation order + session + standard duration + condition + presentation order  ✻  session + presentation order  ✻  standard duration + session  ✻  standard duration + presentation order  ✻  condition + standard duration  ✻  condition |  | 0.006 |  | 0.002 |  | 0.415 |  | 1.837e +13 |  | 7.292 |  |
| presentation order + session + standard duration + condition + presentation order  ✻  session + presentation order  ✻  standard duration + session  ✻  standard duration + presentation order  ✻  condition + session  ✻  condition + presentation order  ✻  session  ✻  standard duration |  | 0.006 |  | 0.002 |  | 0.315 |  | 1.397e +13 |  | 8.543 |  |
| presentation order + session + standard duration + condition + presentation order  ✻  standard duration + session  ✻  standard duration + presentation order  ✻  condition + session  ✻  condition + standard duration  ✻  condition + presentation order  ✻  standard duration  ✻  condition |  | 0.006 |  | 0.002 |  | 0.289 |  | 1.281e +13 |  | 17.177 |  |
| presentation order + session + standard duration + condition + presentation order  ✻  session + presentation order  ✻  standard duration + session  ✻  standard duration + presentation order  ✻  condition + standard duration  ✻  condition + presentation order  ✻  session  ✻  standard duration |  | 0.006 |  | 0.001 |  | 0.228 |  | 1.010e +13 |  | 15.561 |  |
| presentation order + session + standard duration + condition + presentation order  ✻  standard duration |  | 0.006 |  | 0.001 |  | 0.184 |  | 8.141e +12 |  | 3.217 |  |
| presentation order + session + standard duration + condition + presentation order  ✻  session + presentation order  ✻  standard duration + session  ✻  standard duration + presentation order  ✻  condition + session  ✻  condition + presentation order  ✻  session  ✻  condition |  | 0.006 |  | 0.001 |  | 0.176 |  | 7.810e +12 |  | 5.658 |  |
| presentation order + session + standard duration + condition + presentation order  ✻  standard duration + session  ✻  standard duration + presentation order  ✻  condition + session  ✻  condition + standard duration  ✻  condition + session  ✻  standard duration  ✻  condition |  | 0.006 |  | 0.001 |  | 0.173 |  | 7.662e +12 |  | 5.984 |  |
| presentation order + session + standard duration + condition + presentation order  ✻  session + presentation order  ✻  standard duration + presentation order  ✻  condition + session  ✻  condition + standard duration  ✻  condition + presentation order  ✻  standard duration  ✻  condition |  | 0.006 |  | 9.869e  -4 |  | 0.164 |  | 7.274e +12 |  | 14.527 |  |
| presentation order + standard duration + condition + presentation order  ✻  standard duration + standard duration  ✻  condition |  | 0.006 |  | 9.397e  -4 |  | 0.156 |  | 6.926e +12 |  | 4.948 |  |
| presentation order + session + standard duration + condition + presentation order  ✻  session + presentation order  ✻  standard duration + session  ✻  standard duration + presentation order  ✻  condition + standard duration  ✻  condition + presentation order  ✻  standard duration  ✻  condition |  | 0.006 |  | 8.699e  -4 |  | 0.145 |  | 6.412e +12 |  | 9.501 |  |
| presentation order + session + standard duration + condition + presentation order  ✻  session + presentation order  ✻  standard duration + session  ✻  standard duration + presentation order  ✻  condition + session  ✻  condition + standard duration  ✻  condition |  | 0.006 |  | 7.667e  -4 |  | 0.127 |  | 5.651e +12 |  | 5.010 |  |
| presentation order + session + standard duration + condition + presentation order  ✻  session + presentation order  ✻  standard duration + presentation order  ✻  condition + session  ✻  condition + standard duration  ✻  condition + presentation order  ✻  session  ✻  condition |  | 0.006 |  | 7.413e  -4 |  | 0.123 |  | 5.464e +12 |  | 5.680 |  |
| presentation order + standard duration + presentation order  ✻  standard duration |  | 0.006 |  | 6.889e  -4 |  | 0.114 |  | 5.078e +12 |  | 2.060 |  |
| presentation order + session + standard duration + condition + presentation order  ✻  session + presentation order  ✻  standard duration + session  ✻  standard duration + presentation order  ✻  condition + session  ✻  condition + presentation order  ✻  session  ✻  standard duration + presentation order  ✻  session  ✻  condition |  | 0.006 |  | 5.224e  -4 |  | 0.087 |  | 3.850e +12 |  | 5.590 |  |
| presentation order + session + standard duration + condition + presentation order  ✻  session + presentation order  ✻  standard duration + session  ✻  standard duration + presentation order  ✻  condition + session  ✻  condition + standard duration  ✻  condition + presentation order  ✻  session  ✻  standard duration |  | 0.006 |  | 4.962e  -4 |  | 0.082 |  | 3.657e +12 |  | 10.641 |  |
| presentation order + session + standard duration + condition + presentation order  ✻  session + presentation order  ✻  standard duration + session  ✻  standard duration + presentation order  ✻  condition + standard duration  ✻  condition + presentation order  ✻  session  ✻  standard duration + presentation order  ✻  standard duration  ✻  condition |  | 0.006 |  | 4.331e  -4 |  | 0.072 |  | 3.192e +12 |  | 6.294 |  |
| presentation order + session + standard duration + condition + presentation order  ✻  standard duration + session  ✻  standard duration + presentation order  ✻  condition + session  ✻  condition + standard duration  ✻  condition + presentation order  ✻  standard duration  ✻  condition + session  ✻  standard duration  ✻  condition |  | 0.006 |  | 4.111e  -4 |  | 0.068 |  | 3.030e +12 |  | 9.654 |  |
| presentation order + session + standard duration + condition + presentation order  ✻  standard duration + session  ✻  condition |  | 0.006 |  | 3.446e  -4 |  | 0.057 |  | 2.540e +12 |  | 5.335 |  |
| presentation order + session + standard duration + condition + presentation order  ✻  standard duration + session  ✻  standard duration |  | 0.006 |  | 3.380e  -4 |  | 0.056 |  | 2.491e +12 |  | 2.760 |  |
| presentation order + session + standard duration + condition + presentation order  ✻  session + presentation order  ✻  standard duration + session  ✻  standard duration + presentation order  ✻  condition + session  ✻  condition + standard duration  ✻  condition + session  ✻  standard duration  ✻  condition |  | 0.006 |  | 3.138e  -4 |  | 0.052 |  | 2.313e +12 |  | 17.830 |  |
| presentation order + session + standard duration + condition + presentation order  ✻  standard duration + standard duration  ✻  condition |  | 0.006 |  | 2.828e  -4 |  | 0.047 |  | 2.085e +12 |  | 5.138 |  |
| presentation order + session + standard duration + condition + presentation order  ✻  session + presentation order  ✻  standard duration |  | 0.006 |  | 2.791e  -4 |  | 0.046 |  | 2.057e +12 |  | 6.111 |  |
| presentation order + session + standard duration + condition + presentation order  ✻  session + presentation order  ✻  standard duration + session  ✻  standard duration + presentation order  ✻  condition + session  ✻  condition + standard duration  ✻  condition + presentation order  ✻  standard duration  ✻  condition |  | 0.006 |  | 2.577e  -4 |  | 0.043 |  | 1.899e +12 |  | 5.787 |  |
| presentation order + session + standard duration + condition + presentation order  ✻  session + presentation order  ✻  standard duration + presentation order  ✻  condition + session  ✻  condition + standard duration  ✻  condition + presentation order  ✻  session  ✻  condition + presentation order  ✻  standard duration  ✻  condition |  | 0.006 |  | 2.549e  -4 |  | 0.042 |  | 1.879e +12 |  | 6.846 |  |
| presentation order + session + standard duration + condition + presentation order  ✻  session + presentation order  ✻  standard duration + session  ✻  standard duration + presentation order  ✻  condition + session  ✻  condition + standard duration  ✻  condition + presentation order  ✻  session  ✻  condition |  | 0.006 |  | 2.450e  -4 |  | 0.041 |  | 1.806e +12 |  | 5.715 |  |
| presentation order + session + standard duration + condition + presentation order  ✻  session + presentation order  ✻  standard duration + session  ✻  standard duration + presentation order  ✻  condition + session  ✻  condition + standard duration  ✻  condition + presentation order  ✻  session  ✻  standard duration + presentation order  ✻  session  ✻  condition |  | 0.006 |  | 2.305e  -4 |  | 0.038 |  | 1.699e +12 |  | 36.707 |  |
| presentation order + session + standard duration + presentation order  ✻  standard duration |  | 0.006 |  | 2.296e  -4 |  | 0.038 |  | 1.693e +12 |  | 12.245 |  |
| presentation order + session + standard duration + condition + presentation order  ✻  session + presentation order  ✻  standard duration + session  ✻  standard duration + presentation order  ✻  condition + session  ✻  condition + standard duration  ✻  condition + presentation order  ✻  session  ✻  standard duration + presentation order  ✻  standard duration  ✻  condition |  | 0.006 |  | 1.338e  -4 |  | 0.022 |  | 9.860e +11 |  | 5.180 |  |
| presentation order + session + standard duration + condition + presentation order  ✻  session + presentation order  ✻  standard duration + session  ✻  standard duration + presentation order  ✻  condition + session  ✻  condition + standard duration  ✻  condition + presentation order  ✻  session  ✻  standard duration + session  ✻  standard duration  ✻  condition |  | 0.006 |  | 1.301e  -4 |  | 0.022 |  | 9.591e +11 |  | 8.103 |  |
| presentation order + session + standard duration + condition + presentation order  ✻  session + presentation order  ✻  standard duration + session  ✻  standard duration + session  ✻  condition + standard duration  ✻  condition + presentation order  ✻  session  ✻  standard duration |  | 0.006 |  | 1.189e  -4 |  | 0.020 |  | 8.767e +11 |  | 97.655 |  |
| presentation order + session + standard duration + condition + presentation order  ✻  standard duration + session  ✻  standard duration + session  ✻  condition |  | 0.006 |  | 1.185e  -4 |  | 0.020 |  | 8.736e +11 |  | 11.888 |  |
| presentation order + session + standard duration + condition + presentation order  ✻  session + presentation order  ✻  standard duration + session  ✻  standard duration + presentation order  ✻  condition + session  ✻  condition + standard duration  ✻  condition + presentation order  ✻  session  ✻  condition + presentation order  ✻  standard duration  ✻  condition |  | 0.006 |  | 9.960e  -5 |  | 0.017 |  | 7.341e +11 |  | 17.717 |  |
| presentation order + session + standard duration + condition + presentation order  ✻  session + presentation order  ✻  standard duration + session  ✻  standard duration + presentation order  ✻  condition + session  ✻  condition + standard duration  ✻  condition + presentation order  ✻  standard duration  ✻  condition + session  ✻  standard duration  ✻  condition |  | 0.006 |  | 8.838e  -5 |  | 0.015 |  | 6.514e +11 |  | 10.983 |  |
| presentation order + session + standard duration + condition + presentation order  ✻  standard duration + session  ✻  standard duration + standard duration  ✻  condition |  | 0.006 |  | 8.762e  -5 |  | 0.015 |  | 6.458e +11 |  | 10.167 |  |
| presentation order + session + standard duration + condition + presentation order  ✻  session + presentation order  ✻  standard duration + session  ✻  condition |  | 0.006 |  | 8.499e  -5 |  | 0.014 |  | 6.264e +11 |  | 7.245 |  |
| presentation order + session + standard duration + condition + presentation order  ✻  session + presentation order  ✻  standard duration + session  ✻  standard duration |  | 0.006 |  | 7.941e  -5 |  | 0.013 |  | 5.853e +11 |  | 4.310 |  |
| presentation order + session + standard duration + condition + presentation order  ✻  standard duration + session  ✻  condition + standard duration  ✻  condition |  | 0.006 |  | 7.939e  -5 |  | 0.013 |  | 5.852e +11 |  | 4.007 |  |
| presentation order + session + standard duration + condition + presentation order  ✻  session + presentation order  ✻  standard duration + session  ✻  standard duration + presentation order  ✻  condition + session  ✻  condition + standard duration  ✻  condition + presentation order  ✻  session  ✻  condition + session  ✻  standard duration  ✻  condition |  | 0.006 |  | 7.606e  -5 |  | 0.013 |  | 5.606e +11 |  | 8.749 |  |
| presentation order + session + standard duration + condition + presentation order  ✻  session + presentation order  ✻  standard duration + standard duration  ✻  condition |  | 0.006 |  | 7.603e  -5 |  | 0.013 |  | 5.604e +11 |  | 22.548 |  |
| presentation order + session + standard duration + presentation order  ✻  standard duration + session  ✻  standard duration |  | 0.006 |  | 7.190e  -5 |  | 0.012 |  | 5.300e +11 |  | 17.593 |  |
| presentation order + session + standard duration + condition + presentation order  ✻  session + presentation order  ✻  standard duration + session  ✻  standard duration + presentation order  ✻  session  ✻  standard duration |  | 0.006 |  | 6.050e  -5 |  | 0.010 |  | 4.459e +11 |  | 20.886 |  |
| presentation order + session + standard duration + condition + presentation order  ✻  session + presentation order  ✻  standard duration + session  ✻  standard duration + presentation order  ✻  condition + session  ✻  condition + standard duration  ✻  condition + presentation order  ✻  session  ✻  standard duration + presentation order  ✻  session  ✻  condition + session  ✻  standard duration  ✻  condition |  | 0.006 |  | 5.092e  -5 |  | 0.008 |  | 3.753e +11 |  | 21.990 |  |
| presentation order + session + standard duration + condition + presentation order  ✻  session + presentation order  ✻  standard duration + session  ✻  standard duration + presentation order  ✻  condition + session  ✻  condition + standard duration  ✻  condition + presentation order  ✻  session  ✻  standard duration + presentation order  ✻  session  ✻  condition + presentation order  ✻  standard duration  ✻  condition |  | 0.006 |  | 4.417e  -5 |  | 0.007 |  | 3.256e +11 |  | 8.100 |  |
| presentation order + session + standard duration + presentation order  ✻  session + presentation order  ✻  standard duration |  | 0.006 |  | 4.292e  -5 |  | 0.007 |  | 3.164e +11 |  | 2.272 |  |
| presentation order + session + standard duration + condition + presentation order  ✻  session + presentation order  ✻  standard duration + session  ✻  standard duration + presentation order  ✻  condition + session  ✻  condition + standard duration  ✻  condition + presentation order  ✻  session  ✻  standard duration + presentation order  ✻  standard duration  ✻  condition + session  ✻  standard duration  ✻  condition |  | 0.006 |  | 3.890e  -5 |  | 0.006 |  | 2.867e +11 |  | 4.825 |  |
| presentation order + session + standard duration + condition + presentation order  ✻  session + presentation order  ✻  standard duration + session  ✻  standard duration + session  ✻  condition |  | 0.006 |  | 2.666e  -5 |  | 0.004 |  | 1.965e +11 |  | 5.591 |  |
| presentation order + session + standard duration + condition + presentation order  ✻  standard duration + session  ✻  standard duration + session  ✻  condition + standard duration  ✻  condition |  | 0.006 |  | 2.664e  -5 |  | 0.004 |  | 1.964e +11 |  | 5.852 |  |
| presentation order + session + standard duration + condition + presentation order  ✻  session + presentation order  ✻  standard duration + session  ✻  standard duration + presentation order  ✻  condition + session  ✻  condition + standard duration  ✻  condition + presentation order  ✻  session  ✻  condition + presentation order  ✻  standard duration  ✻  condition + session  ✻  standard duration  ✻  condition |  | 0.006 |  | 2.482e  -5 |  | 0.004 |  | 1.830e +11 |  | 7.422 |  |
| presentation order + session + standard duration + condition + presentation order  ✻  session + presentation order  ✻  standard duration + session  ✻  standard duration + standard duration  ✻  condition |  | 0.006 |  | 2.090e  -5 |  | 0.003 |  | 1.540e +11 |  | 7.534 |  |
| presentation order + session + standard duration + condition + presentation order  ✻  session + presentation order  ✻  standard duration + session  ✻  condition + standard duration  ✻  condition |  | 0.006 |  | 1.948e  -5 |  | 0.003 |  | 1.436e +11 |  | 4.384 |  |
| presentation order + session + standard duration + presentation order  ✻  session + presentation order  ✻  standard duration + session  ✻  standard duration |  | 0.006 |  | 1.522e  -5 |  | 0.003 |  | 1.122e +11 |  | 4.017 |  |
| presentation order + session + standard duration + condition + presentation order  ✻  session + presentation order  ✻  standard duration + session  ✻  standard duration + session  ✻  condition + presentation order  ✻  session  ✻  standard duration |  | 0.006 |  | 1.458e  -5 |  | 0.002 |  | 1.074e +11 |  | 13.445 |  |
| presentation order + session + standard duration + condition + presentation order  ✻  session + presentation order  ✻  standard duration + session  ✻  standard duration + presentation order  ✻  condition + session  ✻  condition + standard duration  ✻  condition + presentation order  ✻  session  ✻  standard duration + presentation order  ✻  session  ✻  condition + presentation order  ✻  standard duration  ✻  condition + session  ✻  standard duration  ✻  condition |  | 0.006 |  | 1.282e  -5 |  | 0.002 |  | 9.450e +10 |  | 6.695 |  |
| presentation order + session + standard duration + condition + presentation order  ✻  standard duration + session  ✻  standard duration + session  ✻  condition + standard duration  ✻  condition + session  ✻  standard duration  ✻  condition |  | 0.006 |  | 1.231e  -5 |  | 0.002 |  | 9.072e +10 |  | 25.851 |  |
| presentation order + session + standard duration + condition + presentation order  ✻  session + presentation order  ✻  standard duration + session  ✻  standard duration + presentation order  ✻  condition + session  ✻  condition + standard duration  ✻  condition + presentation order  ✻  session  ✻  standard duration + presentation order  ✻  session  ✻  condition + presentation order  ✻  standard duration  ✻  condition + session  ✻  standard duration  ✻  condition + presentation order  ✻  session  ✻  standard duration  ✻  condition |  | 0.006 |  | 8.707e  -6 |  | 0.001 |  | 6.418e +10 |  | 16.750 |  |
| presentation order + session + standard duration + condition + presentation order  ✻  session + presentation order  ✻  standard duration + session  ✻  standard duration + standard duration  ✻  condition + presentation order  ✻  session  ✻  standard duration |  | 0.006 |  | 8.664e  -6 |  | 0.001 |  | 6.386e +10 |  | 3.645 |  |
| presentation order + session + standard duration + presentation order  ✻  session + presentation order  ✻  standard duration + session  ✻  standard duration + presentation order  ✻  session  ✻  standard duration |  | 0.006 |  | 7.453e  -6 |  | 0.001 |  | 5.493e +10 |  | 5.688 |  |
| presentation order + session + standard duration + condition + presentation order  ✻  session + presentation order  ✻  standard duration + session  ✻  standard duration + session  ✻  condition + standard duration  ✻  condition |  | 0.006 |  | 6.407e  -6 |  | 0.001 |  | 4.723e +10 |  | 14.768 |  |
| presentation order + session + standard duration + condition + presentation order  ✻  session + presentation order  ✻  standard duration + session  ✻  standard duration + session  ✻  condition + standard duration  ✻  condition + session  ✻  standard duration  ✻  condition |  | 0.006 |  | 2.364e  -6 |  | 3.925e  -4 |  | 1.743e +10 |  | 22.013 |  |
| presentation order + session + standard duration + condition + presentation order  ✻  session + presentation order  ✻  standard duration + session  ✻  standard duration + session  ✻  condition + standard duration  ✻  condition + presentation order  ✻  session  ✻  standard duration + session  ✻  standard duration  ✻  condition |  | 0.006 |  | 9.104e  -7 |  | 1.511e  -4 |  | 6.711e  +9 |  | 6.672 |  |
| presentation order + condition + presentation order  ✻  condition |  | 0.006 |  | 4.375e  -9 |  | 7.262e  -7 |  | 3.225e  +7 |  | 4.210 |  |
| presentation order + session + condition + presentation order  ✻  condition |  | 0.006 |  | 1.088e  -9 |  | 1.806e  -7 |  | 8.021e  +6 |  | 2.937 |  |
| presentation order + standard duration + condition + presentation order  ✻  condition |  | 0.006 |  | 7.632e -10 |  | 1.267e  -7 |  | 5.625e  +6 |  | 7.046 |  |
| presentation order + session + condition + presentation order  ✻  condition + session  ✻  condition |  | 0.006 |  | 3.132e -10 |  | 5.199e  -8 |  | 2.308e  +6 |  | 3.404 |  |
| presentation order + session + condition + presentation order  ✻  session + presentation order  ✻  condition |  | 0.006 |  | 2.708e -10 |  | 4.495e  -8 |  | 1.996e  +6 |  | 6.177 |  |
| presentation order + session + standard duration + condition + presentation order  ✻  condition |  | 0.006 |  | 1.980e -10 |  | 3.288e  -8 |  | 1.460e  +6 |  | 9.094 |  |
| presentation order + condition |  | 0.006 |  | 1.941e -10 |  | 3.222e  -8 |  | 1.431e  +6 |  | 3.171 |  |
| presentation order + standard duration + condition + presentation order  ✻  condition + standard duration  ✻  condition |  | 0.006 |  | 1.708e -10 |  | 2.835e  -8 |  | 1.259e  +6 |  | 3.578 |  |
| presentation order |  | 0.006 |  | 8.502e -11 |  | 1.411e  -8 |  | 626667.249 |  | 1.571 |  |
| presentation order + session + condition + presentation order  ✻  session + presentation order  ✻  condition + session  ✻  condition |  | 0.006 |  | 7.548e -11 |  | 1.253e  -8 |  | 556308.268 |  | 4.022 |  |
| presentation order + session + standard duration + condition + presentation order  ✻  condition + session  ✻  condition |  | 0.006 |  | 7.014e -11 |  | 1.164e  -8 |  | 516962.477 |  | 12.412 |  |
| presentation order + session + standard duration + condition + presentation order  ✻  condition + standard duration  ✻  condition |  | 0.006 |  | 6.466e -11 |  | 1.073e  -8 |  | 476607.654 |  | 32.974 |  |
| presentation order + session + standard duration + condition + session  ✻  standard duration + presentation order  ✻  condition |  | 0.006 |  | 6.152e -11 |  | 1.021e  -8 |  | 453437.713 |  | 8.432 |  |
| presentation order + session + standard duration + condition + presentation order  ✻  session + presentation order  ✻  condition |  | 0.006 |  | 4.675e -11 |  | 7.761e  -9 |  | 344588.299 |  | 4.442 |  |
| presentation order + session + condition |  | 0.006 |  | 4.657e -11 |  | 7.731e  -9 |  | 343279.140 |  | 1.831 |  |
| presentation order + standard duration + condition |  | 0.006 |  | 3.286e -11 |  | 5.455e  -9 |  | 242193.992 |  | 1.920 |  |
| presentation order + session + condition + presentation order  ✻  session + presentation order  ✻  condition + session  ✻  condition + presentation order  ✻  session  ✻  condition |  | 0.006 |  | 2.757e -11 |  | 4.577e  -9 |  | 203213.176 |  | 9.386 |  |
| presentation order + session |  | 0.006 |  | 2.151e -11 |  | 3.571e  -9 |  | 158547.593 |  | 2.513 |  |
| presentation order + session + condition + session  ✻  condition |  | 0.006 |  | 1.753e -11 |  | 2.911e  -9 |  | 129243.895 |  | 20.472 |  |
| presentation order + session + standard duration + condition + presentation order  ✻  condition + session  ✻  condition + standard duration  ✻  condition |  | 0.006 |  | 1.723e -11 |  | 2.860e  -9 |  | 126987.056 |  | 15.288 |  |
| presentation order + session + standard duration + condition + session  ✻  standard duration + presentation order  ✻  condition + session  ✻  condition |  | 0.006 |  | 1.673e -11 |  | 2.776e  -9 |  | 123278.799 |  | 5.259 |  |
| presentation order + session + standard duration + condition + session  ✻  standard duration + presentation order  ✻  condition + standard duration  ✻  condition |  | 0.006 |  | 1.598e -11 |  | 2.653e  -9 |  | 117798.519 |  | 8.689 |  |
| presentation order + session + standard duration + condition + presentation order  ✻  session + presentation order  ✻  condition + session  ✻  condition |  | 0.006 |  | 1.498e -11 |  | 2.487e  -9 |  | 110439.057 |  | 13.491 |  |
| presentation order + standard duration |  | 0.006 |  | 1.431e -11 |  | 2.375e  -9 |  | 105464.140 |  | 1.164 |  |
| presentation order + session + standard duration + condition + presentation order  ✻  session + session  ✻  standard duration + presentation order  ✻  condition |  | 0.006 |  | 1.396e -11 |  | 2.318e  -9 |  | 102932.487 |  | 5.896 |  |
| presentation order + session + standard duration + condition + presentation order  ✻  session + presentation order  ✻  condition + standard duration  ✻  condition |  | 0.006 |  | 1.374e -11 |  | 2.280e  -9 |  | 101248.220 |  | 23.345 |  |
| presentation order + session + condition + presentation order  ✻  session |  | 0.006 |  | 1.178e -11 |  | 1.955e  -9 |  | 86808.901 |  | 2.472 |  |
| presentation order + session + standard duration + condition |  | 0.006 |  | 8.321e -12 |  | 1.381e  -9 |  | 61331.379 |  | 2.217 |  |
| presentation order + standard duration + condition + standard duration  ✻  condition |  | 0.006 |  | 7.744e -12 |  | 1.286e  -9 |  | 57080.501 |  | 2.397 |  |
| presentation order + session + presentation order  ✻  session |  | 0.006 |  | 5.001e -12 |  | 8.302e -10 |  | 36864.618 |  | 2.123 |  |
| presentation order + session + standard duration + condition + presentation order  ✻  session + presentation order  ✻  condition + session  ✻  condition + presentation order  ✻  session  ✻  condition |  | 0.006 |  | 4.129e -12 |  | 6.854e -10 |  | 30432.685 |  | 4.170 |  |
| presentation order + session + standard duration + condition + presentation order  ✻  session + session  ✻  standard duration + presentation order  ✻  condition + session  ✻  condition |  | 0.006 |  | 4.102e -12 |  | 6.810e -10 |  | 30235.777 |  | 4.730 |  |
| presentation order + session + standard duration |  | 0.006 |  | 3.879e -12 |  | 6.439e -10 |  | 28590.913 |  | 4.929 |  |
| presentation order + session + standard duration + condition + presentation order  ✻  session + presentation order  ✻  condition + session  ✻  condition + standard duration  ✻  condition |  | 0.006 |  | 3.804e -12 |  | 6.315e -10 |  | 28039.790 |  | 13.962 |  |
| presentation order + session + standard duration + condition + session  ✻  standard duration + presentation order  ✻  condition + session  ✻  condition + standard duration  ✻  condition |  | 0.006 |  | 3.754e -12 |  | 6.231e -10 |  | 27667.554 |  | 6.331 |  |
| presentation order + session + condition + presentation order  ✻  session + session  ✻  condition |  | 0.006 |  | 3.546e -12 |  | 5.886e -10 |  | 26135.115 |  | 3.576 |  |
| presentation order + session + standard duration + condition + presentation order  ✻  session + session  ✻  standard duration + presentation order  ✻  condition + standard duration  ✻  condition |  | 0.006 |  | 3.329e -12 |  | 5.527e -10 |  | 24540.553 |  | 6.648 |  |
| presentation order + session + standard duration + condition + session  ✻  standard duration |  | 0.006 |  | 2.663e -12 |  | 4.420e -10 |  | 19625.724 |  | 8.077 |  |
| presentation order + session + standard duration + condition + session  ✻  condition |  | 0.006 |  | 2.650e -12 |  | 4.399e -10 |  | 19532.556 |  | 5.785 |  |
| presentation order + session + standard duration + condition + standard duration  ✻  condition |  | 0.006 |  | 2.266e -12 |  | 3.761e -10 |  | 16699.802 |  | 9.324 |  |
| presentation order + session + standard duration + condition + presentation order  ✻  session |  | 0.006 |  | 1.983e -12 |  | 3.291e -10 |  | 14614.922 |  | 2.746 |  |
| presentation order + session + standard duration + condition + presentation order  ✻  session + session  ✻  standard duration + presentation order  ✻  condition + session  ✻  condition + presentation order  ✻  session  ✻  condition |  | 0.006 |  | 1.341e -12 |  | 2.225e -10 |  | 9881.015 |  | 8.367 |  |
| presentation order + session + standard duration + condition + session  ✻  standard duration + presentation order  ✻  condition + session  ✻  condition + standard duration  ✻  condition + session  ✻  standard duration  ✻  condition |  | 0.006 |  | 1.248e -12 |  | 2.072e -10 |  | 9201.963 |  | 7.495 |  |
| presentation order + session + standard duration + presentation order  ✻  session |  | 0.006 |  | 1.152e -12 |  | 1.913e -10 |  | 8493.340 |  | 19.131 |  |
| presentation order + session + standard duration + session  ✻  standard duration |  | 0.006 |  | 1.049e -12 |  | 1.741e -10 |  | 7731.577 |  | 2.522 |  |
| presentation order + session + standard duration + condition + presentation order  ✻  session + presentation order  ✻  condition + session  ✻  condition + standard duration  ✻  condition + presentation order  ✻  session  ✻  condition |  | 0.006 |  | 9.860e -13 |  | 1.637e -10 |  | 7267.328 |  | 5.853 |  |
| presentation order + session + standard duration + condition + presentation order  ✻  session + session  ✻  standard duration + presentation order  ✻  condition + session  ✻  condition + standard duration  ✻  condition |  | 0.006 |  | 9.039e -13 |  | 1.500e -10 |  | 6662.372 |  | 6.089 |  |
| presentation order + session + standard duration + condition + session  ✻  standard duration + session  ✻  condition |  | 0.006 |  | 8.835e -13 |  | 1.467e -10 |  | 6512.227 |  | 10.944 |  |
| presentation order + session + standard duration + condition + presentation order  ✻  session + session  ✻  standard duration |  | 0.006 |  | 6.451e -13 |  | 1.071e -10 |  | 4755.035 |  | 11.121 |  |
| presentation order + session + standard duration + condition + presentation order  ✻  session + session  ✻  condition |  | 0.006 |  | 6.402e -13 |  | 1.063e -10 |  | 4718.440 |  | 6.160 |  |
| presentation order + session + standard duration + condition + session  ✻  condition + standard duration  ✻  condition |  | 0.006 |  | 6.393e -13 |  | 1.061e -10 |  | 4712.088 |  | 6.179 |  |
| presentation order + session + standard duration + condition + session  ✻  standard duration + standard duration  ✻  condition |  | 0.006 |  | 6.257e -13 |  | 1.039e -10 |  | 4612.133 |  | 4.865 |  |
| presentation order + session + standard duration + condition + presentation order  ✻  session + standard duration  ✻  condition |  | 0.006 |  | 4.987e -13 |  | 8.278e -11 |  | 3675.461 |  | 6.974 |  |
| presentation order + session + standard duration + condition + presentation order  ✻  session + session  ✻  standard duration + presentation order  ✻  condition + session  ✻  condition + standard duration  ✻  condition + presentation order  ✻  session  ✻  condition |  | 0.006 |  | 3.706e -13 |  | 6.153e -11 |  | 2731.857 |  | 11.772 |  |
| presentation order + session + standard duration + condition + presentation order  ✻  session + session  ✻  standard duration + session  ✻  condition |  | 0.006 |  | 3.660e -13 |  | 6.076e -11 |  | 2697.890 |  | 42.046 |  |
| presentation order + session + standard duration + condition + presentation order  ✻  session + session  ✻  standard duration + presentation order  ✻  condition + session  ✻  condition + standard duration  ✻  condition + session  ✻  standard duration  ✻  condition |  | 0.006 |  | 3.037e -13 |  | 5.042e -11 |  | 2238.706 |  | 8.311 |  |
| presentation order + session + standard duration + presentation order  ✻  session + session  ✻  standard duration |  | 0.006 |  | 2.353e -13 |  | 3.905e -11 |  | 1734.044 |  | 2.215 |  |
| presentation order + session + standard duration + condition + presentation order  ✻  session + session  ✻  standard duration + standard duration  ✻  condition |  | 0.006 |  | 2.094e -13 |  | 3.477e -11 |  | 1543.793 |  | 31.648 |  |
| presentation order + session + standard duration + condition + session  ✻  standard duration + session  ✻  condition + standard duration  ✻  condition |  | 0.006 |  | 1.815e -13 |  | 3.013e -11 |  | 1337.994 |  | 4.416 |  |
| presentation order + session + standard duration + condition + presentation order  ✻  session + session  ✻  condition + standard duration  ✻  condition |  | 0.006 |  | 1.629e -13 |  | 2.704e -11 |  | 1200.674 |  | 9.399 |  |
| presentation order + session + standard duration + condition + presentation order  ✻  session + session  ✻  standard duration + presentation order  ✻  condition + session  ✻  condition + standard duration  ✻  condition + presentation order  ✻  session  ✻  condition + session  ✻  standard duration  ✻  condition |  | 0.006 |  | 9.893e -14 |  | 1.642e -11 |  | 729.152 |  | 10.469 |  |
| presentation order + session + standard duration + condition + session  ✻  standard duration + session  ✻  condition + standard duration  ✻  condition + session  ✻  standard duration  ✻  condition |  | 0.006 |  | 5.457e -14 |  | 9.059e -12 |  | 402.245 |  | 4.327 |  |
| presentation order + session + standard duration + condition + presentation order  ✻  session + session  ✻  standard duration + session  ✻  condition + standard duration  ✻  condition |  | 0.006 |  | 4.291e -14 |  | 7.123e -12 |  | 316.261 |  | 9.153 |  |
| presentation order + session + standard duration + condition + presentation order  ✻  session + session  ✻  standard duration + session  ✻  condition + standard duration  ✻  condition + session  ✻  standard duration  ✻  condition |  | 0.006 |  | 1.389e -14 |  | 2.306e -12 |  | 102.407 |  | 5.949 |  |
| condition |  | 0.006 |  | 1.900e -16 |  | 3.154e -14 |  | 1.401 |  | 1.573 |  |
| session + condition |  | 0.006 |  | 4.475e -17 |  | 7.429e -15 |  | 0.330 |  | 3.133 |  |
| standard duration + condition |  | 0.006 |  | 3.275e -17 |  | 5.437e -15 |  | 0.241 |  | 2.071 |  |
| session |  | 0.006 |  | 3.166e -17 |  | 5.256e -15 |  | 0.233 |  | 1.230 |  |
| standard duration |  | 0.006 |  | 2.389e -17 |  | 3.965e -15 |  | 0.176 |  | 1.404 |  |
| session + condition + session  ✻  condition |  | 0.006 |  | 1.199e -17 |  | 1.991e -15 |  | 0.088 |  | 2.458 |  |
| standard duration + condition + standard duration  ✻  condition |  | 0.006 |  | 8.022e -18 |  | 1.332e -15 |  | 0.059 |  | 8.213 |  |
| session + standard duration + condition |  | 0.006 |  | 7.925e -18 |  | 1.316e -15 |  | 0.058 |  | 2.346 |  |
| session + standard duration |  | 0.006 |  | 5.757e -18 |  | 9.556e -16 |  | 0.042 |  | 3.636 |  |
| session + standard duration + condition + session  ✻  condition |  | 0.006 |  | 2.166e -18 |  | 3.595e -16 |  | 0.016 |  | 2.508 |  |
| session + standard duration + condition + session  ✻  standard duration |  | 0.006 |  | 2.135e -18 |  | 3.543e -16 |  | 0.016 |  | 3.032 |  |
| session + standard duration + condition + standard duration  ✻  condition |  | 0.006 |  | 1.903e -18 |  | 3.159e -16 |  | 0.014 |  | 3.995 |  |
| session + standard duration + session  ✻  standard duration |  | 0.006 |  | 1.749e -18 |  | 2.903e -16 |  | 0.013 |  | 8.009 |  |
| session + standard duration + condition + session  ✻  standard duration + session  ✻  condition |  | 0.006 |  | 6.142e -19 |  | 1.020e -16 |  | 0.005 |  | 4.006 |  |
| session + standard duration + condition + session  ✻  condition + standard duration  ✻  condition |  | 0.006 |  | 5.282e -19 |  | 8.768e -17 |  | 0.004 |  | 2.980 |  |
| session + standard duration + condition + session  ✻  standard duration + standard duration  ✻  condition |  | 0.006 |  | 5.192e -19 |  | 8.618e -17 |  | 0.004 |  | 4.385 |  |
| session + standard duration + condition + session  ✻  standard duration + session  ✻  condition + standard duration  ✻  condition |  | 0.006 |  | 1.431e -19 |  | 2.375e -17 |  | 0.001 |  | 3.503 |  |
| session + standard duration + condition + session  ✻  standard duration + session  ✻  condition + standard duration  ✻  condition + session  ✻  standard duration  ✻  condition |  | 0.006 |  | 4.766e -20 |  | 7.912e -18 |  | 3.513e  -4 |  | 5.977 |  |
|  | | | | | | | | | | | |
| *Note.*  All models include subject | | | | | | | | | | | |

**Table 3** Model comparisons for biases in Experiment 2. *Note.* P(M) represents the prior for each modal. P(M|data) represents how much the data were explained by the model. BF_M_ represents how much the data was likely under the alternative hypothesis than the null hypothesis. BF_10_ represents the Bayesian factor. Note that all models include subject as a variable.

| **Model Comparison** | | | | | | | | | | | |
| --- | --- | --- | --- | --- | --- | --- | --- | --- | --- | --- | --- |
| **Models** | | **P(M)** | | **P(M\|data)** | | **BF _M_** | | **BF _10_** | | **error %** | |
| Null model (incl. subject) |  | 0.006 |  | 4.859e -11 |  | 8.066e -9 |  | 1.000 |  |  |  |
| presentation order + session + standard duration + condition |  | 0.006 |  | 0.071 |  | 12.706 |  | 1.463e +9 |  | 2.364 |  |
| presentation order + session + standard duration + condition + session  ✻  condition |  | 0.006 |  | 0.063 |  | 11.172 |  | 1.298e +9 |  | 2.644 |  |
| presentation order + standard duration + condition |  | 0.006 |  | 0.050 |  | 8.820 |  | 1.038e +9 |  | 2.222 |  |
| presentation order + session + standard duration + condition + presentation order  ✻  standard duration |  | 0.006 |  | 0.043 |  | 7.524 |  | 8.924e +8 |  | 2.388 |  |
| presentation order + session + standard duration + condition + presentation order  ✻  standard duration + session  ✻  condition |  | 0.006 |  | 0.041 |  | 7.109 |  | 8.452e +8 |  | 8.330 |  |
| presentation order + session + condition |  | 0.006 |  | 0.036 |  | 6.274 |  | 7.495e +8 |  | 2.848 |  |
| presentation order + session + standard duration + condition + session  ✻  standard duration |  | 0.006 |  | 0.034 |  | 5.764 |  | 6.906e +8 |  | 4.379 |  |
| presentation order + standard duration + condition + presentation order  ✻  standard duration |  | 0.006 |  | 0.033 |  | 5.695 |  | 6.826e +8 |  | 11.059 |  |
| presentation order + session + condition + session  ✻  condition |  | 0.006 |  | 0.031 |  | 5.309 |  | 6.379e +8 |  | 2.283 |  |
| presentation order + condition |  | 0.006 |  | 0.028 |  | 4.819 |  | 5.806e +8 |  | 2.277 |  |
| presentation order + session + standard duration + condition + session  ✻  standard duration + session  ✻  condition |  | 0.006 |  | 0.026 |  | 4.443 |  | 5.365e +8 |  | 2.570 |  |
| presentation order + session + standard duration + condition + presentation order  ✻  standard duration + session  ✻  standard duration |  | 0.006 |  | 0.023 |  | 3.850 |  | 4.665e +8 |  | 3.307 |  |
| presentation order + session + standard duration + condition + presentation order  ✻  session |  | 0.006 |  | 0.021 |  | 3.609 |  | 4.379e +8 |  | 2.987 |  |
| presentation order + session + standard duration + condition + presentation order  ✻  session + session  ✻  condition |  | 0.006 |  | 0.020 |  | 3.464 |  | 4.207e +8 |  | 10.435 |  |
| presentation order + session + standard duration + condition + standard duration  ✻  condition |  | 0.006 |  | 0.020 |  | 3.389 |  | 4.118e +8 |  | 2.874 |  |
| presentation order + session + standard duration + condition + session  ✻  condition + standard duration  ✻  condition |  | 0.006 |  | 0.018 |  | 3.049 |  | 3.712e +8 |  | 3.246 |  |
| presentation order + session + standard duration + condition + presentation order  ✻  standard duration + session  ✻  standard duration + session  ✻  condition |  | 0.006 |  | 0.018 |  | 2.973 |  | 3.621e +8 |  | 5.280 |  |
| presentation order + session + standard duration + condition + presentation order  ✻  condition |  | 0.006 |  | 0.017 |  | 2.892 |  | 3.524e +8 |  | 2.759 |  |
| presentation order + session + standard duration + condition + presentation order  ✻  session + presentation order  ✻  standard duration |  | 0.006 |  | 0.017 |  | 2.801 |  | 3.415e +8 |  | 16.043 |  |
| presentation order + session + standard duration + condition + presentation order  ✻  session + presentation order  ✻  standard duration + session  ✻  condition |  | 0.006 |  | 0.016 |  | 2.662 |  | 3.248e +8 |  | 34.348 |  |
| presentation order + session + standard duration + condition + presentation order  ✻  condition + session  ✻  condition |  | 0.006 |  | 0.015 |  | 2.569 |  | 3.137e +8 |  | 5.617 |  |
| presentation order + standard duration + condition + standard duration  ✻  condition |  | 0.006 |  | 0.014 |  | 2.405 |  | 2.939e +8 |  | 3.129 |  |
| presentation order + session + standard duration + condition + presentation order  ✻  standard duration + standard duration  ✻  condition |  | 0.006 |  | 0.014 |  | 2.346 |  | 2.868e +8 |  | 3.329 |  |
| presentation order + standard duration + condition + presentation order  ✻  condition |  | 0.006 |  | 0.013 |  | 2.142 |  | 2.622e +8 |  | 5.440 |  |
| presentation order + session + standard duration + condition + presentation order  ✻  standard duration + session  ✻  condition + standard duration  ✻  condition |  | 0.006 |  | 0.012 |  | 2.061 |  | 2.524e +8 |  | 3.902 |  |
| presentation order + session + standard duration + condition + presentation order  ✻  standard duration + presentation order  ✻  condition |  | 0.006 |  | 0.012 |  | 2.048 |  | 2.508e +8 |  | 3.288 |  |
| presentation order + session + condition + presentation order  ✻  session |  | 0.006 |  | 0.011 |  | 1.795 |  | 2.202e +8 |  | 2.644 |  |
| presentation order + session + standard duration + condition + presentation order  ✻  session + session  ✻  standard duration |  | 0.006 |  | 0.010 |  | 1.754 |  | 2.152e +8 |  | 7.209 |  |
| presentation order + session + standard duration + condition + session  ✻  standard duration + standard duration  ✻  condition |  | 0.006 |  | 0.010 |  | 1.664 |  | 2.042e +8 |  | 5.661 |  |
| presentation order + session + condition + presentation order  ✻  condition |  | 0.006 |  | 0.010 |  | 1.598 |  | 1.963e +8 |  | 3.800 |  |
| presentation order + session + standard duration + condition + presentation order  ✻  standard duration + presentation order  ✻  condition + session  ✻  condition |  | 0.006 |  | 0.009 |  | 1.513 |  | 1.859e +8 |  | 3.820 |  |
| presentation order + standard duration + condition + presentation order  ✻  standard duration + standard duration  ✻  condition |  | 0.006 |  | 0.009 |  | 1.511 |  | 1.856e +8 |  | 3.322 |  |
| presentation order + session + condition + presentation order  ✻  session + session  ✻  condition |  | 0.006 |  | 0.009 |  | 1.508 |  | 1.853e +8 |  | 5.243 |  |
| presentation order + session + standard duration + condition + session  ✻  standard duration + presentation order  ✻  condition |  | 0.006 |  | 0.008 |  | 1.322 |  | 1.627e +8 |  | 4.096 |  |
| presentation order + session + condition + presentation order  ✻  condition + session  ✻  condition |  | 0.006 |  | 0.008 |  | 1.321 |  | 1.625e +8 |  | 2.953 |  |
| presentation order + session + standard duration + condition + presentation order  ✻  session + presentation order  ✻  standard duration + session  ✻  standard duration |  | 0.006 |  | 0.008 |  | 1.260 |  | 1.551e +8 |  | 15.329 |  |
| presentation order + standard duration + condition + presentation order  ✻  standard duration + presentation order  ✻  condition |  | 0.006 |  | 0.008 |  | 1.255 |  | 1.544e +8 |  | 3.111 |  |
| presentation order + session + standard duration + condition + presentation order  ✻  session + session  ✻  standard duration + session  ✻  condition |  | 0.006 |  | 0.007 |  | 1.243 |  | 1.530e +8 |  | 3.125 |  |
| presentation order + session + standard duration + condition + session  ✻  standard duration + session  ✻  condition + standard duration  ✻  condition |  | 0.006 |  | 0.007 |  | 1.224 |  | 1.506e +8 |  | 3.584 |  |
| presentation order + condition + presentation order  ✻  condition |  | 0.006 |  | 0.007 |  | 1.146 |  | 1.411e +8 |  | 5.153 |  |
| presentation order + session + standard duration + condition + presentation order  ✻  session + standard duration  ✻  condition |  | 0.006 |  | 0.006 |  | 1.066 |  | 1.313e +8 |  | 6.285 |  |
| presentation order + session + standard duration + condition + session  ✻  standard duration + presentation order  ✻  condition + session  ✻  condition |  | 0.006 |  | 0.006 |  | 1.039 |  | 1.280e +8 |  | 3.881 |  |
| presentation order + session + standard duration + condition + presentation order  ✻  standard duration + session  ✻  standard duration + standard duration  ✻  condition |  | 0.006 |  | 0.006 |  | 0.982 |  | 1.210e +8 |  | 4.970 |  |
| presentation order + session + standard duration + condition + presentation order  ✻  standard duration + session  ✻  standard duration + presentation order  ✻  condition |  | 0.006 |  | 0.005 |  | 0.883 |  | 1.089e +8 |  | 3.608 |  |
| presentation order + session + standard duration + condition + presentation order  ✻  session + presentation order  ✻  standard duration + session  ✻  standard duration + session  ✻  condition |  | 0.006 |  | 0.005 |  | 0.877 |  | 1.081e +8 |  | 4.793 |  |
| presentation order + session + standard duration + condition + presentation order  ✻  standard duration + session  ✻  standard duration + session  ✻  condition + standard duration  ✻  condition |  | 0.006 |  | 0.005 |  | 0.864 |  | 1.066e +8 |  | 7.099 |  |
| presentation order + session + standard duration + condition + presentation order  ✻  session + presentation order  ✻  condition |  | 0.006 |  | 0.005 |  | 0.859 |  | 1.060e +8 |  | 3.230 |  |
| presentation order + session + standard duration + condition + presentation order  ✻  session + session  ✻  standard duration + presentation order  ✻  condition + session  ✻  condition + presentation order  ✻  session  ✻  condition |  | 0.006 |  | 0.005 |  | 0.857 |  | 1.058e +8 |  | 68.168 |  |
| presentation order + session + standard duration + condition + presentation order  ✻  standard duration + session  ✻  standard duration + presentation order  ✻  condition + session  ✻  condition |  | 0.006 |  | 0.005 |  | 0.817 |  | 1.008e +8 |  | 4.938 |  |
| presentation order + session + standard duration + condition + presentation order  ✻  session + presentation order  ✻  standard duration + standard duration  ✻  condition |  | 0.006 |  | 0.005 |  | 0.795 |  | 9.806e +7 |  | 17.173 |  |
| presentation order + session + standard duration + condition + presentation order  ✻  session + session  ✻  condition + standard duration  ✻  condition |  | 0.006 |  | 0.005 |  | 0.785 |  | 9.692e +7 |  | 3.606 |  |
| presentation order + session + standard duration + condition + presentation order  ✻  condition + standard duration  ✻  condition |  | 0.006 |  | 0.005 |  | 0.783 |  | 9.662e +7 |  | 4.612 |  |
| presentation order + session + standard duration + condition + presentation order  ✻  session + presentation order  ✻  condition + session  ✻  condition + presentation order  ✻  session  ✻  condition |  | 0.006 |  | 0.005 |  | 0.761 |  | 9.396e +7 |  | 13.149 |  |
| presentation order + session + standard duration + condition + presentation order  ✻  session + presentation order  ✻  condition + session  ✻  condition |  | 0.006 |  | 0.004 |  | 0.718 |  | 8.865e +7 |  | 7.667 |  |
| presentation order + session + standard duration + condition + presentation order  ✻  session + presentation order  ✻  standard duration + presentation order  ✻  condition |  | 0.006 |  | 0.004 |  | 0.673 |  | 8.306e +7 |  | 11.482 |  |
| presentation order + session + standard duration + condition + presentation order  ✻  condition + session  ✻  condition + standard duration  ✻  condition |  | 0.006 |  | 0.004 |  | 0.651 |  | 8.033e +7 |  | 5.303 |  |
| presentation order + standard duration + condition + presentation order  ✻  condition + standard duration  ✻  condition |  | 0.006 |  | 0.004 |  | 0.630 |  | 7.781e +7 |  | 7.859 |  |
| presentation order + session + standard duration + condition + presentation order  ✻  session + session  ✻  standard duration + standard duration  ✻  condition |  | 0.006 |  | 0.003 |  | 0.542 |  | 6.700e +7 |  | 4.245 |  |
| presentation order + session + standard duration + condition + presentation order  ✻  standard duration + presentation order  ✻  condition + standard duration  ✻  condition |  | 0.006 |  | 0.003 |  | 0.516 |  | 6.374e +7 |  | 5.407 |  |
| presentation order + session + standard duration + condition + presentation order  ✻  session + presentation order  ✻  standard duration + presentation order  ✻  condition + session  ✻  condition |  | 0.006 |  | 0.003 |  | 0.507 |  | 6.267e +7 |  | 6.552 |  |
| presentation order + session + standard duration + condition + session  ✻  standard duration + presentation order  ✻  condition + standard duration  ✻  condition |  | 0.006 |  | 0.003 |  | 0.503 |  | 6.219e +7 |  | 23.757 |  |
| presentation order + session + condition + presentation order  ✻  session + presentation order  ✻  condition |  | 0.006 |  | 0.003 |  | 0.488 |  | 6.037e +7 |  | 2.954 |  |
| presentation order + session + standard duration + condition + presentation order  ✻  session + presentation order  ✻  standard duration + session  ✻  standard duration + presentation order  ✻  session  ✻  standard duration |  | 0.006 |  | 0.003 |  | 0.486 |  | 6.011e +7 |  | 3.717 |  |
| presentation order + session + standard duration + condition + presentation order  ✻  session + presentation order  ✻  standard duration + session  ✻  condition + standard duration  ✻  condition |  | 0.006 |  | 0.003 |  | 0.482 |  | 5.955e +7 |  | 3.138 |  |
| presentation order + session + standard duration + condition + presentation order  ✻  session + session  ✻  standard duration + session  ✻  condition + standard duration  ✻  condition |  | 0.006 |  | 0.003 |  | 0.453 |  | 5.606e +7 |  | 5.508 |  |
| presentation order + session + standard duration + condition + session  ✻  standard duration + session  ✻  condition + standard duration  ✻  condition + session  ✻  standard duration  ✻  condition |  | 0.006 |  | 0.003 |  | 0.452 |  | 5.584e +7 |  | 10.913 |  |
| presentation order + session + standard duration + condition + presentation order  ✻  session + session  ✻  standard duration + presentation order  ✻  condition |  | 0.006 |  | 0.003 |  | 0.425 |  | 5.254e +7 |  | 3.945 |  |
| presentation order + session + standard duration + condition + presentation order  ✻  session + presentation order  ✻  standard duration + session  ✻  standard duration + session  ✻  condition + presentation order  ✻  session  ✻  standard duration |  | 0.006 |  | 0.003 |  | 0.424 |  | 5.249e +7 |  | 3.382 |  |
| presentation order + session + standard duration + condition + presentation order  ✻  standard duration + presentation order  ✻  condition + session  ✻  condition + standard duration  ✻  condition |  | 0.006 |  | 0.002 |  | 0.384 |  | 4.745e +7 |  | 2.585 |  |
| presentation order + session + standard duration + condition + presentation order  ✻  session + presentation order  ✻  standard duration + presentation order  ✻  condition + session  ✻  condition + presentation order  ✻  session  ✻  condition |  | 0.006 |  | 0.002 |  | 0.381 |  | 4.715e +7 |  | 4.634 |  |
| presentation order + standard duration + condition + presentation order  ✻  standard duration + presentation order  ✻  condition + standard duration  ✻  condition |  | 0.006 |  | 0.002 |  | 0.354 |  | 4.380e +7 |  | 3.440 |  |
| presentation order + session + condition + presentation order  ✻  session + presentation order  ✻  condition + session  ✻  condition |  | 0.006 |  | 0.002 |  | 0.343 |  | 4.244e +7 |  | 3.768 |  |
| presentation order + session + standard duration + condition + presentation order  ✻  session + presentation order  ✻  standard duration + session  ✻  standard duration + standard duration  ✻  condition |  | 0.006 |  | 0.002 |  | 0.338 |  | 4.187e +7 |  | 7.121 |  |
| presentation order + session + standard duration + condition + presentation order  ✻  session + session  ✻  standard duration + presentation order  ✻  condition + session  ✻  condition |  | 0.006 |  | 0.002 |  | 0.330 |  | 4.083e +7 |  | 4.277 |  |
| presentation order + session + standard duration + condition + session  ✻  standard duration + presentation order  ✻  condition + session  ✻  condition + standard duration  ✻  condition |  | 0.006 |  | 0.002 |  | 0.309 |  | 3.829e +7 |  | 4.953 |  |
| presentation order + session + condition + presentation order  ✻  session + presentation order  ✻  condition + session  ✻  condition + presentation order  ✻  session  ✻  condition |  | 0.006 |  | 0.002 |  | 0.304 |  | 3.758e +7 |  | 6.775 |  |
| presentation order + session + standard duration + condition + presentation order  ✻  standard duration + session  ✻  standard duration + session  ✻  condition + standard duration  ✻  condition + session  ✻  standard duration  ✻  condition |  | 0.006 |  | 0.002 |  | 0.304 |  | 3.756e +7 |  | 7.969 |  |
| presentation order + session + standard duration + condition + presentation order  ✻  session + presentation order  ✻  condition + standard duration  ✻  condition |  | 0.006 |  | 0.002 |  | 0.298 |  | 3.682e +7 |  | 11.694 |  |
| presentation order + session + standard duration + condition + presentation order  ✻  session + presentation order  ✻  standard duration + session  ✻  standard duration + presentation order  ✻  condition |  | 0.006 |  | 0.002 |  | 0.277 |  | 3.423e +7 |  | 6.465 |  |
| presentation order + session + standard duration + condition + presentation order  ✻  session + presentation order  ✻  standard duration + session  ✻  standard duration + session  ✻  condition + standard duration  ✻  condition |  | 0.006 |  | 0.002 |  | 0.267 |  | 3.307e +7 |  | 7.905 |  |
| presentation order + session + standard duration + condition + presentation order  ✻  standard duration + session  ✻  standard duration + presentation order  ✻  condition + standard duration  ✻  condition |  | 0.006 |  | 0.002 |  | 0.265 |  | 3.284e +7 |  | 4.989 |  |
| presentation order + session + standard duration + condition + presentation order  ✻  session + presentation order  ✻  condition + session  ✻  condition + standard duration  ✻  condition |  | 0.006 |  | 0.001 |  | 0.239 |  | 2.964e +7 |  | 17.271 |  |
| presentation order + session + standard duration + condition + presentation order  ✻  session + presentation order  ✻  standard duration + session  ✻  standard duration + presentation order  ✻  condition + session  ✻  condition |  | 0.006 |  | 0.001 |  | 0.239 |  | 2.956e +7 |  | 7.664 |  |
| presentation order + session + standard duration + condition + presentation order  ✻  standard duration + session  ✻  standard duration + presentation order  ✻  condition + session  ✻  condition + standard duration  ✻  condition |  | 0.006 |  | 0.001 |  | 0.222 |  | 2.750e +7 |  | 5.353 |  |
| presentation order + session + standard duration + condition + presentation order  ✻  session + presentation order  ✻  condition + session  ✻  condition + standard duration  ✻  condition + presentation order  ✻  session  ✻  condition |  | 0.006 |  | 0.001 |  | 0.199 |  | 2.465e +7 |  | 5.244 |  |
| presentation order + session + standard duration + condition + presentation order  ✻  session + presentation order  ✻  standard duration + session  ✻  standard duration + presentation order  ✻  condition + session  ✻  condition + presentation order  ✻  session  ✻  condition |  | 0.006 |  | 0.001 |  | 0.193 |  | 2.395e +7 |  | 8.040 |  |
| presentation order + session + standard duration + condition + presentation order  ✻  session + presentation order  ✻  standard duration + presentation order  ✻  condition + standard duration  ✻  condition |  | 0.006 |  | 0.001 |  | 0.186 |  | 2.307e +7 |  | 14.123 |  |
| presentation order + standard duration + condition + presentation order  ✻  standard duration + presentation order  ✻  condition + standard duration  ✻  condition + presentation order  ✻  standard duration  ✻  condition |  | 0.006 |  | 0.001 |  | 0.167 |  | 2.075e +7 |  | 19.480 |  |
| presentation order + session + standard duration + condition + presentation order  ✻  standard duration + presentation order  ✻  condition + standard duration  ✻  condition + presentation order  ✻  standard duration  ✻  condition |  | 0.006 |  | 9.764e  -4 |  | 0.162 |  | 2.010e +7 |  | 4.583 |  |
| presentation order + session + standard duration |  | 0.006 |  | 9.561e  -4 |  | 0.159 |  | 1.968e +7 |  | 1.740 |  |
| presentation order + session + standard duration + condition + presentation order  ✻  session + presentation order  ✻  standard duration + session  ✻  standard duration + presentation order  ✻  condition + presentation order  ✻  session  ✻  standard duration |  | 0.006 |  | 9.233e  -4 |  | 0.153 |  | 1.900e +7 |  | 12.301 |  |
| presentation order + session + standard duration + condition + presentation order  ✻  session + presentation order  ✻  standard duration + session  ✻  standard duration + standard duration  ✻  condition + presentation order  ✻  session  ✻  standard duration |  | 0.006 |  | 9.139e  -4 |  | 0.152 |  | 1.881e +7 |  | 6.245 |  |
| presentation order + session + standard duration + condition + presentation order  ✻  standard duration + presentation order  ✻  condition + session  ✻  condition + standard duration  ✻  condition + presentation order  ✻  standard duration  ✻  condition |  | 0.006 |  | 8.528e  -4 |  | 0.142 |  | 1.755e +7 |  | 7.272 |  |
| presentation order + session + standard duration + condition + presentation order  ✻  session + session  ✻  standard duration + session  ✻  condition + standard duration  ✻  condition + session  ✻  standard duration  ✻  condition |  | 0.006 |  | 8.085e  -4 |  | 0.134 |  | 1.664e +7 |  | 11.640 |  |
| presentation order + standard duration |  | 0.006 |  | 8.052e  -4 |  | 0.134 |  | 1.657e +7 |  | 2.003 |  |
| presentation order + session + standard duration + condition + presentation order  ✻  session + presentation order  ✻  standard duration + presentation order  ✻  condition + session  ✻  condition + standard duration  ✻  condition |  | 0.006 |  | 7.819e  -4 |  | 0.130 |  | 1.609e +7 |  | 5.106 |  |
| presentation order + session + standard duration + condition + presentation order  ✻  session + session  ✻  standard duration + presentation order  ✻  condition + standard duration  ✻  condition |  | 0.006 |  | 7.629e  -4 |  | 0.127 |  | 1.570e +7 |  | 4.704 |  |
| presentation order + session + standard duration + condition + presentation order  ✻  session + presentation order  ✻  standard duration + session  ✻  standard duration + presentation order  ✻  condition + session  ✻  condition + presentation order  ✻  session  ✻  standard duration |  | 0.006 |  | 7.601e  -4 |  | 0.126 |  | 1.564e +7 |  | 9.362 |  |
| presentation order + session + standard duration + condition + presentation order  ✻  session + presentation order  ✻  standard duration + session  ✻  standard duration + session  ✻  condition + standard duration  ✻  condition + presentation order  ✻  session  ✻  standard duration |  | 0.006 |  | 7.443e  -4 |  | 0.124 |  | 1.532e +7 |  | 5.929 |  |
| presentation order + session + standard duration + condition + presentation order  ✻  session + presentation order  ✻  standard duration + session  ✻  standard duration + session  ✻  condition + standard duration  ✻  condition + session  ✻  standard duration  ✻  condition |  | 0.006 |  | 7.299e  -4 |  | 0.121 |  | 1.502e +7 |  | 23.812 |  |
| presentation order + session + standard duration + condition + session  ✻  standard duration + presentation order  ✻  condition + session  ✻  condition + standard duration  ✻  condition + session  ✻  standard duration  ✻  condition |  | 0.006 |  | 6.807e  -4 |  | 0.113 |  | 1.401e +7 |  | 8.421 |  |
| presentation order + session + standard duration + condition + presentation order  ✻  session + presentation order  ✻  standard duration + presentation order  ✻  condition + session  ✻  condition + standard duration  ✻  condition + presentation order  ✻  session  ✻  condition |  | 0.006 |  | 6.778e  -4 |  | 0.113 |  | 1.395e +7 |  | 4.242 |  |
| presentation order + session + standard duration + condition + presentation order  ✻  session + presentation order  ✻  standard duration + session  ✻  standard duration + presentation order  ✻  condition + session  ✻  condition + presentation order  ✻  session  ✻  standard duration + presentation order  ✻  session  ✻  condition |  | 0.006 |  | 6.439e  -4 |  | 0.107 |  | 1.325e +7 |  | 8.070 |  |
| presentation order + session |  | 0.006 |  | 6.325e  -4 |  | 0.105 |  | 1.302e +7 |  | 1.735 |  |
| presentation order + session + standard duration + condition + presentation order  ✻  session + session  ✻  standard duration + presentation order  ✻  condition + session  ✻  condition + standard duration  ✻  condition |  | 0.006 |  | 6.043e  -4 |  | 0.100 |  | 1.244e +7 |  | 7.302 |  |
| presentation order + session + standard duration + presentation order  ✻  standard duration |  | 0.006 |  | 5.749e  -4 |  | 0.095 |  | 1.183e +7 |  | 4.052 |  |
| presentation order + session + standard duration + condition + presentation order  ✻  session + session  ✻  standard duration + presentation order  ✻  condition + session  ✻  condition + standard duration  ✻  condition + presentation order  ✻  session  ✻  condition |  | 0.006 |  | 5.721e  -4 |  | 0.095 |  | 1.177e +7 |  | 7.304 |  |
| presentation order |  | 0.006 |  | 5.478e  -4 |  | 0.091 |  | 1.127e +7 |  | 1.502 |  |
| presentation order + session + standard duration + condition + presentation order  ✻  standard duration + session  ✻  standard duration + presentation order  ✻  condition + session  ✻  condition + standard duration  ✻  condition + session  ✻  standard duration  ✻  condition |  | 0.006 |  | 5.096e  -4 |  | 0.085 |  | 1.049e +7 |  | 7.692 |  |
| presentation order + session + standard duration + condition + presentation order  ✻  standard duration + session  ✻  standard duration + presentation order  ✻  condition + standard duration  ✻  condition + presentation order  ✻  standard duration  ✻  condition |  | 0.006 |  | 4.984e  -4 |  | 0.083 |  | 1.026e +7 |  | 5.748 |  |
| presentation order + standard duration + presentation order  ✻  standard duration |  | 0.006 |  | 4.661e  -4 |  | 0.077 |  | 9.592e +6 |  | 2.171 |  |
| presentation order + session + standard duration + condition + presentation order  ✻  session + presentation order  ✻  standard duration + session  ✻  standard duration + presentation order  ✻  condition + standard duration  ✻  condition |  | 0.006 |  | 4.532e  -4 |  | 0.075 |  | 9.327e +6 |  | 5.037 |  |
| presentation order + session + standard duration + session  ✻  standard duration |  | 0.006 |  | 4.055e  -4 |  | 0.067 |  | 8.346e +6 |  | 2.455 |  |
| presentation order + session + standard duration + condition + presentation order  ✻  session + presentation order  ✻  standard duration + session  ✻  standard duration + presentation order  ✻  condition + session  ✻  condition + standard duration  ✻  condition + presentation order  ✻  session  ✻  condition |  | 0.006 |  | 3.869e  -4 |  | 0.064 |  | 7.964e +6 |  | 6.566 |  |
| presentation order + session + standard duration + condition + presentation order  ✻  standard duration + session  ✻  standard duration + presentation order  ✻  condition + session  ✻  condition + standard duration  ✻  condition + presentation order  ✻  standard duration  ✻  condition |  | 0.006 |  | 3.729e  -4 |  | 0.062 |  | 7.674e +6 |  | 3.995 |  |
| presentation order + session + standard duration + condition + presentation order  ✻  session + presentation order  ✻  standard duration + session  ✻  standard duration + presentation order  ✻  condition + session  ✻  condition + standard duration  ✻  condition |  | 0.006 |  | 3.479e  -4 |  | 0.058 |  | 7.161e +6 |  | 5.876 |  |
| presentation order + session + standard duration + condition + presentation order  ✻  session + presentation order  ✻  standard duration + presentation order  ✻  condition + standard duration  ✻  condition + presentation order  ✻  standard duration  ✻  condition |  | 0.006 |  | 3.306e  -4 |  | 0.055 |  | 6.804e +6 |  | 10.058 |  |
| presentation order + session + standard duration + presentation order  ✻  session |  | 0.006 |  | 3.085e  -4 |  | 0.051 |  | 6.350e +6 |  | 4.338 |  |
| presentation order + session + standard duration + condition + presentation order  ✻  session + presentation order  ✻  standard duration + session  ✻  standard duration + session  ✻  condition + standard duration  ✻  condition + presentation order  ✻  session  ✻  standard duration + session  ✻  standard duration  ✻  condition |  | 0.006 |  | 2.964e  -4 |  | 0.049 |  | 6.100e +6 |  | 15.162 |  |
| presentation order + session + standard duration + presentation order  ✻  standard duration + session  ✻  standard duration |  | 0.006 |  | 2.658e  -4 |  | 0.044 |  | 5.470e +6 |  | 2.906 |  |
| presentation order + session + standard duration + condition + presentation order  ✻  session + presentation order  ✻  standard duration + presentation order  ✻  condition + session  ✻  condition + standard duration  ✻  condition + presentation order  ✻  standard duration  ✻  condition |  | 0.006 |  | 2.397e  -4 |  | 0.040 |  | 4.933e +6 |  | 4.780 |  |
| presentation order + session + standard duration + condition + presentation order  ✻  session + presentation order  ✻  standard duration + session  ✻  standard duration + presentation order  ✻  condition + session  ✻  condition + standard duration  ✻  condition + presentation order  ✻  session  ✻  standard duration |  | 0.006 |  | 2.009e  -4 |  | 0.033 |  | 4.135e +6 |  | 6.230 |  |
| presentation order + session + standard duration + condition + presentation order  ✻  session + presentation order  ✻  standard duration + presentation order  ✻  condition + session  ✻  condition + standard duration  ✻  condition + presentation order  ✻  session  ✻  condition + presentation order  ✻  standard duration  ✻  condition |  | 0.006 |  | 1.988e  -4 |  | 0.033 |  | 4.091e +6 |  | 5.994 |  |
| presentation order + session + standard duration + condition + presentation order  ✻  session + presentation order  ✻  standard duration + session  ✻  standard duration + presentation order  ✻  condition + standard duration  ✻  condition + presentation order  ✻  session  ✻  standard duration |  | 0.006 |  | 1.917e  -4 |  | 0.032 |  | 3.945e +6 |  | 3.657 |  |
| presentation order + session + standard duration + condition + presentation order  ✻  session + session  ✻  standard duration + presentation order  ✻  condition + session  ✻  condition + standard duration  ✻  condition + presentation order  ✻  session  ✻  condition + session  ✻  standard duration  ✻  condition |  | 0.006 |  | 1.905e  -4 |  | 0.032 |  | 3.921e +6 |  | 15.018 |  |
| presentation order + session + standard duration + presentation order  ✻  session + presentation order  ✻  standard duration |  | 0.006 |  | 1.762e  -4 |  | 0.029 |  | 3.626e +6 |  | 3.956 |  |
| presentation order + session + presentation order  ✻  session |  | 0.006 |  | 1.689e  -4 |  | 0.028 |  | 3.475e +6 |  | 1.324 |  |
| presentation order + session + standard duration + condition + presentation order  ✻  session + session  ✻  standard duration + presentation order  ✻  condition + session  ✻  condition + standard duration  ✻  condition + session  ✻  standard duration  ✻  condition |  | 0.006 |  | 1.685e  -4 |  | 0.028 |  | 3.468e +6 |  | 4.279 |  |
| presentation order + session + standard duration + condition + presentation order  ✻  session + presentation order  ✻  standard duration + session  ✻  standard duration + presentation order  ✻  condition + session  ✻  condition + standard duration  ✻  condition + presentation order  ✻  session  ✻  standard duration + presentation order  ✻  session  ✻  condition |  | 0.006 |  | 1.554e  -4 |  | 0.026 |  | 3.198e +6 |  | 4.511 |  |
| presentation order + session + standard duration + condition + presentation order  ✻  session + presentation order  ✻  standard duration + session  ✻  standard duration + presentation order  ✻  condition + session  ✻  condition + standard duration  ✻  condition + presentation order  ✻  session  ✻  condition + presentation order  ✻  standard duration  ✻  condition |  | 0.006 |  | 1.362e  -4 |  | 0.023 |  | 2.804e +6 |  | 8.041 |  |
| presentation order + session + standard duration + condition + presentation order  ✻  standard duration + session  ✻  standard duration + presentation order  ✻  condition + session  ✻  condition + standard duration  ✻  condition + presentation order  ✻  standard duration  ✻  condition + session  ✻  standard duration  ✻  condition |  | 0.006 |  | 1.352e  -4 |  | 0.022 |  | 2.782e +6 |  | 9.061 |  |
| presentation order + session + standard duration + condition + presentation order  ✻  session + presentation order  ✻  standard duration + session  ✻  standard duration + presentation order  ✻  condition + session  ✻  condition + standard duration  ✻  condition + session  ✻  standard duration  ✻  condition |  | 0.006 |  | 1.275e  -4 |  | 0.021 |  | 2.623e +6 |  | 9.176 |  |
| presentation order + session + standard duration + condition + presentation order  ✻  session + presentation order  ✻  standard duration + session  ✻  standard duration + presentation order  ✻  condition + standard duration  ✻  condition + presentation order  ✻  standard duration  ✻  condition |  | 0.006 |  | 1.270e  -4 |  | 0.021 |  | 2.613e +6 |  | 4.928 |  |
| presentation order + session + standard duration + condition + presentation order  ✻  session + presentation order  ✻  standard duration + session  ✻  standard duration + presentation order  ✻  condition + session  ✻  condition + standard duration  ✻  condition + presentation order  ✻  standard duration  ✻  condition |  | 0.006 |  | 1.255e  -4 |  | 0.021 |  | 2.584e +6 |  | 5.851 |  |
| presentation order + session + standard duration + condition + presentation order  ✻  session + presentation order  ✻  standard duration + session  ✻  standard duration + presentation order  ✻  condition + session  ✻  condition + standard duration  ✻  condition + presentation order  ✻  session  ✻  condition + session  ✻  standard duration  ✻  condition |  | 0.006 |  | 1.215e  -4 |  | 0.020 |  | 2.500e +6 |  | 10.197 |  |
| presentation order + session + standard duration + presentation order  ✻  session + session  ✻  standard duration |  | 0.006 |  | 1.204e  -4 |  | 0.020 |  | 2.477e +6 |  | 2.991 |  |
| presentation order + session + standard duration + presentation order  ✻  session + presentation order  ✻  standard duration + session  ✻  standard duration |  | 0.006 |  | 7.756e  -5 |  | 0.013 |  | 1.596e +6 |  | 9.025 |  |
| presentation order + session + standard duration + condition + presentation order  ✻  session + presentation order  ✻  standard duration + session  ✻  standard duration + presentation order  ✻  condition + standard duration  ✻  condition + presentation order  ✻  session  ✻  standard duration + presentation order  ✻  standard duration  ✻  condition |  | 0.006 |  | 7.118e  -5 |  | 0.012 |  | 1.465e +6 |  | 11.744 |  |
| presentation order + session + standard duration + condition + presentation order  ✻  session + presentation order  ✻  standard duration + session  ✻  standard duration + presentation order  ✻  condition + session  ✻  condition + standard duration  ✻  condition + presentation order  ✻  session  ✻  standard duration + presentation order  ✻  session  ✻  condition + session  ✻  standard duration  ✻  condition |  | 0.006 |  | 6.786e  -5 |  | 0.011 |  | 1.397e +6 |  | 7.925 |  |
| presentation order + session + standard duration + condition + presentation order  ✻  session + presentation order  ✻  standard duration + session  ✻  standard duration + presentation order  ✻  condition + session  ✻  condition + standard duration  ✻  condition + presentation order  ✻  session  ✻  standard duration + session  ✻  standard duration  ✻  condition |  | 0.006 |  | 6.016e  -5 |  | 0.010 |  | 1.238e +6 |  | 7.389 |  |
| presentation order + session + standard duration + condition + presentation order  ✻  session + presentation order  ✻  standard duration + session  ✻  standard duration + presentation order  ✻  condition + session  ✻  condition + standard duration  ✻  condition + presentation order  ✻  session  ✻  standard duration + presentation order  ✻  standard duration  ✻  condition |  | 0.006 |  | 5.972e  -5 |  | 0.010 |  | 1.229e +6 |  | 9.378 |  |
| presentation order + session + standard duration + condition + presentation order  ✻  session + presentation order  ✻  standard duration + session  ✻  standard duration + presentation order  ✻  condition + session  ✻  condition + standard duration  ✻  condition + presentation order  ✻  session  ✻  standard duration + presentation order  ✻  session  ✻  condition + presentation order  ✻  standard duration  ✻  condition |  | 0.006 |  | 5.247e  -5 |  | 0.009 |  | 1.080e +6 |  | 7.910 |  |
| presentation order + session + standard duration + condition + presentation order  ✻  session + presentation order  ✻  standard duration + session  ✻  standard duration + presentation order  ✻  condition + session  ✻  condition + standard duration  ✻  condition + presentation order  ✻  standard duration  ✻  condition + session  ✻  standard duration  ✻  condition |  | 0.006 |  | 4.193e  -5 |  | 0.007 |  | 862992.521 |  | 7.896 |  |
| presentation order + session + standard duration + presentation order  ✻  session + presentation order  ✻  standard duration + session  ✻  standard duration + presentation order  ✻  session  ✻  standard duration |  | 0.006 |  | 3.965e  -5 |  | 0.007 |  | 815928.564 |  | 8.242 |  |
| presentation order + session + standard duration + condition + presentation order  ✻  session + presentation order  ✻  standard duration + session  ✻  standard duration + presentation order  ✻  condition + session  ✻  condition + standard duration  ✻  condition + presentation order  ✻  session  ✻  condition + presentation order  ✻  standard duration  ✻  condition + session  ✻  standard duration  ✻  condition |  | 0.006 |  | 3.162e  -5 |  | 0.005 |  | 650665.711 |  | 6.356 |  |
| presentation order + session + standard duration + condition + presentation order  ✻  session + presentation order  ✻  standard duration + session  ✻  standard duration + presentation order  ✻  condition + session  ✻  condition + standard duration  ✻  condition + presentation order  ✻  session  ✻  standard duration + presentation order  ✻  standard duration  ✻  condition + session  ✻  standard duration  ✻  condition |  | 0.006 |  | 1.671e  -5 |  | 0.003 |  | 343813.489 |  | 6.129 |  |
| presentation order + session + standard duration + condition + presentation order  ✻  session + presentation order  ✻  standard duration + session  ✻  standard duration + presentation order  ✻  condition + session  ✻  condition + standard duration  ✻  condition + presentation order  ✻  session  ✻  standard duration + presentation order  ✻  session  ✻  condition + presentation order  ✻  standard duration  ✻  condition + session  ✻  standard duration  ✻  condition |  | 0.006 |  | 1.650e  -5 |  | 0.003 |  | 339591.014 |  | 7.359 |  |
| presentation order + session + standard duration + condition + presentation order  ✻  session + presentation order  ✻  standard duration + session  ✻  standard duration + presentation order  ✻  condition + session  ✻  condition + standard duration  ✻  condition + presentation order  ✻  session  ✻  standard duration + presentation order  ✻  session  ✻  condition + presentation order  ✻  standard duration  ✻  condition + session  ✻  standard duration  ✻  condition + presentation order  ✻  session  ✻  standard duration  ✻  condition |  | 0.006 |  | 1.291e  -5 |  | 0.002 |  | 265655.016 |  | 16.890 |  |
| condition |  | 0.006 |  | 6.137e -10 |  | 1.019e -7 |  | 12.631 |  | 2.135 |  |
| standard duration + condition |  | 0.006 |  | 6.122e -10 |  | 1.016e -7 |  | 12.599 |  | 2.064 |  |
| session + standard duration + condition |  | 0.006 |  | 5.714e -10 |  | 9.486e -8 |  | 11.761 |  | 8.603 |  |
| session + condition |  | 0.006 |  | 4.661e -10 |  | 7.738e -8 |  | 9.593 |  | 1.181 |  |
| session + standard duration + condition + session  ✻  condition |  | 0.006 |  | 2.991e -10 |  | 4.966e -8 |  | 6.157 |  | 4.331 |  |
| session + condition + session  ✻  condition |  | 0.006 |  | 2.957e -10 |  | 4.909e -8 |  | 6.086 |  | 5.057 |  |
| session + standard duration + condition + session  ✻  standard duration |  | 0.006 |  | 1.868e -10 |  | 3.101e -8 |  | 3.845 |  | 3.247 |  |
| standard duration + condition + standard duration  ✻  condition |  | 0.006 |  | 1.686e -10 |  | 2.799e -8 |  | 3.470 |  | 3.782 |  |
| session + standard duration + condition + standard duration  ✻  condition |  | 0.006 |  | 1.412e -10 |  | 2.344e -8 |  | 2.906 |  | 4.558 |  |
| session + standard duration + condition + session  ✻  standard duration + session  ✻  condition |  | 0.006 |  | 1.161e -10 |  | 1.927e -8 |  | 2.388 |  | 4.891 |  |
| session + standard duration + condition + session  ✻  condition + standard duration  ✻  condition |  | 0.006 |  | 9.643e -11 |  | 1.601e -8 |  | 1.985 |  | 19.557 |  |
| session + standard duration + condition + session  ✻  standard duration + standard duration  ✻  condition |  | 0.006 |  | 5.329e -11 |  | 8.847e -9 |  | 1.097 |  | 4.582 |  |
| standard duration |  | 0.006 |  | 4.246e -11 |  | 7.048e -9 |  | 0.874 |  | 0.801 |  |
| session |  | 0.006 |  | 3.516e -11 |  | 5.836e -9 |  | 0.724 |  | 1.277 |  |
| session + standard duration |  | 0.006 |  | 3.325e -11 |  | 5.519e -9 |  | 0.684 |  | 1.772 |  |
| session + standard duration + condition + session  ✻  standard duration + session  ✻  condition + standard duration  ✻  condition |  | 0.006 |  | 3.276e -11 |  | 5.439e -9 |  | 0.674 |  | 3.879 |  |
| session + standard duration + session  ✻  standard duration |  | 0.006 |  | 1.221e -11 |  | 2.026e -9 |  | 0.251 |  | 2.289 |  |
| session + standard duration + condition + session  ✻  standard duration + session  ✻  condition + standard duration  ✻  condition + session  ✻  standard duration  ✻  condition |  | 0.006 |  | 1.196e -11 |  | 1.986e -9 |  | 0.246 |  | 16.055 |  |
|  | | | | | | | | | | | |
| *Note.*  All models include subject | | | | | | | | | | | |

**Table 4** Model comparisons for Weber fractions in Experiment 2. P(M) represents the prior for each modal. P(M|data) represents how much the data were explained by the model. BF_M_ represents how much the data was likely under the alternative hypothesis than the null hypothesis. BF_10_ represents the Bayesian factor. Note that all models include subject as a variable.
